# An expanded rating curve model to estimate river discharge during tidal influences across the progressive-mixed-standing wave systems

**DOI:** 10.1371/journal.pone.0225758

**Published:** 2019-12-18

**Authors:** Allan E. Jones, Amber K. Hardison, Ben R. Hodges, James W. McClelland, Kevan B. Moffett

**Affiliations:** 1 School of the Environment, Washington State University Vancouver, Vancouver, WA, United States of America; 2 Department of Marine Science, University of Texas Marine Science Institute, Port Aransas, TX, United States of America; 3 Department of Civil, Architectural and Environmental Engineering, University of Texas at Austin, Austin, TX, United States of America; University of the Chinese Academy of Sciences, CHINA

## Abstract

Empirically quantifying tidally-influenced river discharge is typically laborious, expensive, and subject to more uncertainty than estimation of upstream river discharge. The tidal stage-discharge relationship is not monotonic nor necessarily single-valued, so conventional stage-based river rating curves fail in the tidal zone. Herein, we propose an expanded rating curve method incorporating stage-rate-of-change to estimate river discharge under tidal influences across progressive, mixed, and standing waves. This simple and inexpensive method requires (1) stage from a pressure transducer, (2) flow direction from a tilt current meter, and (3) a series of ADP surveys at different flow rates for model calibration. The method was validated using excerpts from 12 tidal USGS gauging stations during baseflow conditions. USGS gauging stations model discharge using a different more complex and expensive method. Comparison of new and previous models resulted in good R^2^ correlations (min 0.62, mean 0.87 with S.D. 0.10, max 0.97). The method for modeling tidally-influenced discharge during baseflow conditions was applied *de novo* to eight intertidal stations in the Mission and Aransas Rivers, Texas, USA. In these same rivers, the model was further expanded to identify and estimate tidally-influenced stormflow discharges. The Mission and Aransas examples illustrated the potential scientific and management utility of the applied tidal rating curve method for isolating transient tidal influences and quantifying baseflow and storm discharges to sensitive coastal waters.

## 1. Introduction

Stage-discharge rating curves are useful and widely applied to estimate river discharge from inexpensive measurements of river surface height. A rating-curve model is a nonlinear stage-discharge regression that is calibrated for a specific river cross-section using current or discharge measurements paired with stage data [1–3]. Rating curves upstream of a tidal zone are (relatively) constant because local stage is typically a repeatable function of discharge [1]. However, in tidally-influenced systems stage is driven by a combination of riverine discharge and downstream tidal elevation. Thus, the dynamic stage-discharge relationship cannot be independent of tidal phase. To bypass issues associated with dynamic stage-discharge relationships, coastal rivers are typically gauged upstream of the tidal influence.

Although non-tidal river gauges are important for monitoring local discharge and for estimating net river discharge in a broad sense, multiple challenges arise when interpreting inland data as relevant to tidally-influenced rivers. First, relating non-tidal, upstream discharge to the river mouth assumes conservation of mass along the river channel, i.e., little significant gain or loss of river water between the gauging point and mouth. This condition may be difficult to meet in lowland systems where tidal influence can extend 10–100+ km inland [4–10]. Second, unidirectional river discharge measured upstream can only represent a net coastal discharge at daily or longer timescales. Otherwise, transient magnitudes and directions of diurnal and semidiurnal tidal discharge at the river mouth would not be captured.

There are a variety of reasons to seek methods for robustly estimating the time-varying discharge in a tidally-influenced river. Discharge and residence time within tidal systems play a vital role in determining the composition of instream and downstream estuarine biota, as well as influencing the global water budget and sediment supply to the ocean [11–15]. Long-term records of volumetric discharge describe riverine flow and transport conditions, and enable the investigation of nutrient and freshwater residence times in tidal river zones [16]. Tidal residence time dynamics will impact geochemical cycling processes and total maximum daily loads (TMDLs) through these critical coastal river reaches [17,18]. Furthermore, long-term discharge records aid in predictions of hyporheic exchange rates that control groundwater inputs of nitrogen (N) in these costal environments; understanding such exchange rates will further inform management and policy surrounding TMDLs [19,20]. Complete long-term tidal volumetric discharge records also inform increasingly precise relationships between terrestrial physical parameters and downstream ecology and biota over a range of timescales, from flashy, short-term event timescales to longer global climate trends [11,14,16,21,22].

At present, the two major approaches for quantifying tidal discharge are based on either continuous hydrodynamic empirical monitoring or detailed hydrodynamic modeling. To empirically estimate the discharge at tidally-influenced locations, the United States Geological Survey (USGS) employs the index velocity method [23,24]. This method uses a permanently installed acoustic Doppler velocity meter (ADVM) or ultrasonic velocity meter (UVM) to measure the velocities of suspended particles in the water column, which are then scaled to estimate mean channel velocity. The scaling algorithm is calibrated using a least-squares regression between site-specific empirical observations of index velocity (recorded by the site’s ADVM) and mean velocity (acquired via boat-mounted, downward-facing ADVM observations). The scaled mean velocity is then applied across the site’s cross-sectional area to model volumetric discharge. Although effective, this method requires permanent installation and maintenance of ADVM or UVM instrumentation. As of 2011, 470 stations operated and maintained by the USGS employed the index velocity method [23]. Other studies have also used the index velocity method, or similar scaling relationship [25,26].

Alternatively, the discharge from a river or estuary can be modeled using a carefully resolved, transient computer simulation [27–30]. Several studies have investigated methods to decrease the computational expense of tidal river modeling, by such methods as decreasing dimensionality [28] or employing a more conceptual architecture (e.g., conceptual reservoir-type models) [31]. Also, recent studies have used artificial neural networks (ANN) to characterize tidal river stage-discharge relationships [32]. ANNs provide strong hindcast discharge results, while still improving on forecasting and the ability of these networks to address conditions more extreme than their training sets [31,32]. However, such models (both multi-dimensional simulations and ANNs) require exceptional hardware and software capabilities, which may be impractical for every river mouth of interest.

Other methods used to model tidal river discharges include estimates from direct measurements of discharge [33,34], permanently installed horizontally-mounted acoustic instrumentation [35–37], empirical relationships derived from estuarine geometry [38], and one-dimensional tidal hydrodynamics models [39–41]. The goal of the tidal rating curve method developed herein is to provide empirically-based discharge estimates for tidal rivers using concepts and a calibration approach similar to classic river rating curve methods, but for less cost and effort than other methods.

## 2. Methods

### 2.1 Tidal influence on stage and discharge

**Fig 1:**
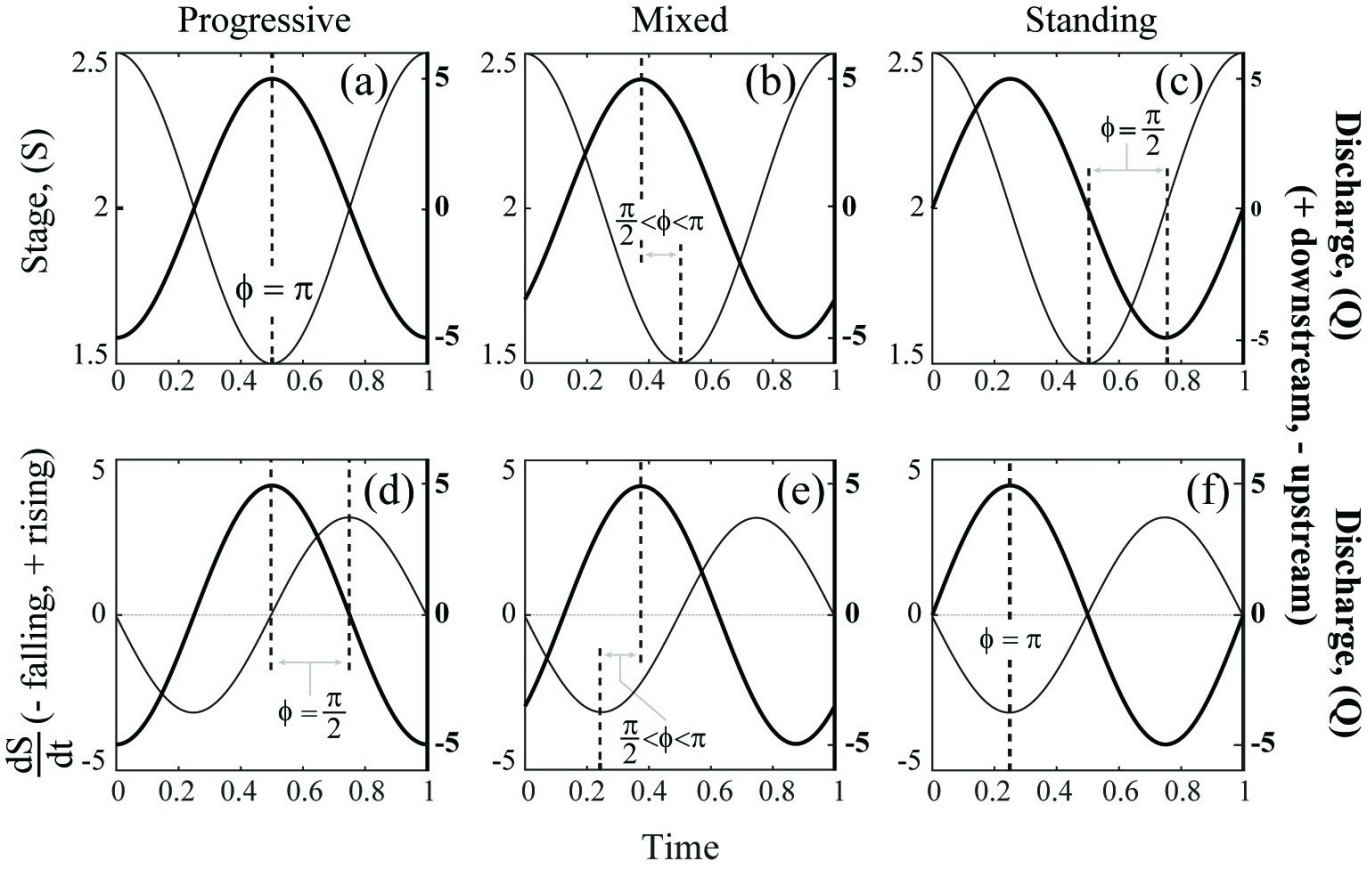
Conceptual schematic of progressive, mixed, and standing waveforms. Schematic of relationships between stage (*S*) and discharge (*Q*) (first row) and between stage-rate-of-change (*dS/dt*) and discharge (second row) through time, for progressive (a, d), mixed (b, e), and standing (c, f) waveforms. Thicker line and right y-axes represent *Q(t)*, thinner lines and left y-axes represent *S(t)* or *dS(t)/dt*. Absolute magnitude of phase offset (| ϕ |) between *S* and *Q* is the space between the dashed lines. The figure was composed using MATLAB from a hypothetical dataset.

To motivate the mathematical formulation, it is helpful to first consider the different patterns of influence that tides can exert on river stage and discharge. In contrast to the conventional monotonic stage-discharge relationship in a non-tidal reach, the tides allow development of a phase-shifted, time-varying relationship between stage and discharge. Although a tidally-influenced river will behave as a continuum, it is useful to broadly classify the stage-discharge phase relationships as standing, mixed, or progressive waves [8], as illustrated in Fig 1(A), 1(B), and 1(C), respectively.

The different wave behaviors arise through the interaction of geomorphology and tidal physics. For the simple ideal case of an infinitely long frictionless channel with constant cross-section, the tidal energy propagates upstream as a progressive wave [8]. In this waveform, river discharge to the sea will be greatest when stage is lowest, i.e., at low tide, when the river’s longitudinal free water surface slope is maximized (Fig 1A). Then, as tidal flow reverses, i.e., discharge toward the sea reduces and eventually flows upstream, water is backed up in the river channel, and stage rises until the highest stage corresponds with lowest (most negative) discharge. In this case, the maximum rate of change of stage actually occurs when river discharge (*Q*) is just reversing direction, i.e., neatly at *Q* = 0 (Fig 1D). Typically, only deep man-made shipping channels of constant cross-section promote propagation of progressive waveforms [8]. Conversely, in a short estuary, or a semi-enclosed body (i.e., a bay) effectively functioning as a storage basin, tidal energy should instead travel upstream via a standing wave [8]. In this case, slack water (zero discharge) aligns with high tide and low tide, and maximum tidal speeds occur around mean water level, when stage is changing most rapidly. For this standing wave case, *Q* and the change in stage (*dS/dt*) are neatly in phase-opposition (Fig 1F). Standing waves are more common in fjord or ria systems (i.e., drowned river valleys) [8]. Alluvial estuaries more commonly experience an intermediate set of conditions, characterized as a mixed waveform with phase-lagged offsets between stage and discharge (Fig 1B) and between discharge and stage-rate-of-change (Fig 1E) [8]. For a greater in-depth, analytic, and numeric discussion of tidal wave propagation see the works of [42,43] and others.

Each waveform in the progressive-mixed-standing continuum has a characteristic phase offset (ϕ) between tidal stage and discharge. As discussion of this topic predominantly originates with oceanography and estuarine hydrodynamics, standing, mixed, and progressive waves are typically discussed in the literature with the coordinate axes aligned with the direction of wave travel, i.e., positive upstream, resulting in offsets of ϕ = 0 for an ideal progressive wave to ϕ = $\frac{\pi }{2}$ for an ideal standing wave [8]. However, for this application to riverine science, and to allow river discharge to be positive when directed toward the sea (i.e., opposite the incoming tidal wave), we rotated our coordinate system by π. Thus, in our analysis, a progressive tidal wave traveling upstream has a phase offset relative to stage of ϕ = π and should exhibit a negative relationship between discharge and stage (Fig 1A). The phase offset of a standing wave in our coordinate system remains $\left|\frac{\pi }{2}\right|$, since phase offsets of $\frac{3\pi }{2}$ and $-\frac{\pi }{2}$ are equivalent (Fig 1C). A standing wave system should exhibit a negative relationship between discharge and stage-rate-of-change ($\frac{dS}{dt}$) (Fig 1F). Intermediate, mixed waves exhibit phase offsets of $\frac{\pi }{2}$ < | ϕ | < π in our coordinate system (Fig 1B).

### 2.2 Field sites and data collection

#### 2.2.1 Texas field sites

The tidal rating curve method requires the permanent installation of a pressure transducer and a tilt current meter (TCM) at each monitoring site for which discharge estimates are desired. Long-term records of the site’s stage, stage-rate-of-change ($\frac{dS}{dt}$), and thalweg velocity are paired with occasional acoustic Doppler profiler (ADP) discharge measurements for model calibration.

**Fig 2:**
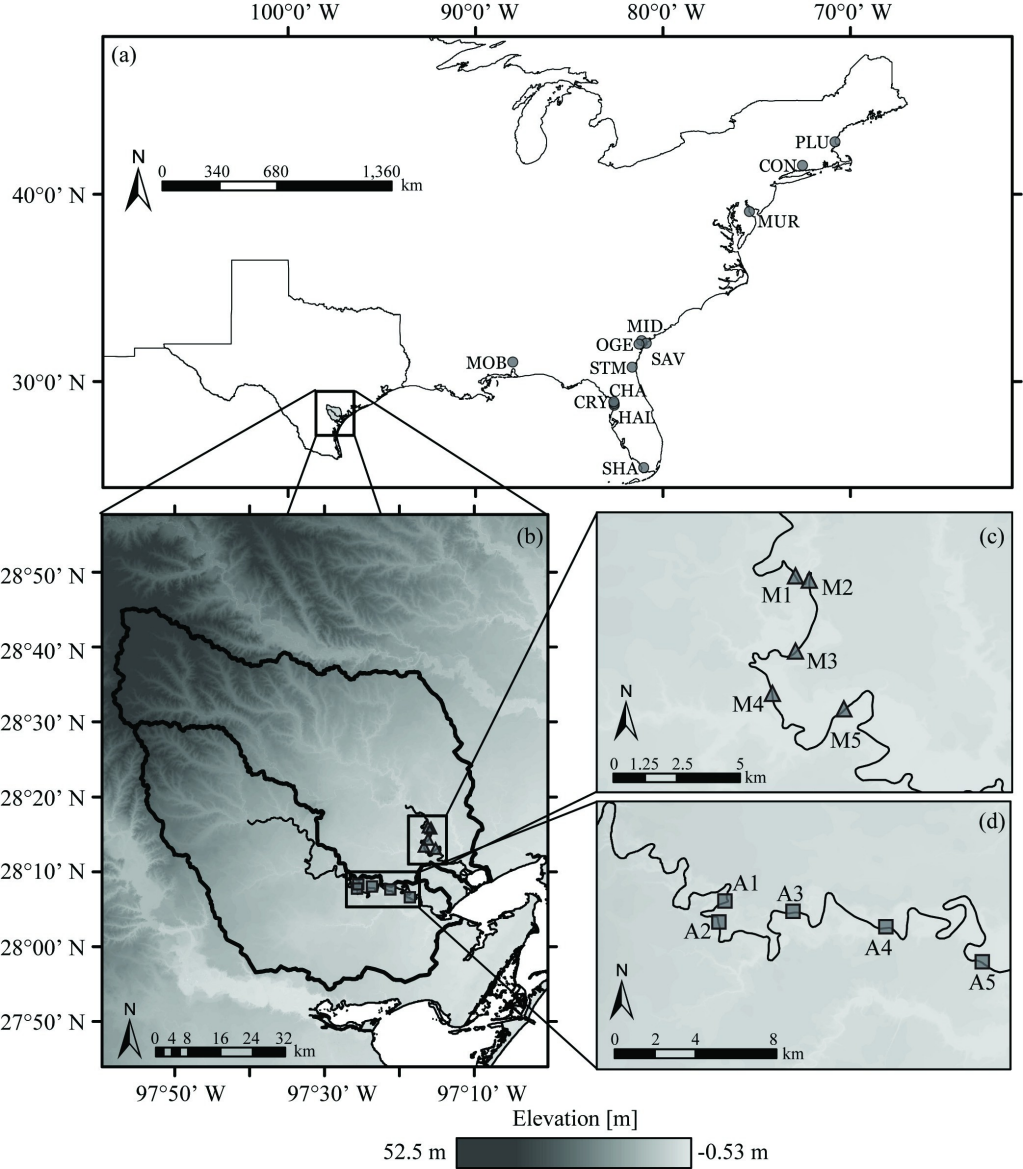
Locations of the tidal rating curve model validation and application sites. The 12 tidal USGS gauges available nationally with appropriate data for validation were located throughout the eastern US (circles in a) [51]. The ten original model application sites were on the Mission (triangles in b,c) and Aransas (squares in b, d) Rivers of the Texas Gulf Coastal plain, distributed throughout the tidal freshwater zone of each river [16]. Elevation data, watershed boundaries, and river lines were obtained from U.S. Geological Survey, National Geospatial Program [52]. The Texas state and U.S.A. country outline was obtained from the U.S. Census Bureau [53]. Both the National Geospatial Program and the U.S. Census Bureau provide public access to their data sets. Data visualized using ESRI ArcGIS software.

To test this approach *de novo* in the field, ten monitoring locations (Fig 2B), five in each of the Mission (Fig 2C) and Aransas Rivers (Fig 2D) recorded stage using a pressure datalogging sonde (LTC Levellogger Jr., Solinst, Georgetown, ON, CAN) and thalweg velocity using a tilt current meter (TCM; SeaHorse, OkeanoLog, North Falmouth, MA, USA) installed on the bed. From the stage observations, we calculated the rate of change of stage ($\frac{dS}{dt}$) as:

$$\frac{dS_{n}}{dt}=\frac{{Stage}_{n+1}-{Stage}_{n}}{{time}_{n+1}-{time}_{n}}$$ (1)

The field sampling interval (*dt*) of 15 minutes generated 96 samples daily, which was consistent with index velocity method recommendations of 50 to 120 samples every 12 to 13 hours to ensure sufficient resolution of the tidal cycle [24].

The Mission and Aransas Rivers (M-A) provided good locations at which to test the operational utility of the tidal rating curve methodology because of their challenging conditions. Their small tidal range, complex tidal signal, small baseflow, and extremely flashy flood events would all logically challenge the method’s ability to obtain a robust calibration on baseflow tidal periods and produce a continuous time series of discharge estimates across both baseflow and stormflow conditions. The tidal range is micro-tidal, at just 0.15 m, but the set of coastal bays forming the M-A Estuary system between the rivers and the Gulf of Mexico cause a highly convoluted tidal signal to reach the rivers and this study’s field measurement sites [18,21,44,45]. The M-A Rivers experience drought and flood extremes while lacking seasonal discharge regimes. The region’s sub-humid to semiarid-subtropical climate generates extreme hydrologic variability and strong seasonal oscillations in wind and temperature [45,46]. Elevated summer temperatures often result in summer evaporation exceeding local annual precipitation [18,45,47]. However, warm summer waters from the Gulf of Mexico and El Niño Southern Oscillation (ENSO) events promote storms, leading to flash flooding and freshets in coastal bays [14,18,21,22,45,48,49]. This typically baseflow, yet wide-ranging set of riverine conditions, when combined with tidal forces, provided an ideal test case for the new tidal rating curve method.

The Aransas watershed covers approximately 2,146 km^2^ of primarily cropland, while the Mission watershed spans approximately 2,675 km^2^ of shrub and forest land [18,45]. The minimal topographic relief of the M-A watersheds (Fig 2B) promotes long water residence times in the river channels [16] that impact the timing and magnitude of nutrient fluxes to, and hence the biological productivity of, downstream estuarine environments [10,13,14,18,21].

#### 2.2.2 USGS validation sites

To validate the new tidal rating curve model with third party, verified data, we applied the baseflow tidal rating curve model (Eq 1) to data from 12 USGS streamflow gauges in tidal settings and non-storm conditions (Fig 2A). To be useful for this validation, the USGS stations needed to report both discharge and stage. The 12 selected stations provided the most complete, USGS-verified, 15-minute sampling records of tidal discharge and stage available from July 2015 to July 2017. The data were obtained from the USGS National Water Information System (NWIS) [50].

The 12 USGS stations used for validation resided throughout the eastern United States (Fig 2A) and in several different ecological and climate zones (Table S7.1). The USGS stations extended from the Florida Everglades (Shark River) and southeastern plains (Mobile River) to the Northeastern temperate forests (Connecticut River). The stations also monitored a range of discharges, with the maximum discharge, averaged between July 2015 and July 2017, represented by the Mobile River, Alabama, of about 655 m^3^ s^-1^ and the minimum average of 0.91 m^3^ s^-1^ on the Halls River, Florida.

### 2.3 Phase analysis

To determine the standing, mixed, or progressive waveform for a given site, we performed a phase offset analysis. This analysis compared the timing of tidal discharge and stage to determine whether their sinusoids matched over time or were offset (e.g., peak discharge occurring with maximum stage). A Fast Fourier Transform (FFT) using the Discrete Fourier Transform (DFT) package of Matlab (Mathworks, Natick, MA, USA) returned the local semidiurnal and diurnal harmonics in both stage and discharge. Longer-term harmonics (e.g., annual or semiannual) were found to be nearly in-phase between stage and discharge signals. Since these longer harmonics did not elucidate standing, mixed, or progressive character, they were omitted from further analysis. The FFT determined the phases of stage, *ε*_*S*_, and discharge, *ε*_*Q*_, at each of the three significant, semidiurnal and diurnal, harmonic frequencies (*f*) at each station. We calculated the three frequency-specific phase offsets, ϕ_*f*_, at each station as:

$$\phi_{f}=|\varepsilon_{S,f}-\varepsilon_{Q,f}|$$ (2)

For each site analyzed, Eq 2 provided three nearly identical ϕ_*f*_ values, which were averaged to obtain the final characterization of each station’s stage-discharge phase offset (ϕ). The resulting offset defined each station’s dynamics as a standing (ϕ ≈ $\frac{\pi }{2}$), progressive (ϕ ≈ π), or mixed ($\frac{\pi }{2}$ < | ϕ | < π) waveform. Section B in the S1 File provides greater detail regarding FFT methods and results.

### 2.4 The tidal rating curve model

The first expansion we introduce to the classic river rating curve method to help enable estimation of tidal discharge is to incorporate into the model the river’s stage-rate-of-change, $\frac{dS}{dt}$. Because a given tidal stage paired with a negative $\frac{dS}{dt}$ value (e.g., ebb tide) describes a different tidal condition and discharge than when paired with a positive $\frac{dS}{dt}$ value (e.g., flood tide), the combination of stage and stage-rate-of-change data allow the oscillatory and transient effects of tides on stage and discharge during the tidal cycle to be accounted for. An additional modification of the classic approach empirically distinguishes stormflow (and its rising and falling limbs of a storm hydrograph) from tidal stage changes, which is a necessary condition for capturing both storm and tidal influences on a single system. Together, these two expansions of the river rating curve method permit estimation of continuous discharge time series for tidally-influenced river reaches using low-cost and simple empirical data.

#### 2.4.1 Modeling tidally-influenced discharge during baseflow

As per assessment of discharge-stage interactions across the continuum of progressive-mixed-standing wave types of river-tide interactions, river discharge (*Q*) during baseflow and tidal conditions should relate to a combination of stage (*S*) and $\frac{dS}{dt}$. The dominant waveform of a system determines whether discharge is more strongly related to *S* (i.e., progressive), $\frac{dS}{dt}$ (i.e., standing), or a combination (i.e., mixed). The classic river rating curve that relates *Q* monotonically to *S* takes the form *Q* = *P S*^*β*^ if zero flow corresponds with zero gauge height (i.e., zero intercept). For a given stage gauge location of interest, the rating curve constants *P* and *β* are calibrated to sparse flow measurements over a variety of conditions and *β* typically falls between 1 and 2 [3]. In some cases, a parabolic equation is used instead: *Q* = *a*_1_ *S* + *a*_2_ *S*^2^ [3]. To enable representation of mixed tidal waveform influences on river stage in a similarly simple additive framework, we adopted the parabolic representation and expanded it with two additional stage-rate-of-change terms. The resulting tidal rating curve model at time *t*_*i*_ is:

$$Q_{i}=k_{1}S_{i}^{2}+k_{2}S_{i}+k_{3}(\frac{dS_{i}}{dt}{)}^{2}+k_{4}\frac{{dS}_{i}}{dt}$$ (3)

Site-specific coefficients *k*_*1*_ through *k*_*4*_ encapsulate factors related to site-specific channel geometry, hydrologic, wave amplification, or damping behaviors, similarly to the *a*_*1*_ and *a*_*2*_ parameters of the classic parabolic river rating curve. Eq 3 does not include an intercept and so assumes zero flow occurs when stage is zero; this formulation is easily modified by addition of an intercept if appropriate for a specific setting or gauge.

Eq 3 contains two halves corresponding to progressive and standing wave endmembers of tidal systems. When linearly superimposed, these halves also represent mixed wave systems. The *k*_*1*_ and *k*_*2*_ terms reflect progressive wave conditions or non-tidal river conditions by relating discharge to stage, while terms *k*_*3*_ and *k*_*4*_ represent standing wave behavior by relating discharge to $\frac{dS}{dt}$. A linear relationship (i.e., using only coefficients *k*_*2*_ and *k*_*4*_) was tested and found to be inadequate to sufficiently estimate tidal discharge; second-order polynomials provided the next simplest and adequate model that was fully consistent with existing non-tidal rating curve methods (parabolic form).

To operationalize the tidal rating curve model for a site of interest, stage data need to be recorded continuously (e.g., by pressure transducer) and site-specific coefficients *k*_*1*_ through *k*_*4*_ must be calibrated. This calibration can be done using a limited set of Acoustic Doppler Profiler (ADP) measurements of discharge collected concurrently with stage data, just as calibration for the classic non-tidal river rating curve would be done. In the tidal case, ADP observations should be collected during different combinations of baseflow discharge and the primary tidal harmonics. The most efficient number of ADP surveys required for a given site will depend on its discharge and stage variability. Sets of ADP measurements also must be sufficiently long to observe temporal changes in stage, i.e., half a tidal cycle or more. Before relating discharge to *S* and $\frac{dS}{dt}$, a 3-hour moving average or other smoothing procedure is recommended to remove noise (e.g., wind) from the *S* and $\frac{dS}{dt}$ time series. For systems with semi-diurnal tidal influence, a 3-hour window represents a quarter of a tidal cycle and so is the largest averaging window possible without drastically influencing the $Q=f(S,\frac{dS}{dt})$ relationship. While the smoothing procedure may remove the instantaneous, outlier variation from observed S and $\frac{dS}{dt}$ time series, the procedure will uncover underlying harmonics that govern discharge. These underlying harmonics are necessary to properly calibrate the model coefficients, k_1_-k_4_. The ADP observations of *Q* are then regressed against their respective concurrent *S* and $\frac{dS}{dt}$ observations to calibrate the four model coefficients, *k*_*1*_, *k*_*2*_, *k*_*3*_, and *k*_*4*_, for the site of interest.

A final model-pruning step can be employed to help avoid over-fitting the $Q=f(S,\frac{dS}{dt})$ relationship with unneeded terms, e.g., in a fully progressive or fully standing wave setting. Given the four terms of the full model (Eq 1), 15 different candidate models exist for a given site that are combinations of one to four terms. Candidate models exhibiting insignificant *p* values (i.e., *p* > 0.05) for any of the calibrated parameters are first discarded, then the remaining model with the best empirical fit to the calibration data (highest R^2^) is selected as the tidal rating curve model for baseflow conditions.

#### 2.4.2 Identifying and modeling stormflow discharge

A rating curve designed for tidal rivers must accommodate both the riverine and tidal endmembers. Large increases in riverine discharge from runoff, snowmelt, or upstream flood events may overwhelm the local tidal character and emulate non-tidal riverine behavior [16]; Jones et al., *In Review*]. Identifying if tidal influence is still notable in the river can then become especially difficult as the early and late tails of storm hydrographs can mimic rising and falling tidal signals. For periods of unidirectional velocity, however, such as will occur in when runoff or flood events overwhelm the tidal signal in an otherwise tidal river reach, the complex relationship of Eq 3 is unnecessary. Instead a simple, area-scaled specific discharge model can reasonably approximate discharge during such unidirectional discharge events:

$$Q_{i}={a*v}_{i}*A(S_{i})$$ (4)

with velocity (*v*_*i*_) and stage (*S*_*i*_) observations at time *t*_*i*_, velocity scaling coefficient *a*, and cross-sectional area *A* as a function of stage. Depending on the shape of the channel cross-section, this model (Eq 4) can easily reproduce the classic linear or parabolic river rating curve forms [3]. For example, assuming a rectangular channel of width *w*: *A*(*S*_*i*_) *= w S*_*i*_, Eq 2 would represent a linear river rating curve model. For an isosceles trapezoidal channel with bed width *w* and side slope *m*: *A*(*S*_*i*_) *= w S*_*i*_ *+ m*^*-1*^ *S*_*i*_^*2*^ and so the model represents a parabolic river rating curve. *A*(*S*_*i*_) can be substituted by analysis of other regular geometries, if appropriate, or parametrized from cross-sectional channel survey data. Use of empirical cross-sectional channel survey data would align with the usual methods and degree of effort required by typical river gaging stations [54] and be fully comparable to the stage-area rating concept that forms part of the USGS index velocity method [23,24]. The coefficient, *a*, scales the measured velocity to reflect the average cross-sectional velocity, with *a* = 1 signifying a near match. The scaling coefficient is necessary to complete Eq 2 since point velocity observations may not describe discharge complexity throughout the riverine cross-section. Section 4.2 provides an in-depth discussion of the issues of point velocity observations. The area-scaled specific discharge model of (Eq 4) was adopted in this method as one of the simplest approaches available, requiring little to no calibration, and also well-suited to calculating discharge for unidirectional, high flows [1,54].

The velocity data for Eq 4 can be suitably obtained at high flows from an inexpensive tilt-current meter (TCM) [55,56]. This study provided the first TCM application to rivers, following on previous work with TCMs that has investigated the bottom circulation and sediment dynamics in the Gulf of Maine [57,58] and the current dynamics in the Mesquite, Aransas, and Copano Bays of Texas [59]. Raw TCM accelerometer data are recorded as X, Y, and Z coordinates, which then need calibration and normalization to produce local velocity data. Calibration tests for each TCM provide the maximum observable X, Y, and Z coordinates and baseline coordinate observations during zero-velocity conditions [55,56]. TCM field observations are then normalized within the range between the sensor’s maximum (i.e., TCM horizontal in the direction of flow) and zero-velocity endmembers. Submerged TCM field installations may also experience misalignment between observed (accelerometer) and ideal (primary flow direction) coordinate axes. In this study, axial misalignment was rectified using pitch, roll, and yaw rotational corrections similar to those used in eddy flux wind data processing. From the normalized and rotated TCM data, the angle-from-vertical is calculated and then converted to a velocity measurement using a flume-derived angle-velocity relationship [55,56]. Greater detail on TCM calibration and methods is provided in Section A in the S1 File.

Depending on the TCM technology used, there will be a lower limit to the sensitivity of the instrument’s embedded accelerometer. For example, for the Okeanolog SeaHorse TCMs used in this research, absolute velocities with magnitudes less than 2 cm s^-1^ had unreliable magnitudes [55,56]. However, the timings of directional oscillations due to tidal effects at flow magnitudes < 2 cm s^-1^ (i.e., positive vs. negative flows) were still sufficiently accurate to identify the timing of changes in discharge regimes for our study sites [57,58]. During storm flows, though, TCM velocity data greatly exceeded the instrument’s lower threshold and so were suitable to use as velocities in Eq 2.

#### 2.4.3 Piecewise combination of stormflow and tidal baseflow rating curve models

Combined, Eqs 1 and 3 represent a piecewise rating curve model appropriate for systems prone to both tide and flood influences. It remains to specify a threshold for transitioning between the two model pieces, the key characteristic of which must be to separate times when the location of interest is tidally-influenced, i.e., stage and discharge are not monotonically related, from times when the reach is experiencing unidirectional flow. In this first pilot of the new tidal rating curve method, we used the 90^th^ percentile of annual measured reach velocity as a simple empirical means of identifying the threshold above which stormflow occurred. Benefits of this simple threshold approach are that it easily adaptable—and should be adapted—on a site-specific basis. For example, at a gauging site with only microtidal influence in a watershed prone to frequent, moderately-sized floods, a lower percentile (e.g., 70^th^ or 80^th^) may be appropriate. For the very flashy discharge regime of extended minimal baseflow punctuated by large runoff events that characterized the Mission and Aransas Rivers tested in this research [16,18,21], the 90^th^ percentile of a year of TCM velocity measurements from a given monitoring site served well to identify the occurrence of high flow events.

## 3. Results

### 3.1 USGS validation sites

#### 3.1.1 Phase analysis

To assess whether this set of 12 USGS stations spanned the continuum of progressive-mixed-standing tidal wave influences that the tidal rating curve sought to be able to capture, we calculated the phase offset between the longest period of reported discharge and stage at each station between July 2015 and July 2017. Raw discharge observations from USGS stations were averaged using a 3-hour moving window to remove potential noise before applying the FFT.

**Fig 3:**
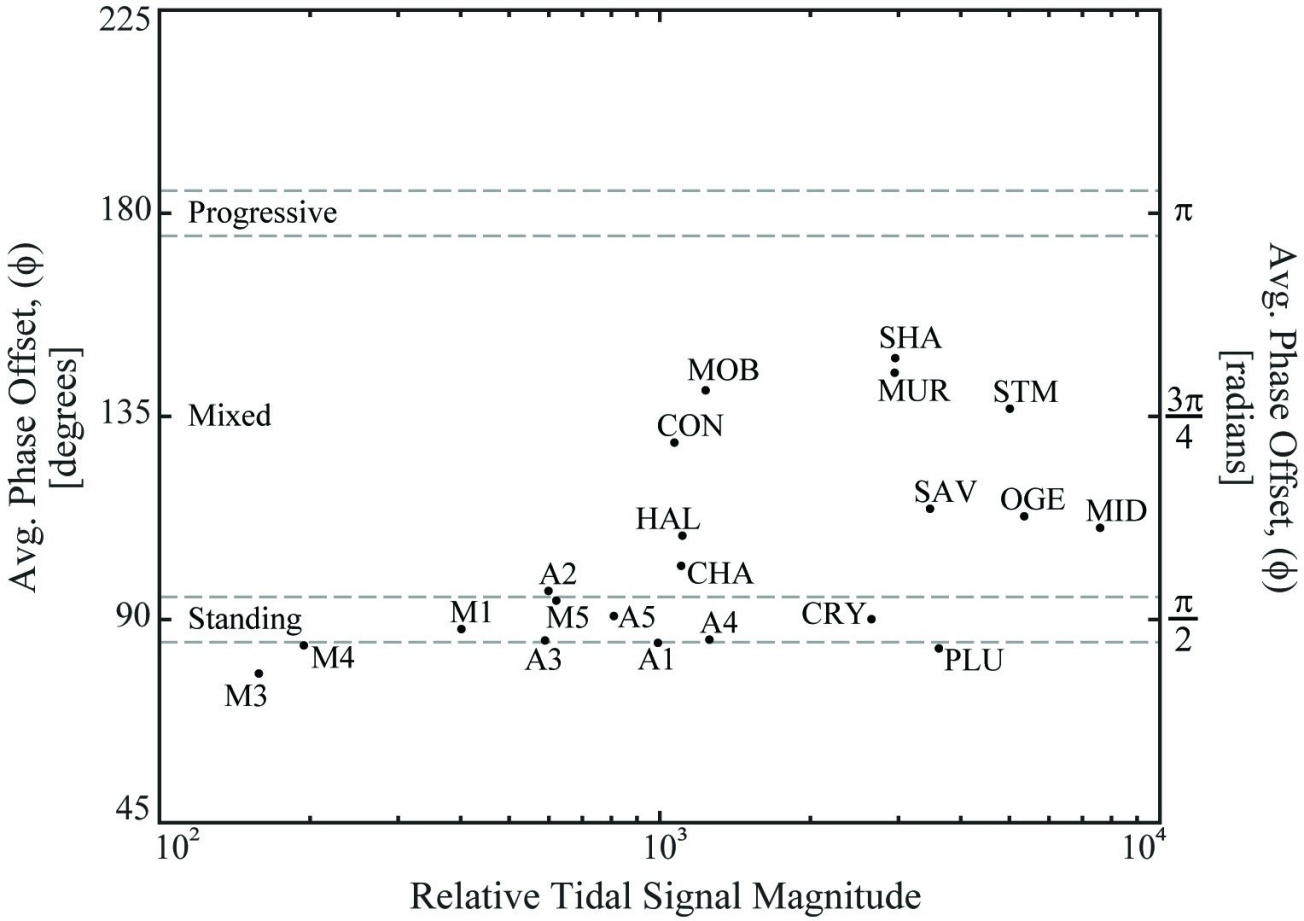
Range of tidal fourier transform magnitudes vs. velocity phase offsets (ϕ) across sites. Site labels are the same as those in Table 1. To enable comparison among sites, the Relative Tidal Signal Magnitude represents the maximum power spectrum magnitude observed during each site’s FFT analysis. Although the variable is unitless, this variable serves as a proxy for the maximum tidal harmonic amplitude (i.e., larger magnitudes ~ larger amplitudes). However, this metric is used for illustration purposes and aides in the graphical differentiation of each site, but the variable does not completely represent the multiple important tidal harmonics at each site. Visualization created using MATLAB software.

The set of 12 USGS validation stations did span a wide range of mixed-to-standing tidal conditions (Fig 3, Table 1) and, as the only available stations meeting the need for USGS-certified discharge and stage data in the tidal reaches of US rivers, the set was adopted as sufficient for the purposes of this study. The 12 USGS stations resulted in two standing and ten mixed wave systems, with phase offsets ranging from ~ $0.93*\frac{\pi }{2}$ (Plum Island River) to ~ $1.64*\frac{\pi }{2}$ (Shark River). None of the tidal USGS stations analyzed exhibited a progressive wave offset of π. Each site’s phase offset and its resulting waveform classification as standing, mixed, or progressive is listed in Table 1 and illustrated in Fig 3. A ±5 degree (±0.087 radian) margin was used around the standing and progressive phase offset definitions (gray dashed lines in Fig 3). The USGS tidal river gauge on the Crystal River of southwestern Florida represented the validation station most similar to standing wave characteristics. None of the validation rivers exhibited true progressive wave characteristics. However, the frictionless channel and infinite length requirements of progressive wave systems make such systems rare in nature [8]. The validation station closest to progressive wave character was the Shark River of southern Florida, followed by the Murderkill River of Delaware.

**Table 1:** List of sites used for validation and testing of the tidal rating curve model.

Site Name	USGS Gauge Number	Label	Mean Discharge ± Std. Deviation [m^3^ s^-1^]	Mean Tidal Range, [m]^++^	Phase Offset ϕ [Rad]	Phase Offset ϕ [deg]	Wave Type
**Tidal USGS Model Validation Sites**							
Connecticut River	01193050	CON	501.00 ± 520.48	0.78	$1.44*\frac{\pi }{2}$	129.20	Mixed
Plum Island River	424752070491701	PLU	2.77 ± 57.36	2.63	$0.93*\frac{\pi }{2}$	83.61	*Mixed
Murderkill River	01484085	MUR	2.62 ± 128.33	1.49	$1.61*\frac{\pi }{2}$	144.68	Mixed
Middle River	02198950	MID	30.10 ± 255.28	2.11	$1.23*\frac{\pi }{2}$	110.31	Mixed
Savannah River	02198980	SAV	479.75 ± 4641.36	2.11	$1.27*\frac{\pi }{2}$	114.55	Mixed
Ogeechee River	02203536	OGE	90.40 ± 253.48	2.11	$1.25*\frac{\pi }{2}$	112.88	Mixed
St. Mary's River	02231254	STM	40.40 ± 559.89	1.83	$1.52*\frac{\pi }{2}$	136.72	Mixed
Chassahowitzka River	02310663	CHA	2.46 ± 21.22	0.86	$1.13*\frac{\pi }{2}$	101.90	Mixed
Halls River	02310689	HAL	0.91 ± 4.02	0.86	$1.21*\frac{\pi }{2}$	108.59	Mixed
Crystal River	02310747	CRY	5.02 ± 129.08	0.86	$1.00*\frac{\pi }{2}$	90.08	Standing
Mobile River	02470629	MOB	654.88 ± 530.81	0.45	$1.56*\frac{\pi }{2}$	140.78	Mixed
Shark River	252230081021300	SHA	9.62 ± 143.20	0.22	$1.64*\frac{\pi }{2}$	147.92	Mixed
**Aransas and Mission River Model Application Sites**							
Aransas River site 1 (most upstream)	n/a	A1	0.27 ± 1.36^**+**^	0.11	$0.94*\frac{\pi }{2}$	84.84	*Mixed
Aransas River site 2	n/a	A2			$1.07*\frac{\pi }{2}$	96.34	Mixed
Aransas River site 3	n/a	A3			$0.95*\frac{\pi }{2}$	85.36	Standing
Aransas River site 4	n/a	A4			$0.95*\frac{\pi }{2}$	85.54	Standing
Aransas River site 5 (most downstream)	n/a	A5			$1.01*\frac{\pi }{2}$	90.77	Standing
Mission River site 1 (most upstream)	n/a	M1	1.15 ± 5.51^**+**^	0.11	$0.98*\frac{\pi }{2}$	87.88	Standing
Mission River site 2	n/a	M2			**	**	**
Mission River site 3	n/a	M3			$0.87*\frac{\pi }{2}$	78.04	*Mixed
Mission River site 4	n/a	M4			$0.94*\frac{\pi }{2}$	84.28	*Mixed
Mission River site 5 (most downstream)	n/a	M5			$1.05*\frac{\pi }{2}$	94.20	Standing

Labels are as in Figs 1 and 3. Mean discharge is from July 2015-July 2017.

^+^ For M-A sites, discharge is reported from each river’s nearest USGS gauging station, which are non-tidal and significantly upstream (Aransas ID: 08189700; Mission ID: 08189500).

^++^ As reported by the nearest NOAA tidal gauge station summary [60]. Section H in the S1 File reports the NOAA gauges were used to determine these values.

* Phase offsets are likely of strongly standing character, even though slightly beyond ± 5 degree margin.

** Insufficient TCM velocity data to accurately determine ϕ.

#### 3.1.2 Baseflow tidal rating curve

To validate the baseflow tidal rating curve model, we first calibrated the model on two weeks of baseflow stage and discharge data from each of the 12 USGS stations (as in section 2.4.1) and then applied the calibrated model using only the stage data from a different two weeks of baseflow at each tidal USGS station. The dates used for calibration and validation at each USGS site are listed in Section I in the S1 File. Calibration regression fit was assessed by the significance (*p* < 0.05) and t-statistic of each of the four model calibration parameters and by the overall adjusted R^2^ of the calibration regression (Table 2). Validation success was evaluated by the R^2^ between the modeled discharge, based only on the measured stage records, and the measured discharge, as measured and reported by the USGS (Table 2).

**Table 2:** Tidal rating curve model coefficients (k_1_, k_2_, k_3_, k_4_, standard errors, and t-statistics) for the validation and test sites under baseflow conditions.

Site	k_1_ ± SE (tStat)	k_2_ ± SE (tStat)	k_3_ ± SE (tStat)	k_4_ ± SE (tStat)	Calibration Adj. R^2^	Validation R^2^
**Tidal USGS Model Validation Sites**						
**CON**	-2408.66 ± 32.63	1784.70 ± 28.36	7.20 ± 0.52	-99.22 ± 1.14	0.82	0.82
	(-73.83)	(62.94)	(13.78)	(-87.32)		
**PLU**	1.69 ± 0.55	2.02 ± 0.61	0.06 ± 0.00	-4.82 ± 0.04	0.92	0.86
	(3.09)	(3.34)	(16.76)	(-129.99)		
**MUR**	-140.95 ± 7.96	53.54 ± 10.78	1.14 ± 0.06	-12.60 ± 0.17	0.79	0.72
	(-17.71)	(4.97)	(18.79)	(-74.53)		
**MID**	62.20 ± 2.48	-99.29 ± 2.64	1.05 ± 0.02	-21.87 ± 0.21	0.90	0.74
	(25.06)	(-37.63)	(63.66)	(-106.60)		
**SAV**	690.80 ± 34.86	-2937.24 ± 28.13	4.82 ± 0.24	-444.94 ± 2.30	0.97	0.96
	(19.82)	(-104.40)	(19.86)	(-193.05)		
**OGE**	-55.68 ± 3.62	0.00	2.79 ± 0.08	-43.15 ± 0.49	0.85	0.69
	(-15.39)		(34.80)	(-88.61)		
**STM**	82.77 ± 11.05	-771.16 ± 5.93	2.87 ± 0.08	-67.99 ± 0.48	0.96	0.89
	(7.49)	(-130.03)	(37.40)	(-142.39)		
**CHA**	-100.89 ± 3.13	57.69 ± 1.72	0.00	-9.30 ± 0.11	0.85	0.81
	(-32.25)	(33.49)		(-88.57)		
**HAL**	-73.56 ± 4.89	13.88 ± 1.31	1.21 ± 0.12	-5.24 ± 0.07	0.85	0.59
	(-15.05)	(10.59)	(10.38)	(-79.84)		
**CRY**	-20.50 ± 0.56	82.46 ± 2.21	-1.62 ± 0.11	-41.17 ± 0.21	0.97	0.96
	(-36.33)	(37.31)	(-14.79)	(-200.16)		
**MOB**	-3740.80 ± 75.28	2079.38 ± 43.20	-9.20 ± 3.36	-113.23 ± 2.84	0.74	0.52
	(-49.69)	(48.13)	(-2.74)	(-39.81)		
**SHA**	-1230.88 ± 36.00	-919.98 ± 16.40	-9.10 ± 0.26	-22.94 ± 0.60	0.85	0.71
	(-34.19)	(-56.09)	(-34.81)	(-38.08)		
**Aransas and Mission River Model Application Sites**						
**A1**	0.67 ± 0.15	-1.06 ± 0.27	0.00	-2.59 ± 0.38	0.44	n/a
	(4.44)	(-4.00)		(-6.73)		
**A2**	0.00	0.39 ± 0.10	-2.22 ± 0.92	-1.11 ± 0.27	0.50	n/a
		(3.96)	(-2.42)	(-4.09)		
**A3**	-3.43 ± 0.37	7.39 ± 0.85	0.00	-8.76 ± 0.32	0.94	n/a
	(-9.20)	(8.73)		(-27.05)		
**A4**	-2.22 ± 0.62	5.63 ± 1.50	4.40 ± 1.60	-10.47 ± 0.63	0.83	n/a
	(-3.6)	(3.74)	(2.75)	(-16.67)		
**A5**	*	*	*	*	*	n/a
**M1**	-0.30 ± 0.03	0.00	0.00	0.00	0.17	n/a
	(-8.90)					
**M2**	**	**	**	**	**	n/a
**M3**	0.63 ± 0.15	-1.01 ± 0.24	-4.35 ± 0.48	0.00	0.69	n/a
	(4.22)	(-4.28)	(-9.10)			
**M4**	0.28 ± 0.05	0.00	0.00	-5.58 ± 0.80	0.81	n/a
	(5.48)			(-7.00)		
**M5**	-6.23 ± 1.29	18.84 ± 3.92	0.00	0.00	0.51	n/a
	(-4.83)	(4.81)				

Coefficient *p*-values were all between 0.00 and 1.85 x 10^−2^. Values of k_i_ = 0.00 indicate terms not included in a site’s final model (see section 2.4.1).

* No ADP transects were collected for calibration at site A5 due to safety concerns.

** Insufficient monitoring data were collected at site M2 for calibration due to instrumentation issues.

### 3.2 Mission and Aransas longitudinal tidal phase analysis

The place of the M-A sites among the continuum of progressive-mixed-standing wave influences was assessed using phase offset analysis, as for the tidal USGS stations. Instead of the reported discharge and stage data used for the USGS stations’ phase analysis, the TCM velocity data and pressure transducer stage data collected at each M-A site were used for M-A site phase analysis. The minimal discharge and tide at the M-A sites resulted in substantial external noise, requiring data cleaning via a moving average longer than 3 hours. A 6-hour moving average removed noise (e.g., wind) from the M-A data before applying the FFT to extract the dominant tidal harmonics exhibited in the stage and velocity time series. As site M2 had insufficient TCM data due to instrument malfunctions, only nine M-A sites could be analyzed. A FFT was applied to the longest continual dataset of stage and TCM velocity available at each site (minimum: 129 days at A2, median: 366 days, maximum: 731 days at A1), which isolated the Fourier phase of the harmonics that contributed the greatest signal power. For both velocity and stage, and at all nine sites, the most prominent harmonics were the local semidiurnal and two diurnal tidal harmonics, as was the case for the USGS validation stations. These harmonics matched the M_2_, O_1_, and K_1_ harmonics published for Copano Bay, which is the downstream receiving water for both the Mission and Aransas Rivers [60].

The M-A sites all exhibited standing wave-like conditions during the period of study, whereas the USGS stations ranged from standing through mixed to nearly progressive waveform types. More specifically, eight of the nine analyzed M-A sites were characterized or nearly characterized by a standing waveform with a phase offset of approximately $\frac{\pi }{2}$ (Fig 3, Table 1). The remaining M-A site, A2, exhibited a mixed wave that was very nearly of standing character. Section B in the S1 File summarizes each M-A site and USGS station’s tidal harmonic and phase offset analysis results.

### 3.3 Calibrated tidal rating curve models for the Mission and Aransas Rivers

Calibration data for the 10 installed M-A monitoring sites were collected by deploying an Acoustic Doppler Profiler (ADP; SonTek RiverSurveyor or Teledyne StreamPro) across river transects at each site at multiple times throughout the two-year study period. To obtain a discharge measurement, the ADP was mounted to a small dinghy and towed slowly across the channel, perpendicular to flow. The downward-facing ADPs used divergent sonar beams to acquire high-resolution, cross-sectional velocity measurements from which to calculate volumetric discharge [23,24,61,62]. To ensure reliable estimates of discharge, we verified that surveys used in analysis contained a sufficiently high signal-to-noise ratio (> 50:1) and did not exhibit spatial errors within the recorded transect [61,62]. In total, 435 ADP usable transects were collected during 2016 and 2017. The collected data were sufficient to calibrate the tidal rating curve model at eight of the ten sites; site A5 had to be omitted from ADP surveying due to on-water safety concerns and site M2 experienced monitoring site instrumentation failures.

**Fig 4:**
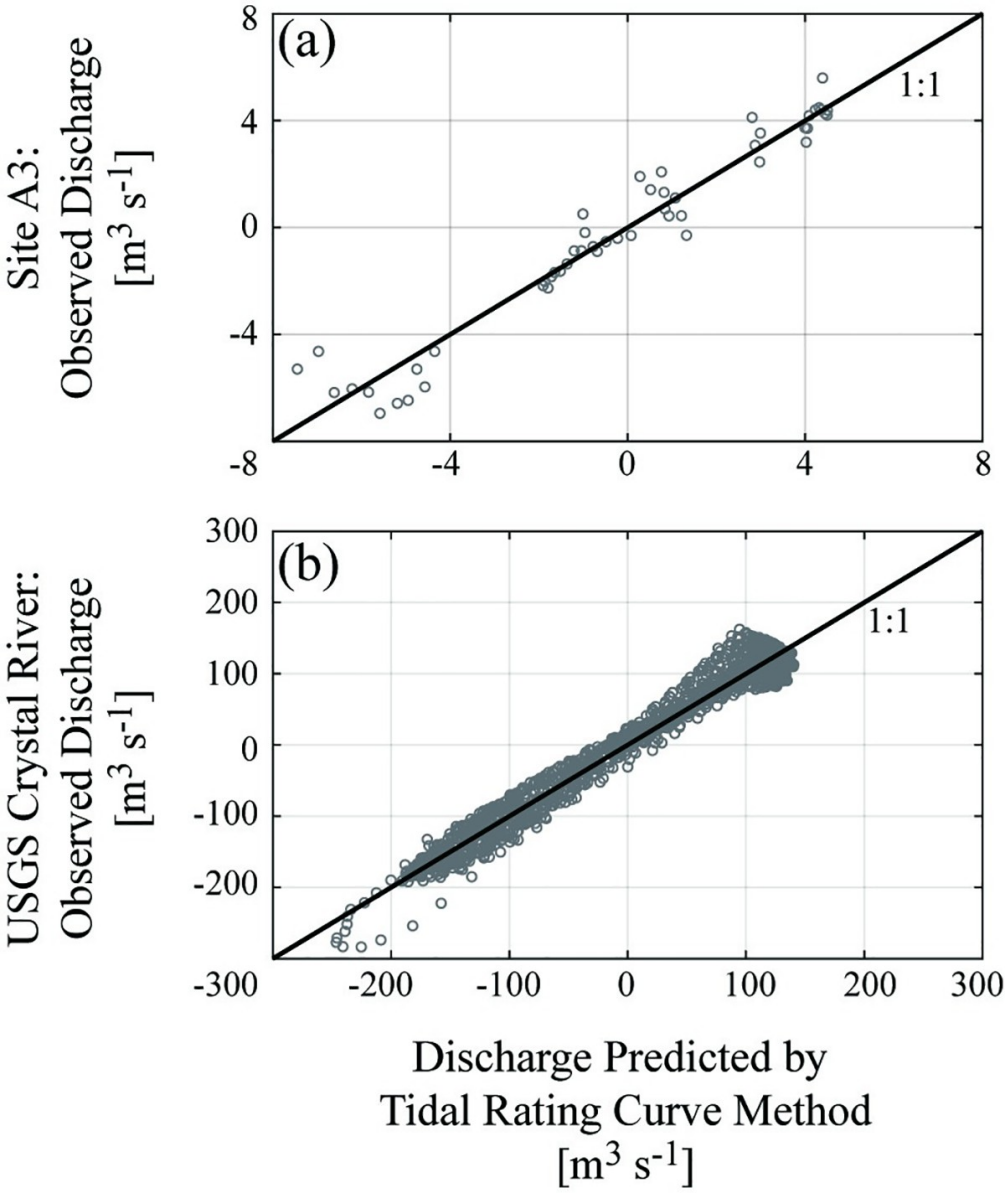
Comparison of observed and modeled discharge at two standing wave sites. Example comparisons of baseflow discharge data from M-A site A3 observed by ADP surveys (a) and at the USGS Crystal River gauge (b) versus discharges modeled by the tidal rating curve method. The solid line represents the 1:1 ratio. Predictions at site A3 exhibited R^2^ = 0.94, while predictions at the USGS Crystal River site displayed R^2^ = 0.97. Visualization created using MATLAB software.

At the 8 M-A sites with sufficient data available, the tidal rating curve model for baseflow conditions was successfully calibrated, producing site-specific and statistically significant calibration coefficients (Table 2). Model terms pruned as per the procedure in section 2.4.1 have values of zero in Table 2. The coefficients exhibited generally small standard errors relative to each coefficient’s magnitude. The M-A sites spanned a broad R^2^ range from 0.17 at site M1 to 0.94 at site A3. The M-A sites with the strongest agreement between modeled and observed discharges were the most downstream sites, A3 (R^2^ = 0.94) and A4 (R^2^ = 0.83). For example, Fig 4 illustrates the good agreements between observed and modeled discharges at Aransas site A3 (Fig 4A) and at the USGS Crystal River (station 02310747, Fig 4B). All non-zero k_4_ coefficients were negative in the calibrated models, inversely relating $\frac{dS}{dt}$ to discharge. Several other sites exhibited an inverse discharge relationship with $(\frac{dS}{dt}{)}^{2}$ (A2, M3, Crystal River, Shark River). All M-A sites except for M1 and M5 included $\frac{dS}{dt}$ as an important explanatory variable (i.e., included k_3_ and/or k_4_) for modeling river discharge through the monitoring locations’ cross-sections. Section C in the S1 File graphically summarizes all sites’ calibrated tidal rating curve models.

### 3.4 Mission and Aransas tidal discharge time series

For the eight M-A sites with calibrated tidal rating curve models we calculated a complete discharge time series over both baseflow and stormflow conditions. Table 3 summarizes each site’s discharge regime during baseflow or stormflow periods, and overall for the full two years from July 2015—July 2017. Section D in the S1 File depicts each M-A site’s discharge time series plot.

**Table 3:** Summary of Mission-Aransas sites’ discharge (m^3^ s^-1^) during baseflow, storm, and overall conditions from July 2015 –July 2017.

Site Name	Discharge Mean ± SD (min; median; max)		
	Baseflow	Storm	Overall
**A1**	0.12 ± 1.09 (-4.03; 0.14; 3.79)	6.76 ± 8.25 (1.57; 3.62; 64.09)	0.79 ± 3.44 (-4.03; 0.31; 64.09)
**A2**	0.21 ± 0.69 (-24.70; 0.46; 0.97)	5.16 ± 4.16 (2.02; 3.58; 23.06)	0.71 ± 2.10 (-24.70; 0.52; 23.06)
**A3**	-1.92 ± 3.56 (-17.64; -2.05; 9.26)	11.42 ± 7.91 (5.41; 8.81; 64.40)	-0.58 ± 5.80 (-17.64; -1.48; 64.40)
**A4**	0.17 ± 3.78 (-9.84; -0.42; 33.92)	15.45 ± 7.51 (8.36; 13.49; 74.15)	1.70 ± 6.29 (-9.84; 0.18; 74.15)
**A5**	*	*	*
**M1**	-0.77 ± 0.18 (-1.54; -0.79; -0.25)	7.70 ± 9.66 (1.40; 3.83; 59.20)	0.07 ± 3.98 (-1.54; -0.76; 59.20)
**M2**	**	**	**
**M3**	-0.30 ± 0.84 (-61.78; -0.16; 1.34)	10.55 ± 14.01 (1.62; 4.75; 84.03)	0.78 ± 5.56 (-61.78; -0.10; 84.03)
**M4**	1.29 ± 1.78 (-16.56; 1.29; 8.16)	17.85 ± 19.43 (3.65; 9.44; 98.68)	2.95 ± 8.09 (-16.56; 1.57; 98.68)
**M5**	0.97 ± 2.75 (-11.72; 1.27; 8.43)	11.57 ± 2.73 (7.82; 10.76; 20.61)	2.03 ± 4.20 (-11.72; 1.68; 20.61)

* No ADP transects were collected at site A5 due to safety concerns.

** Insufficient monitoring data were collected at site M2 due to instrumentation issues.

Overall, between July 2015 –July 2017, average and median discharges modeled for the M-A sites strongly reflected baseflow conditions. However, the majority of freshwater transport occurred during storm events. Average storm discharge was several orders of magnitude larger than mean baseflow at each site. For example, site A4’s average storm discharge was 15.45 m^3^ s^-1^ but mean baseflow was 0.17 m^3^ s^-1^. Median discharge increased downstream during storm conditions (e.g., 3.83 m^3^ s^-1^ at M1 to 10.76 m^3^ s^-1^ at M5). During baseflow, typical (median) discharges of each M-A site described negligible net flow, ranging from -2.05 m^3^ s^-1^ (A3) to 1.29 m^3^ s^-1^ (M4). The standard deviation of baseflow discharge increased downstream (e.g., 0.18 m^3^ s^-1^ at M1 to 2.57 m^3^ s^-1^ at M5).

## 4. Discussion

### 4.1 Comparison of Tidal Discharge Estimation Methods

#### 4.1.1 Tidal rating curve vs. index velocity methods

The index velocity method used presently by the USGS to model tidal discharge is similar to the tidal rating curve method by using ADP surveys to calibrate the discharge relationship based on an index variable. In the former, the index variable is index velocity (point-measured flow velocity), in the latter the index variable is river stage. The baseflow portion of the tidal rating curve (Eq 3) effectively reflects the index velocity method’s quadratic equations used to relate index and mean velocities by combining two quadratic equations relating *Q* to *S* and to $\frac{dS}{dt}$.

Just as with the index velocity method, the tidal rating curve method will most accurately model discharge when the set of occasional ADP surveys broadly captures the range of discharge, stage, and tidal conditions, especially both flood and ebb tides. The index velocity method addresses tidal variability by obtaining 50–120 calibration discharge observations over several 12- to 13-hour periods to capture tidal variability. Seasonal variability is captured through smaller sets of calibration data captured “during periods of hydrologic interest (most often high-flow events)” [24]. Comparable guidelines should be followed when applying the tidal rating curve method, to achieve broad discharge calibration data. If a narrow range of conditions is sampled, a tidal rating curve model can still be developed by calibrating Eq 3 to ADP-measured discharge values, but the resulting model will be low quality. M-A site M1 illustrated this challenge. Poor ADP resolution resulted in few viable surveys. Many M1 surveys recorded negligible discharge (0 m^3^ s^-1^ < | *Q* | < 1 m^3^ s^-1^) and occurred during rising stage despite planning efforts to obtain a wider variety of flow. The resulting regression analysis had insufficient constraints to accurately model site M1’s ADP discharge based on stage data (R^2^ = 0.17, Table 2). Still, as a test application of the new method, this is a useful illustration. Section E in the S1 File discusses this issue further.

The types and costs of necessary instrumentation differ between the index velocity and tidal rating curve methods. The index velocity method requires permanent installation of a moderately expensive ADVM or UVM, while the tidal rating curve method requires a much less expensive pressure transducer and tilt current meter. Both methods additionally require a set of ADP discharge surveys that span a variety of flow and forcing conditions. Both methods also presume minimal changes in channel geometry over time, or else new stage-area relations should be developed and the discharge models recalibrated. Due to the methods’ similarities, the tidal rating curve could be employed usefully in a variety of locations world-wide. Applications of the tidal rating curve method may aid research, freshwater or estuarine water quality or ecological management, validation of basin water rights allocations and withdrawals, or integration of coastal watershed flows. More generally, the tidal rating curve would be useful where a time series of tidal riverine discharge is desired but ADV or UVM instrumentation would be too costly to obtain or replace in a hazard- or vandalism-prone area.

#### 4.1.2 Tilt-current meters vs. index or rating curve methods

Ideally, an installed TCM could inexpensively substitute for an ADVM or UVM and measure index velocity for modelling tidally oscillating river discharge. The typical design of a tilt-current meter is an accelerometer attached atop a rod-shaped housing that acts as a lever-arm and enables the accelerometer end of the housing to remain buoyant. With the other end of the housing affixed to the bed, the TCM tilts according to the net force exerted by the current. The resulting angle and direction of deflection from vertical relate to the magnitude and direction of flow [55,56]. TCMs are useful tools, especially for tracking flow direction and magnitude in coastal water bodies [57–59].

**Fig 5:**
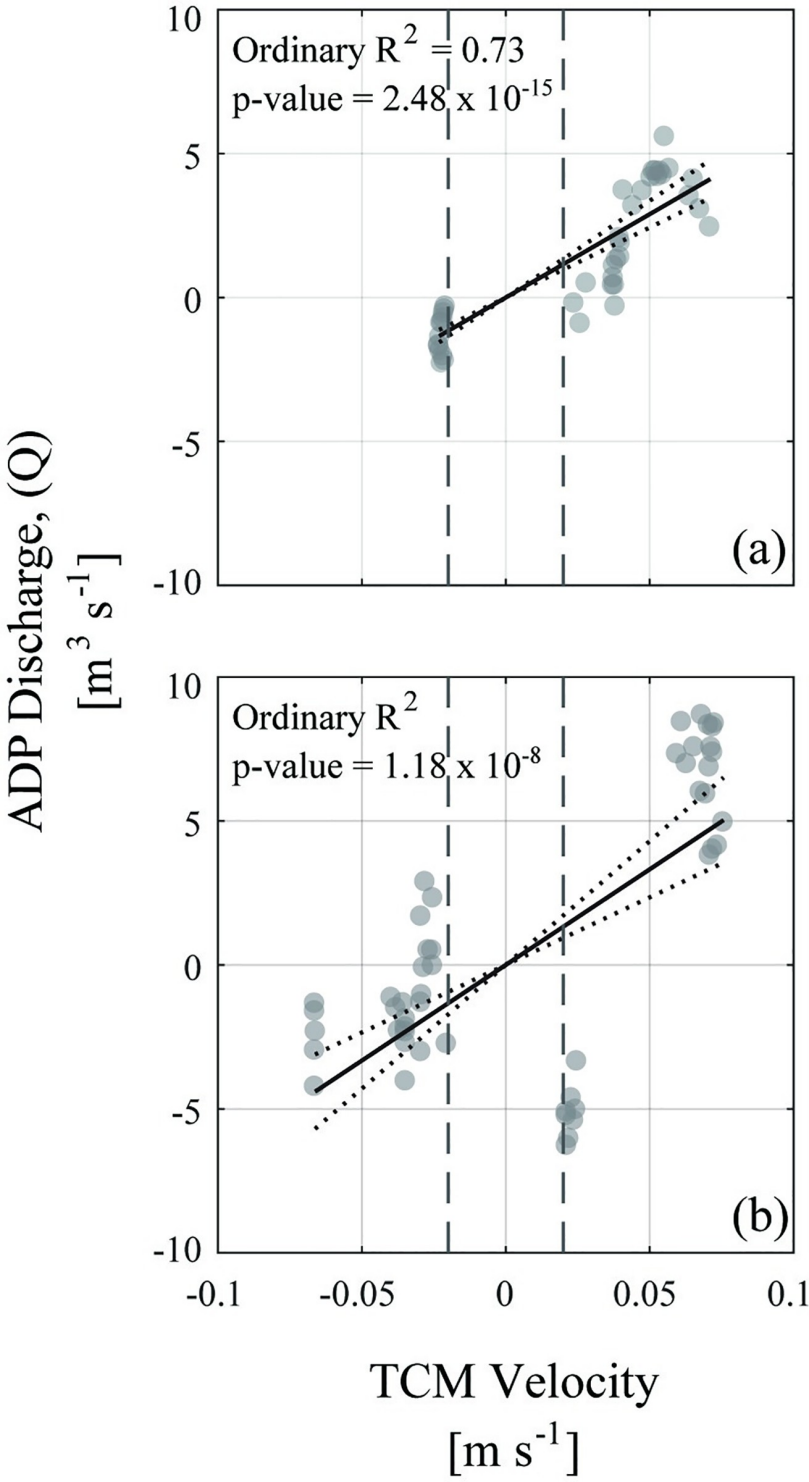
Example regressions of tilt current meter velocities to ADP-measured discharges. Example linear regressions of tilt current meter (TCM) velocities to ADP-measured discharge at Aransas river sites A3 (a) and A4 (b). Gray dashed lines indicate zone of TCM velocity magnitudes less than the 0.02 m s^-1^ reliable detection limit. In each figure panel, circles marking empirical observations are transparent; thus, darker circles denote overlap of observations. Dashed lines represent 95% confidence bounds of the regression. Visualization created using MATLAB software.

This study provided the first such application of TCM instrumentation to monitor river velocity, using the M-A system as a test case. Results did show monotonic relationships between the apparent velocity measured by the TCM and the discharge measured by the ADP at M-A sites A3 and A4 (Fig 5). Notably, these were the most downstream M-A sites, where flow velocities were sufficiently large to be in excess of the sensitivity of the TCM instrument (± 0.02 m s^-1^ in this study) [55,56]. Users deploying TCMs in low-flow riverine environments must be aware of the lower limit of reliable flow detection of the accelerometer and lever arm used. Section F in the S1 File further compares TCM velocities and ADP volumetric discharge data. Although such a limitation naturally also exists for ADVM and UVM instruments, those instruments’ lower threshold for a minimum detectable velocity (e.g., 10^−4^ m/s for Sontek ADV) enables a broader range of use [63]. Still, the use of TCM data in an index velocity method-like approach may be successful in high-flow rivers or locations with fast tidal flows (i.e., regularly exceeding the minimum threshold).

However, using stage data as the sole quantitative inputs to the tidal rating curve model successfully estimated discharge across all M-A sites with sufficient calibration data. Thus, the tidal rating curve provided realistic estimates at sites where observed velocities were often below the TCM sensitivity (e.g., A1). In this study, TCM data are used for three primary purposes: (1) to distinguish stormflow and baseflow time periods, (2) to estimate stormflows, and (3) to monitor changes in upstream- versus downstream-oriented discharge when determining phase offset. TCM observations were not used in baseflow discharge estimation.

### 4.2 Tidal rating curve model considerations

The tidal rating curve method provides an estimate of vertically and horizontally averaged discharge at a tidally-influenced river cross-section. After calibration, this new method produced baseflow discharge results comparable to those of the 12 selected USGS tidal stations (R^2^ of 0.62 to 0.97). The new method also generated reasonable results for the M-A sites’ discharge regimes given our knowledge of baseflow for the semiarid-subtropical M-A physiographic setting.

The tidal rating curve method’s lowest accuracy was exhibited during transitions between baseflow and stormflow. Large stormflow events impacted the surface water stage before significantly altering the measured TCM velocity. Because our criterion for distinguishing stormflow was the 90^th^ percentile of a site’s measured TCM velocities for the period of the study, the lag in TCM velocity response led to baseflow discharge calculations (Eq 3) being applied to the initial portion of some storm hydrographs. Furthermore, rapid stage rise (i.e., positive $\frac{dS}{dt}$) from the hydrograph’s rising limb caused Eq 3 to estimate upstream flow (i.e., negative discharge). However, as a first approximation, times of negative discharge not associated with rising tide may be easily filtered out, improving the overall discharge timeseries with little effort. The use of a single criterion to distinguish storm and baseflow in this study was consistent with the effort to develop the simplest useful model for tidal river discharge requiring the least and lowest cost data requirements. In reality, the transitions between storm and baseflow periods are more complex than the simple binary division used here. Future methodological refinements might establish a more nuanced approach to distinguishing the storm and non-storm regimes.

The presented method models stormflow discharge as an area-scaled TCM velocity (Eq 4), which may be overly simplified and would benefit from more complexity. However, this simple approach reflects both the index velocity method and classic river rating curve method (Section 2.4.2). Using TCM velocities also maintains the parsimonious approach desired in this study, and is a safe and effective means of estimating high flows. For example, the flashy southern Texas hydrologic setting made obtaining ADP surveys unsafe during fast and extreme stormflow conditions. In such environments, any method relying on calibration by ADP data would be highly challenging. This study also assumed that the velocity scaling factor in Eq 4 was *a* = 1, i.e., TCM velocity equals specific discharge. This assumption could be calibrated in future implementations. To best emulate this assumption, we located TCMs in the channel thalweg where accelerometers would roughly observe primary channel flow. However, when high flows angled the TCM arm toward the bed, the TCM accelerometer potentially dropped below the zone of mean velocity where likely *a* ≠ 1. High flows may also result in floodplain inundation that could alter the cross-sectional geometry and representativeness of a TCM measurement. Future implementations can use other existing methods of relating area and stage [23,24] if assuming a rectangular or other regular channel cross-section is deemed unsuitable. If ADP data were available from stormflows, the discharge model for stormflow may be more nuanced (e.g., a calibrated constant of proportionality between TCM velocity and discharge, or additional nonlinear terms). This expansion to the method is also recommended for future implementations, since it could not be implemented and tested while maintaining personnel safety in this study.

Model Eq 3 seeks to describe discharge regimes of mixed wave systems by combining impacts of endmember conditions while leveraging empirical observations, without inducing a mathematical fitting exercise. Eq 3 contains two halves describing progressive and standing waveforms. The k_1_ and k_2_ terms correspond to the progressive wave portion of Eq 3, while k_3_ and k_4_ terms relate to standing waves. Thus, each modeled coefficient should reflect the expected waveform properties and relationships between tidal discharge, stage, and $\frac{dS}{dt}$ identified by the phase offset analysis. In addition, section 2.4.1 discusses how each of the modeled coefficients might relate to channel geometry.

Both halves of the equation are characterized as second-order polynomials to mimic the power law relationship relating non-tidal riverine stage to discharge employed by the USGS [3].

$$Q_{i}=a*{\left(S_{i}-b\right)}^{c}$$ (5)

In Eq 5, Q_i_ and S_i_ are the discharge and stage at time, t_i_, the value of *b* represents the stage measurement for which no discharge occurs, and the value of *a* is a fit coefficient.

However, a power law exponent, *c*, cannot be determined empirically or analytically for a variable that contains negative values such as $\frac{dS}{dt}$. For an empirical derivation, data is plotted in log-log space and the slope of the resulting linear trend characterizes the exponent [3]. However, negative $\frac{dS}{dt}$ values cannot be plotted in Real log-log. Similarly, for an analytical solution, Eq 5 is deconstructed using logarithms that result in complex imaginary solutions for negative $\frac{dS}{dt}$.

**Fig 6:**
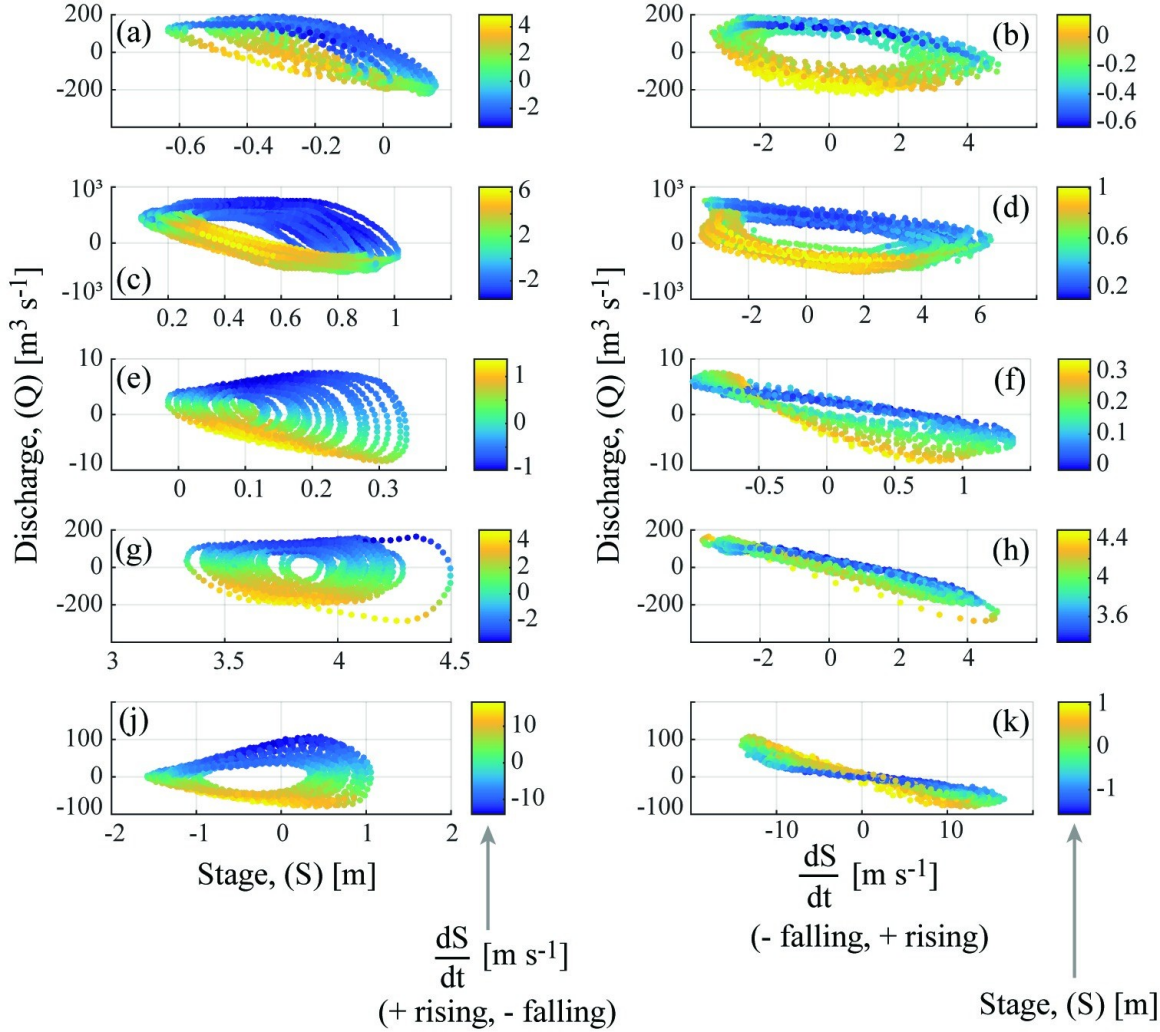
Elliptical relationships between stage, stage-rate-of-change, and discharge. Discharge (*Q*) relationships to stage (*S*, left column) or stage-rate-of-change ($\frac{dS}{dt}$, right column) at five of the tidal USGS sites during two weeks of baseflow, with points colored by the other variable ($\frac{dS}{dt}$, left; *S*, right). An ideal progressive-wave system would exhibit linear *Q*(*S*) and an ideal standing-wave system would exhibit linear *Q* ($\frac{dS}{dt}$). Sites are: (a-b) Shark River, (c-d) Connecticut River, (e-f) Halls River, (g-h) Crystal River, (j-k) Plum Island River (see Table 1). The Shark, Halls, and Plum Island Rivers measured stage relative to a zero MSL datum, hence positive and negative stage; the Connecticut and Crystal Rivers used a lower datum. Data for these rivers was obtained from USGS NWIS [50] and visualized via MATLAB.

Instead, we used two second-order polynomials to address the immiscibility of logarithms and negatives, while attempting to describe the elliptical relationship between tidal discharge, stage, and $\frac{dS}{dt}$ (Fig 6). Linear regressions of stage and $\frac{dS}{dt}$ would be insufficient to accurately estimate tidal discharge. Studies along the Sacramento River estimated tidal river discharges using a power law (on a “tidal property ratio”) whose exponent, γ, ranged between $\frac{2}{3}$ ≤ γ ≤ 2 [12]. At the upper extreme, this power law would mimic a second-order polynomial.

Although the second-order polynomial assumes the simplest complexity after a linear characterization, there is an issue with using the second-order polynomial. The symmetrical character of second-order polynomials pose a potential problem as conditions outside those observed, where sharply rising stage (e.g., $\frac{dS}{dt}$ > 2) may produce erroneous discharge estimates.

To exhibit the tidal rating curve’s applicability across different climate zones and river systems, we applied the tidal rating curve to twelve different tidal USGS gauges (Table 1, Fig 2A). While the tidal rating curve displayed strong calibration and validation R^2^ for twelve USGS sites (Table 2), the twelve USGS sites only covered the range of climatic conditions experienced across the eastern coastline of the United States. Future iterations of the tidal rating curve need to be refined through application to different river systems and climatic regimes. For example, applying this method to the Columbia River (northwestern USA) would improve this method’s usefulness across large river systems and new climate zones. Other future iterations of the tidal rating curve should also look outside the United States for potential field sites that, again, would provide unique riverine and climatic dynamics.

### 4.3 Longitudinal variations in Tidal River discharge

Theoretically, for non-tidal rivers, increasingly large watershed areas contribute to streamflow at sites farther downstream, which results in discharge increasing downstream [1]. In the M-A rivers, only stormflow discharge increased downstream. During baseflow, the mean and median discharges calculated at each M-A site over the two-year study period were not significantly different from zero in the Aransas River (A1-A5) or at the three downstream Mission River sites (M3-M5). Yet, the standard deviation (SD) of baseflow discharge increased downstream in both rivers (Table 3). This is consistent with the expectation that a non-tidal river’s baseflow should remain fairly consistent (i.e., low SD further inland) and oriented downstream, but a tidal river should exhibit more variable discharge (i.e., larger SD toward mouth) [62]. However, approximately zero average baseflow and increasing discharge SD downstream suggest that tidal energy effectively opposes baseflow discharge. This occurs in the Aransas River up to at least the geographic position of A1, and in the Mission River up to between sites M3 and M1. Another study identified a tidal river reach with zero net mean discharge, averaged over a week in June 2008, by comparing ADP observations between ebb and flood tide [64]. In their study, net mean discharge at upstream sites was oriented downstream, while downstream sites recorded net mean discharge oriented upstream with nearly equal magnitude [64]. Granted, although this study’s limited temporal observations may not be universally applicable, the implication of these findings is that fluid, solute, sediment, and aquatic organism kinematic residence times may be very long in these tidal riverine environments. Although a reasonable hypothesis *a priori*, this implication is difficult to validate as longitudinal variations in discharge are not often monitored and recorded in lowland tidal rivers, nor in riverine tidal freshwater zones [16]. Whether modeled or measured, longitudinal observations in estuaries typically focus on saline intrusion and dispersion [8,65], not longitudinal variations in upstream freshwater discharges. Likewise, studies of fresh river flow very seldom extend into the tidal zone. The impacts of these very long riverine tidal freshwater zone (TFZ) residence times are likely significant and understudied, with nutrients potentially residing in the riverine TFZ for extended periods (e.g., months) until a storm flushes the system [16,18,21].

However, estimated net-zero discharge does not indicate that no freshwater was transported through the tidal river reach. From the conservation of mass, water input to the tidal freshwater zone must either be stored or removed (e.g., discharged, evaporated, or intruded into groundwater). Thus, assuming negligible changes in storage, the observed tidal freshwater zone outflow must approximately equal inflow when averaged over long periods of baseflow. While the majority of surface export of freshwater occurred during storm flows, the estimated net-zero discharge and minimal input from the M-A rivers suggests that more work is necessary to adequately resolve the fate of these outflows.

For the M-A sites, baseflow discharges are minimal since the hydrologic climate is dominated by evaporation. As such, we assumed that freshwater baseflow did not significantly impact tidal discharge estimates. However, for larger river systems with significant baseflow, riverine freshwater discharges may influence tidal discharge estimates. Future iterations of the expanded tidal rating curve model should work to incorporate the impact of freshwater discharge on empirical tidal discharge estimates during baseflow conditions. For the M-A sites, the primary impacts of freshwater discharge were observed during stormflow events and, thus, are accounted for in that portion of the piecewise model.

In contrast to the very low baseflow discharge of the M-A rivers, stormflows were significant. The majority of M-A freshwater export, as determined using the piecewise tidal rating curve model, occurred during storm events, which concurs with previous M-A studies [16,18,21]. During baseflow, all M-A sites’ average and median downstream discharges did not exceed 1.5 m^3^ s^-1^. However, the discharges from stormflow periods during the two-year study were substantially larger, with maximum storm discharges exceeding typical baseflow by nearly three orders of magnitude. For example, site M3 exhibited a mean baseflow of -0.30 m^3^ s^-1^, mean storm flow of 10.55 m^3^ s^-1^, and maximum storm flow of 84.03 m^3^ s^-1^ (Table 3). The disparity in discharge volume between baseflow and storm flow reflects the climate of south Texas and its impact on the M-A Rivers. During the study period, 01 July 2015 to 01 July 2017, NOAA reported an El Niño Southern Oscillation (ENSO) event, which brings cooler temperatures and increased precipitation frequency to south Texas [48,66,67]. However, even without ENSO effects, south Texas experiences minimal baseflow that is punctuated by convective and tropical storm runoff that promotes flooding, which would produce a similar discharge summary [14,16,18,21,45]. For the M-A systems, the tidal rating curve method improved and quantified our understanding of baseflow v. stormflow both in their relative importance over time and their relative magnitudes.

### 4.4 Discharge consequences of Tidal River waveforms

The phase offset analysis determined each USGS and M-A site’s tidal waveform, whether standing, mixed, or progressive (Table 1). Among the 20 sites analyzed, standing and mixed conditions were represented but the progressive case was not observed. A purely progressive waveform requires a frictionless channel of constant cross-section and infinite length, which may be rare in the natural world [8]. The Crystal River stage-discharge phase offset most nearly matched the perfect theoretical standing wave (ϕ = ~ $\frac{\pi }{2}$ or ~ 90°, Table 1) and so, as expected, Crystal River discharge was highly related to $\frac{dS}{dt}$ (Fig 6H, linear regression R^2^ = 0.94). In contrast, the Shark River, which was the closest to being a progressive wave among the study sites, exhibited discharge more related to *S* (Fig 6A, linear regression R^2^ = 0.39). Since mixed waves naturally combine progressive and standing wave characteristics, we should expect mixed-system tidal rating curve models (i.e., Eq 3) to include relationships between discharge and both $\frac{dS}{dt}$ and stage. In fact, tidally-influenced *Q/S* relationships are more or less elliptical (Fig 6A, 6C and 6E). This non-monotonic relationship is the primary factor that inhibits the accuracy and use of traditional rating curve methods, which relate discharge to stage [24,68]. This was supported by the results from the mixed-waveform systems in the study, each of which included both a negative stage to discharge relationship (i.e., negative k_1_ and/or k_2_) and a negative $\frac{dS}{dt}$ to discharge relationship (i.e., negative k_3_ and/or k_4_, Table 2). Fig 6 more generally illustrates tidal discharge’s dual dependence on stage and $\frac{dS}{dt}$ for mixed-waveform settings, as scatter plots of five USGS stations’ data.

In addition to the insights it provides for predicting tidal river discharge, as a future application, this stage-discharge phase offset analysis may provide a method for early forecasting of rising sea level’s impact on estuarine systems. As sea level continues to rise, estuarine geometries will change and potentially result in different waveforms transporting tidal energy upstream [8]. An observed temporal shift in waveform character may indicate a critical threshold for sea level change. Altered waveform transport may induce significant cascading ramifications for the transport and timing of nutrients and freshwater discharging to the coastal environment. Observing such a phase shift might forecast potential dramatic alterations in associated riverine, estuarine, and coastal ecosystems that might lag in time.

## 5. Summary and conclusion

This study expanded the classic rating curve often used in gauging non-tidal rivers to a similarly simple model for tidal rivers during baseflow. The classic method models discharge using an empirically calibrated regression with stage. The tidal rating curve method maintains consistency with the classic model structure while also incorporating the time rate of change of stage into the regression model. The representation of discharge during baseflow conditions as a combined function of stage and stage-rate-of-change was shown to adequately represent tidal river discharge across the continuum of progressive-mixed-standing waveform systems, as validated using all the available tidal USGS gauges with sufficient data during the study period (R^2^ ≥ 0.5). The validation demonstrated the value of the parsimonious tidal rating curve model to estimate tidally-influenced river discharge comparably to, but at lower cost compared to, the index velocity method that is presently used by the USGS for discharge in tidal settings [23,24]. The characterization of each tidal site as of standing, mixed, or progressive wave character was accomplished by FFT analyses on sites’ stage and velocity time series. TCMs provided velocity time series observations for this phase offset analysis. To also capture stormflow conditions, a piecewise modeling approach was adopted, simulating baseflow using the tidal rating curve model and stormflow as area-scaled specific discharge. This piecewise tidal rating curve method was applied *de novo* to calculate discharge time series at eight sites longitudinally distributed in the riverine tidal freshwater zones (TFZs) of the Mission and Aransas Rivers of south Texas. The resultant discharge time series for the Mission and Aransas rivers reflected the natural discharge variability of very low baseflows occasionally punctuated by extreme stormflows [16,18,21]. The tidal rating curve method for estimating tidally-influenced baseflow river discharge was shown to provide a new, simple, and inexpensive alternative to existing approaches, requiring only continuous stage data and minimal ADP calibration measurements. TCM data identified stormflow versus baseflow, predicted stormflow discharges, and provided velocity measurements to determine phase offset.

The implications of being able to generate continuous baseflow estimates at low cost and with very simple calibration methods are diverse. Operationally, this approach may encourage researchers, water quality monitors, aquatic ecosystem managers, or auditors of upstream basin withdrawals to begin to monitor river discharge more routinely at the lowest point of the basin, near the river mouth. Expanding the ability to monitor near-shore river flows even to communities with minimal funding could facilitate enhanced understanding of global streamflows in the abundant small coastal watersheds. In addition to basic understanding of land-sea interactions, more publicly available tidal discharge data has the potential to improve efficiency and efficacy of environmental management policies. The stage-discharge phase offset analysis also developed in this work may have potential to enable early detection of the impacts of rising sea level on estuary and lowland river systems, as the nature of waveforms transporting energy upstream through riverine tidal zones may be altered in advance of other more notable consequences occurring. Increased coastal monitoring and publicly available data may also improve environmental education, engagement, and outreach, which may ultimately lead to an improved socio-environmental relationship.

## Supporting information

S1 File Supporting Information for Gauging Tidal Rivers by Expansion of Classic Rating Curve Methods. The supporting information discusses the following topics: (A) methods converting raw tilt current meter (TCM) vector coordinates into velocity data, (B) Fast Fourier Transform (FFT) results indicating primary diurnal and semidiurnal harmonics of each site (M-A and USGS), (C) summaries of the tidal baseflow regressions for each tidal monitoring site (both M-A and USGS), includes the results of Matlab regression output object for each site, (D) discharge time series for each M-A site, including both storm and inter-storm flows, (E) issues and considerations for using TCMs in low-flow environments, (F) assessing the standing waveform classification of the M-A sites, (G) the North American ecological region [69] and Köppen-Geiger climate type [70] zone for each USGS validation site, (H) mean tidal ranges for the nearest tidal gauging sites for each USGS site, (I) calibration and validation dates used at each USGS site, and (J) additional MATLAB files of the model.
(DOCX)
