# Supplementary material for: An expanded rating curve model to estimate river discharge during tidal influences across the progressive-mixed-standing wave systems
Source: PLoS One. 2019 Dec 18;14(12):e0225758. doi: 10.1371/journal.pone.0225758 (PMC6919628; doi:10.1371/journal.pone.0225758)
Supplement: S1 File — The supporting information discusses the following topics: (A) methods converting raw tilt current meter (TCM) vector coordinates into velocity data, (B) Fast Fourier Transform (FFT) results indicating primary diurnal and semidiurnal harmonics of each site (M-A and USGS), (C) summaries of the tidal baseflow regressions for each tidal monitoring site (both M-A and USGS), includes the results of Matlab regression output object for each site, (D) discharge time series for each M-A site, including both storm and inter-storm flows, (E) issues and considerations for using TCMs in low-flow environments, (F) assessing the standing waveform classification of the M-A sites, (G) the North American ecological region [69] and Köppen-Geiger climate type [70] zone for each USGS validation site, (H) mean tidal ranges for the nearest tidal gauging sites for each USGS site, (I) calibration and validation dates used at each USGS site, and (J) additional MATLAB files of the model. (DOCX) [file pone.0225758.s001.docx]

Supporting Information for

**Gauging Tidal Rivers by Expansion of Classic Rating Curve Methods**

Allan E. Jones^1^*, Amber K. Hardison^2^, Ben R. Hodges^3^, James W. McClelland^2^, and
 Kevan B. Moffett^4^

1 – Department of Geological Sciences, University of Texas at Austin, TX, USA

2 – University of Texas Marine Science Institute, Port Aransas, TX, USA

3 – Department of Civil, Architectural and Environmental Engineering, University of Texas at Austin, TX, USA

4 – School of the Environment, Washington State University Vancouver, WA, USA

Corresponding Author: Allan E. Jones

Email: [allan.e.jones@utexas.edu](mailto:allan.e.jones@utexas.edu)

# Contents of this file:

Supplement A (p. 2):
Details of methods converting raw tilt current meter (TCM) vector coordinates into velocity data.

Supplement B (p. 28):
Fast Fourier Transform (FFT) results indicating primary diurnal and semidiurnal harmonics of each site (M-A and USGS).

Supplement C (p. 39):
Graphical summaries of the tidal baseflow regressions for each tidal monitoring site (both M-A and USGS). Each figure is the result of plotting the Matlab regression output object for each site.

Supplement D (p. 50):
Figures of the discharge time series for each M-A site. These discharge time series include both storm and inter-storm flows.

Supplement E (p. 55):
Greater detail on issues and considerations for using TCMs in low-flow environments.

Supplement F (p. 57):
Discussion of assessing the standing waveform classification of the M-A sites.

Supplement G (p. 59):
Provides a table of the ecological and climatic zones of each USGS validation site.

Supplement H (p. 62):
Provides a table of mean tidal ranges for the nearest tidal gauging sites for each USGS site.

Supplement I (p. 63):
Provides a table of the calibration and validation dates used at each USGS site.

Supplement J (p.64):
Provides additional MATLAB files of the model.

# A. Conversion of TCM coordinates to velocity

Raw TCM accelerometer data are recorded as X, Y, and Z coordinates. Calibrating and normalizing the raw data are the first steps in translating from raw X, Y, Z coordinates into local velocity measurements. Calibration tests for each TCM provide the maximum observable X, Y, and Z coordinates, as well as the X, Y, and Z coordinates the accelerometer observes during zero velocity conditions. Throughout each of the 2-3 minute long calibration tests, the TCMs should record observations at a sampling rate ≥ 10 Hz [Sheremet, 2013; Sheremet et al., 2009]. The maximum X, Y, and Z coordinates are determined through *roll* and *swing* calibration tests. The *roll* test identifies the maximum X and Y coordinates while the TCM is rolled slowly along a flat surface. In the *swing* test, a tether attaches the TCM to a vertical wall, and the TCM, remaining flat against the wall, is swung in a circular arc about the tether. The *swing* test identifies the maximum Z coordinate. Each of these tests result in sinusoids of the respective coordinates. The maximum coordinates are calculated as an average of the magnitudes recorded during the crest/trough for each coordinate sinusoid [Sheremet, 2013; Sheremet et al., 2009]. The TCM-specific X, Y, and Z zero-velocity coordinates are empirically derived from average laboratory observations of each TCM submerged in a bucket of water [Sheremet, 2013; Sheremet et al., 2009].

Using a TCM’s maximum and zero-velocity endmembers, that TCM’s field observations may be normalized within this expected range. Thus, post normalization, ideally, zero velocity conditions register as the coordinate vector [0, 0, 1] (i.e., the vertically buoyant TCM is motionless above the X-Y plane’s origin), and maximum discharge conditions, where the TCM would be pointed horizontally in the direction of flow, would register as a coordinate vector of [x, y, 0], where $x^{2}+y^{2}=1$.

However, the submerged field installations of the TCMs introduce errors, e.g., spatial offsets from the true thalweg or rotation of the TCM. These may cause the observed coordinate axes to be out of alignment with the ideal axes corresponding to the primary flow direction (Figure A.1a). This axial offset can be corrected using pitch, roll, and yaw corrections similar to those used in eddy flux observations (e.g., Heinesch, [2006]; Wilczak et al., [2001]; Yuan et al., [2007]). The pitch and roll corrections align the zero velocity conditions of the empirical observations (blue dots in Figure A.1a) to the ideal X-Y origin (black dots in Figure A.1a). The yaw correction rotates the data around the ideal origin and aligns the data with the axis denoting the primary flow direction (i.e., positive y-axis relates to downstream flow, and the negative y-axis denotes upstream flow). To determine the proper flow direction from the field observations, we isolated data associated with large storm events, as these flood discharges would overwhelm the tidal signal and provide a clear indication of the downstream direction. The tilt data associated with storm discharges represented the least common 5% of the data. A k-means cluster analysis (where k=2) partitioned this 5% of the data into two primary groups: one closer and one farther from the origin (yellow and cyan points, respectively, in Figure A.1b). From these clusters, the downstream direction (red arrow in Figure A.1b) was identified as in line with the cluster centroid farthest from the origin (cyan centroid marked with black ‘X’ in Figure A.1b). The yaw correction rotated this downstream orientation to align with the y-positive axis (Figure A.1c). From the pitch, roll, and yaw corrected data (Figure A.1c), we determined the angle of each coordinate vector from the vertical, which corresponded to a laboratory-derived relationship between angle-from-vertical and velocity (Figure A.1d) [Sheremet, 2013; Sheremet et al., 2009]. From this relationship, we obtained a velocity time series for each TCM installation (Figure A.1e).

The resulting TCM velocity time series is accurate down to a magnitude of approximately 2 cm s^-1^ [Sheremet, 2013; Sheremet et al., 2009]. Although the exact accuracy of velocities with magnitude less than 2 cm s^-1^ is uncertain, the timing of tidal oscillations in discharge is sufficiently accurate to provide insight into the impact of diurnal and semidiurnal tidal cycles on the river reach’s discharge regime [Aretxabaleta et al., 2014; Maio et al., 2016].


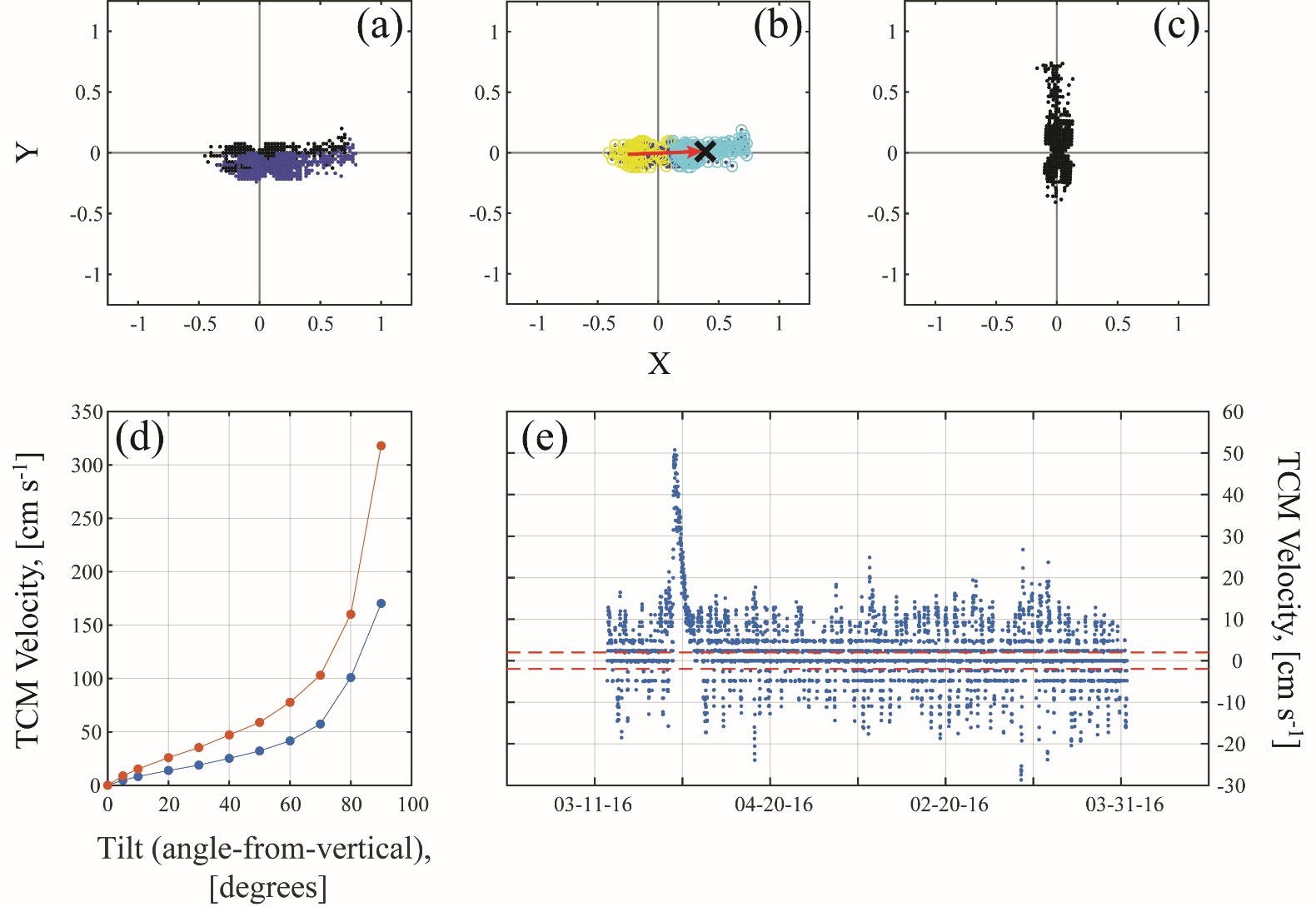


**Figure A.1:** Pitch, roll, and yaw corrections for TCM data and resulting corrected velocity time series. The data presented were observed at site A4 between 02 March and 30 April 2016. The pitch, roll, and yaw corrections align the potentially offset or rotated raw data with the proper downstream flow direction, which we oriented along the positive y-axis. The pitch and roll corrections align the observed, normalized TCM coordinate data (blue dots in (a)) to the ideal X-Y origin (black dots in (a)). The isolated storm data (cyan and yellow data in (b)) are used to determine the primary direction of downstream flow (red arrow in (b)). This downstream trend is yaw-rotated to align with the positive y-axis (c). From the cleaned, normalized, and properly oriented TCM coordinate observations (c), an empirical relationship between tilt (angle-from-vertical) and local velocity (d) create a final velocity time series (e). In (d), the orange and blue lines refer to the tilt-velocity relationship for the ‘p50’ and ‘b15’ SeaHorse TCMs, respectively. The red dashed lines in (e) outline the TCM detection limit of ± 2 cm s^-1^.

## A.1 Outline of TCM calibration, normalization, and rotation

The following is a set of bulleted notes describing the TCM calibration and rotations in greater detail. The Matlab code for the rotations follows that.

- Remove the data hour before and 2 hours after installation of the meters
- Site/TCM specific configuration files provide the normalization coefficients and zero velocity offsets
  - The minimum X and Y normalization coefficients are experienced during the opposing axes maximum
  - The Z normalization coefficients are determined in relation to the X and Y coefficients
- The raw data from the TCM is presented in a left-hand coordinate system, which needs to be translated into a right-handed coordinate system
- The newly translated data is then normalized using the TCM specific configuration coefficients
- We perform pitch, roll, and yaw rotations about the Y, X, and Z axes respectively to align our data with the downstream flow direction of the river channel
  - The pitch and roll rotations center the data on the zero velocity offsets from the TCM-specific configuration files
    - Any lateral or longitudinal offset from the zero velocity coordinates denote a velocity experienced by the meter
  - The Yaw rotation aligns the major axis of the data with the downstream flow direction
    - Periods of increased storm discharge were isolated and denoted the direction of rotation
      - These storm periods/increased downstream discharge were represented by the least common 5% of the TCM data
      - The slope between the centroids of the two clusters determine from the least common 5% of the data was aligned parallel to the Y-axis.
  - The resulting rotation meant that all positive Y-axis values denote downstream flow, while negative Y-axis values represented upstream flow.
- Tilts away from the origin was determined by the following equation:

$\theta={tan}^{-1}(\frac{|\left| vz x a \right||}{vz . a})$ -> where ‘vz . a’ is the ‘scalar/dot product’

where *vz* are the zero velocity observations, and *a* is each [x, y, z] vector observation. All vectors in this calculation have been normalized and rotated with the previous coordinate rotations.

- Flume-derived, empirical curves relate degrees from vertical (0-90) to a local specific discharge
  - Different curve depending upon TCM model
    - Initial purchase was all 1p50s
    - Replaced site M2 with a 1p50b15 in March of 2016
  - Some tilts above 90 degrees observed. However, those values were reset to 90 degrees which is the maximum for which we have empirical flume data.
- We broke down and recorded the discharge vector into its downstream (y-axis) and cross-stream (x-axis) specific discharges.
- The resulting data is a time series of 15 minute data specific discharge data stretching from 20 May 2015 to the present.
- The resulting TCM velocity data is accurate down to approximately 2 cm s^-1^ for any given sampling value [*Sheremet* *et al.* 2009, *Sheremet and Manning* 2013].
  - Although the exact accuracy of velocities of magnitude less than 2 cm s^-1^ is uncertain, the timing of tidal oscillations in discharge is sufficiently accurate to provide insight into the impact of tidal cycles (i.e., diurnal, semidiurnal) on the river reach’s discharge regime [*Maio et al.* 2016, *Aretxabaleta et al.* 2014].

## A.2 Matlab code for TCM calibration, normalization and rotation:

Any questions should be directed to author, Allan E. Jones, at email: [allan.e.jones@utexas.edu](mailto:allan.e.jones@utexas.edu)

%{

This script will replicate vitalii's python scripts to calibrate and

convert TCM tilt data into secific discharge.

Author: Allan Jones

Date: 6/27/2016

%}

close all; clear; clc;

cur_dir = cd; slashes = regexp(cur_dir, '\');

using_comp = cur_dir(1:slashes(3));

date_save = ['prelim_' datestr(now, 'yyyymmdd')];

plot_check = 01; % 0 for no plot save

fig = 1;

% save path for TCM and LTC data

save_path = [using_comp 'Dropbox\Field Work\Analysis\'...

'All Long Term Data\Matlab Cleaned Data\Calibrated TCM data\'...

date_save '\'];...

if ~isdir([save_path])

mkdir([save_path]);

end

% diary to record the output

diary([using_comp 'Dropbox\Field Work\Analysis\'...

'All Long Term Data\Matlab Cleaned Data\Calibrated TCM data\'...

date_save '\Rotation_diary.txt']);

%% correcting datenum python errors

%{

%% obtaining ADP data

disp(['Loading ADP data...'])

ADPdirectory = ['Dropbox\Field Work\'...

'Analysis\All Long Term Data\ADP_24hr\'];

river_folder = 'Aransas\';

ADPfnames24 = {'AR24_20150810.mat'; 'AR24_20160227.mat'};

for iii = 1:length(ADPfnames24)

load([using_comp ADPdirectory river_folder ADPfnames24{iii}]);

end

%% obtaining LEVEL data

disp(['Loading LTC data...'])

LTCdir = ['Dropbox\Field Work\Analysis\'...

'All Long Term Data\LTC data corrected\'];

LTCnames = dir([using_comp LTCdir]);

% find and load actual filenames -> 'from' date to data in real titles

for iii = 1:length(LTCnames)

if ~isempty(regexp(LTCnames(iii).name, 'from'))

load([using_comp LTCdir LTCnames(iii).name]);

end

end

%% obtaining TCM data

disp(['Loading TCM data...'])

TCMdir = ['Dropbox\Field Work\Analysis\'...

'All Long Term Data\TCM data\'];

TCMnames = dir([using_comp TCMdir]);

% find and load actual filenames -> 'from' date to data in real titles

for iii = 1:length(TCMnames)

if ~isempty(regexp(TCMnames(iii).name, 'from'))

load([using_comp TCMdir TCMnames(iii).name]);

end

end

%% obtaining an alphabetical list of the Mission and Aransas variables

AranVars = who('AR*');

MissVars = who('MR*');

%% Cleaning downloaded data - 4/18/2016

disp(['Converting TCM data to double...'])

%making fields of TCM data into doubles

fieldnames = {'X_Accel'; 'Y_Accel'; 'Z_Accel'; 'X_Tilt'; 'Y_Tilt';...

'Z_Tilt'; 'Sum_Vector'};

% for the Aransas River

for vvv = [find(not(cellfun('isempty',regexp(AranVars, 'TCM'))))]'

for iii = 1:length(fieldnames)

% converting string matrices to double

eval([AranVars{vvv} '.' fieldnames{iii} ' = '...

'str2num(' AranVars{vvv} '.' fieldnames{iii} ');'])

end

end

%%%%%%%%%%%%%%%%%%%%%%%%%%%%%%%%%%%%%%%%%%%%%%%%%%%%%%%%%%%%%%%%%%%%%%%%%%%

% RPEAT PROCESS FOR MISSION RIVER

%%%%%%%%%%%%%%%%%%%%%%%%%%%%%%%%%%%%%%%%%%%%%%%%%%%%%%%%%%%%%%%%%%%%%%%%%%%

% for the Miss River

for vvv = [find(not(cellfun('isempty',regexp(MissVars, 'TCM'))))]'

for iii = 1:length(fieldnames)

% converting string matrices to double

eval([MissVars{vvv} '.' fieldnames{iii} ' = '...

'str2num(' MissVars{vvv} '.' fieldnames{iii} ');'])

end

end

%% Resetting all the date numbers

disp(['Adjusting date numbers...'])

% Aransas River

for vvv = 1:length(AranVars)

% for the TCM and ADP files

if ~isempty(regexp(AranVars{vvv}, 'AR24')) ||...

~isempty(regexp(AranVars{vvv},'TCM'))

%resetting datenum variable

eval([AranVars{vvv} '.Datenum = [];'])

eval(['lengthy = length(' AranVars{vvv} '.Date_Time);'])

for iii = 1:lengthy

%recalculatting date numbers

eval([AranVars{vvv} '.Datenum(iii,1) = datenum('...

AranVars{vvv} '.Date_Time(iii,:));'])

end

else

% for the LTC files

% resetting date num variable

eval([AranVars{vvv} '.Datenum = [];'])

eval(['lengthy = length(' AranVars{vvv} '.Date);'])

% Turning temperature and level into vectors

eval([AranVars{vvv} '.TEMPERATURE = ' AranVars{vvv}...

'.TEMPERATURE'';'])

eval([AranVars{vvv} '.CONDUCTIVITY = ' AranVars{vvv}...

'.CONDUCTIVITY'';'])

eval([AranVars{vvv} '.LEVEL = ' AranVars{vvv} '.LEVEL'';'])

eval([AranVars{vvv} '.ms = ' AranVars{vvv} '.ms'';'])

for iii = 1:lengthy

% recalculating date numbers

eval([AranVars{vvv} '.Datenum(iii,1) = datenum(['...

AranVars{vvv} '.Date(iii,:) '' '' ' AranVars{vvv}...

'.Time(iii,:)]);'])

end

end

end

%%%%%%%%%%%%%%%%%%%%%%%%%%%%%%%%%%%%%%%%%%%%%%%%%%%%%%%%%%%%%%%%%%%%%%%%%%%

% REPEAT PROCESS FOR MISSION RIVER

%%%%%%%%%%%%%%%%%%%%%%%%%%%%%%%%%%%%%%%%%%%%%%%%%%%%%%%%%%%%%%%%%%%%%%%%%%%

% for the Miss River

for vvv = 1:length(MissVars)

% for the TCM and ADP files

if ~isempty(regexp(MissVars{vvv}, 'AR24')) ||...

~isempty(regexp(MissVars{vvv},'TCM'))

%resetting datenum variable

eval([MissVars{vvv} '.Datenum = [];'])

eval(['lengthy = length(' MissVars{vvv} '.Date_Time);'])

for iii = 1:lengthy

%recalculatting date numbers

eval([MissVars{vvv} '.Datenum(iii,1) = datenum('...

MissVars{vvv} '.Date_Time(iii,:));'])

end

else

% for the LTC files

% resetting date num variable

eval([MissVars{vvv} '.Datenum = [];'])

eval(['lengthy = length(' MissVars{vvv} '.Date);'])

% Turning temperature and level into vectors

eval([MissVars{vvv} '.TEMPERATURE = ' MissVars{vvv}...

'.TEMPERATURE'';'])

eval([MissVars{vvv} '.CONDUCTIVITY = ' MissVars{vvv}...

'.CONDUCTIVITY'';'])

eval([MissVars{vvv} '.LEVEL = ' MissVars{vvv} '.LEVEL'';'])

eval([MissVars{vvv} '.ms = ' MissVars{vvv} '.ms'';'])

for iii = 1:lengthy

% recalculating date numbers

eval([MissVars{vvv} '.Datenum(iii,1) = datenum(['...

MissVars{vvv} '.Date(iii,:) '' '' ' MissVars{vvv}...

'.Time(iii,:)]);'])

end

end

end

%% Save and clear ADP 24 hour data

disp(['Saving and clearing 24 hour data...'])

% create a path for saving data

adp_save_path = ['Dropbox\Field Work\Analysis\'...

'All Long Term Data\Matlab Cleaned Data\'];

data_saving = '24 Hour ADP Data\';

if ~isdir([using_comp adp_save_path data_saving])

mkdir([using_comp adp_save_path data_saving])

end

% finding 24 hour study variables

for vvv = find(not(cellfun('isempty',regexp(AranVars, 'AR24'))))'

% saving 24 hour data

eval(['save([using_comp adp_save_path data_saving AranVars{vvv} ''.mat''],'...

'''' AranVars{vvv} ''');'])

end

%%%%%%%%%%%%%%%%%%%%%%%%%%%%%%%%%%%%%%%%%%%%%%%%%%%%%%%%%%%%%%%%%%%%%%%%%%%

% REPEAT PROCESS FOR MISSION RIVER

%%%%%%%%%%%%%%%%%%%%%%%%%%%%%%%%%%%%%%%%%%%%%%%%%%%%%%%%%%%%%%%%%%%%%%%%%%%

% for the Miss River

% finding 24 hour study variables

for vvv = find(not(cellfun('isempty',regexp(MissVars, 'MR24'))))'

% saving 24 hour data

eval(['save([using_comp save_path data_saving MissVars{vvv} ''.mat''],'...

'''' MissVars{vvv} ''');'])

end

% clearing data

eval(['clear '...

AranVars{find(not(cellfun('isempty',regexp(AranVars, 'AR24'))))'}])

% eval(['clear '...

% MissVars{find(not(cellfun('isempty',regexp(MissVars, 'MR24'))))'}])

clear fieldnames ADPdirectory ADPfnames24

%% Save datenum corrected data

disp(['Saving datenum fixed data...'])

% create a path for saving data

save_datenum_path = ['Dropbox\Field Work\Analysis\All Long Term Data\'...

'Matlab Cleaned Data\'];

data_saving = 'Datenum cleaned Data\';

if ~isdir([using_comp save_datenum_path data_saving])

mkdir([using_comp save_datenum_path data_saving])

end

% obtaining updated list of datenum corrected variables

AranVars = who('AR*');

for vvv = 1:length(AranVars)

eval(['save([using_comp save_datenum_path data_saving AranVars{vvv} '...

'''_datenum_corrected.mat''],'...

'''' AranVars{vvv} ''');'])

end

%%%%%%%%%%%%%%%%%%%%%%%%%%%%%%%%%%%%%%%%%%%%%%%%%%%%%%%%%%%%%%%%%%%%%%%%%%%

% REPEAT PROCESS FOR MISSION RIVER

%%%%%%%%%%%%%%%%%%%%%%%%%%%%%%%%%%%%%%%%%%%%%%%%%%%%%%%%%%%%%%%%%%%%%%%%%%%

% for the Miss River

% obtaining updated list of datenum corrected variables

MissVars = who('MR*');

for vvv = 1:length(MissVars)

eval(['save([using_comp save_datenum_path data_saving MissVars{vvv} '...

'''_datenum_corrected.mat''],'...

'''' MissVars{vvv} ''');'])

end

%}

%% Loading the datenum corrected data

%7{

load_path = ['Dropbox\Field Work\Analysis\All Long Term Data\'...

'Matlab Cleaned Data\Datenum cleaned Data\'];

load_files = dir([using_comp load_path]);

for vvv = 1:length(load_files)

if ~isempty(regexp(load_files(vvv).name, '.mat'))

eval(['load([using_comp load_path ''' load_files(vvv).name '''])'])

end

end

%}

%% Reading in deployment dates and creating site structure variables

%7{

disp(['Obtaining deployment date information...'])

%opening the .csv deployment file

deployment_path = ['Dropbox\Field Work\Analysis\'...

'Field Data Analysis\Clean Field data\'];

deploy_file = 'TCM_deployments.csv';

Dfid = fopen([using_comp deployment_path deploy_file]);

soi = {};

%reading the information from each line

dummy = fgetl(Dfid); num = 1;

while ischar(dummy)

% pull dates from between commas and create variable of site name

commas = [1 regexp(dummy, ',') length(dummy)+1];

for iii = 1:length(commas)-1

if iii == 1

% creating variable (sturcture) of site name

sitevar = dummy(commas(iii):commas(iii+1)-1);

soi{num,1} = sitevar;

else

% storing the dates within the variable

eval([sitevar '.deploy_dates{iii-1,1} = '....

dummy(commas(iii)+1:commas(iii+1)-1) ';'])

end

end

% grab new line

dummy = fgetl(Dfid); num = num+1;

end

fclose(Dfid);

% clear data

clear num commas sitevar

%}

%% creating master matrices

%{

%% Creating trimmed matrix within structures

disp(['Trimming data based on deployment dates...'])

for vvv = 1:length(soi)

% creating a string to obtain previous variables

initials = [soi{vvv}(1) 'R_' soi{vvv}(2)];

% creating a matrices of all the raw data to trim

eval([initials '.trimmed_mat = [' initials '.Datenum,' initials '.LEVEL,'...

initials '.TEMPERATURE,' initials '.CONDUCTIVITY];'])

eval([initials 'TCM.trimmed_mat = [' initials 'TCM.Datenum,'...

initials 'TCM.X_Accel,'...

initials 'TCM.X_Tilt,'...

initials 'TCM.Y_Accel,'...

initials 'TCM.Y_Tilt,'...

initials 'TCM.Z_Accel,'...

initials 'TCM.Z_Tilt,'...

initials 'TCM.Sum_Vector];']);

% find indices to remove observations before initial install

eval(['remove = find(' initials '.Datenum < datenum('...

soi{vvv} '.deploy_dates{1},''yyyymmdd HHMM''));'])

eval(['remove_t = find(' initials 'TCM.Datenum < datenum('...

soi{vvv} '.deploy_dates{1},''yyyymmdd HHMM''));'])

% find indices to remove for final observations

eval(['remove = [remove;'...

'find(' initials '.Datenum > datenum('...

soi{vvv} '.deploy_dates{end},''yyyymmdd HHMM''))];'])

eval(['remove_t = [remove_t;'...

'find(' initials 'TCM.Datenum > datenum('...

soi{vvv} '.deploy_dates{end},''yyyymmdd HHMM''))];'])

% removing indices

eval([initials '.trimmed_mat(remove,:) = [];'])

eval([initials 'TCM.trimmed_mat(remove_t,:) = [];'])

end

%% Creating master (timestamp aligned) matrix within the site structures

disp(['Creating master matrix of all data for each site...'])

mins15 = 15/(60*24);

for vvv = 1:length(soi)

% creating blank master matrix

eval([soi{vvv} '.master = [];'])

eval(['june15_deploy = datenum(' soi{vvv} '.deploy_dates{2},'...

'''YYYYmmdd HHMM'');'])

% obtaining "initials"

initials = [soi{vvv}(1) 'R_' soi{vvv}(2)];

%finding length of trimmed matrices

eval(['lengthy = length(' initials '.trimmed_mat);'])

for iii = 1:lengthy % searching LTC data for TCM match

% obtaining LTC timestamp

eval(['[time_diff,TCM_index] = min(abs(' initials...

'.trimmed_mat(iii,1) - ' initials 'TCM.trimmed_mat(:,1)));'])

% **** UPDATE IF EVER FIGURE OUT HOW TO USE IMPROPER INSTALL DATA ****

% removing TCM values before June installation

eval(['timer = ' initials '.trimmed_mat(iii,1);'])

if timer < june15_deploy

time_diff = 1e6; % forces next if statement to record nans

end

% if nearest data point is more than 15 minutes away, store nan values

if time_diff > mins15

% storing nan values in the master variable

eval([soi{vvv} '.master = ['...

soi{vvv} '.master;'...

initials '.trimmed_mat(iii,:),'...

'nan(1,size(' initials 'TCM.trimmed_mat,2))];'])

else

% storing TCM values in the master variable

eval([soi{vvv} '.master = ['...

soi{vvv} '.master;'...

initials '.trimmed_mat(iii,:),'...

initials 'TCM.trimmed_mat(TCM_index,:)];'])

end

end

%saving header information

eval([soi{vvv} '.headers = {''LTC Datenum'';'...

'''LTC Level [m]''; ''LTC Temp [C]''; ''LTC Cond uS/cm'';'...

'''TCM Datenum''; ''TCM X Accel''; ''TCM X Tilt'';'...

'''TCM Y Accel''; ''TCM Y Tilt'';'...

'''TCM Z Accel''; ''TCM Z Tilt'';'...

'''TCM Sum Vector''};'])

% remove repeated timesteps - TCM or LTC - sort the matrix by LTC timestamp

% removing repeat TCM timestamps

eval(['[~, ui, ~] = unique(' soi{vvv} '.master(:,5));'])

eval([soi{vvv} '.master = ' soi{vvv} '.master(ui,:);'])

% removing repeat LTC timestamps

eval(['[~, ui, ~] = unique(' soi{vvv} '.master(:,1));'])

eval([soi{vvv} '.master = ' soi{vvv} '.master(ui,:);'])

end

%% save master matrices

disp(['Saving Master data...'])

% create a path for saving data

save_master_path = ['Dropbox\Field Work\Analysis\All Long Term Data\'...

'Matlab Cleaned Data\'];

data_saving = 'initial Master matrices\';

if ~isdir([using_comp save_master_path data_saving])

mkdir([using_comp save_master_path data_saving])

end

for vvv = 1:length(soi)

eval(['save([using_comp save_master_path data_saving soi{vvv} '...

'''_initial_master.mat''],'...

'''' soi{vvv} ''');'])

end

%}

%% load saved Master data

disp(['Loading initial Master data...'])

% create a path for saving data

load_master_path = ['Dropbox\Field Work\Analysis\All Long Term Data\'...

'Matlab Cleaned Data\'];

data_saving = 'initial Master matrices\';

for vvv = 1:length(soi)

eval(['load([using_comp load_master_path data_saving '...

'soi{vvv} '...

'''_initial_master.mat''])'])

end

%% removing unneeded data

% removing the unneeded raw tilt and sum vector data

disp(['Removing the unnecessary raw tilt and sum vector data...'])

unneeded_indices = [7 9 11 12];

for vvv = 1:length(soi)

eval([soi{vvv} '.master(:,unneeded_indices) = [];'])

eval([soi{vvv} '.headers = {''LTC Datenum'';'...

'''LTC Level [m]''; ''LTC Temp [C]''; ''LTC Cond uS/cm'';'...

'''TCM Datenum''; ''TCM X Accel'';'...

'''TCM Y Accel'';'...

'''TCM Z Accel''};'])

end

%% cleaning data from install events and storing in a 'split' cell/matrix

disp(['Cleaning out the data from between deployments...'])

for vvv = 1:length(soi)

eval([soi{vvv} '.split_master ={};'])

%finding # of deplyments

eval(['lengthy = length(' soi{vvv} '.deploy_dates);'])

storage = 1;

for iii = 1:lengthy-1

% grabbing date of deployment

eval(['dateup = ' soi{vvv} '.deploy_dates{iii+1};'])

if iii == 1

% obtaining the LTC timestamps between the first and second install timestamp

% (-1 hr for install time)

eval(['LTC_timestamps = '...

'intersect('...

'find(' soi{vvv} '.master(:,1) > datenum('...

soi{vvv} '.deploy_dates{iii}, ''yyyymmdd HHMM'')),'...

'find(' soi{vvv} '.master(:,1) < datenum('...

soi{vvv} '.deploy_dates{iii+1}, ''yyyymmdd HHMM'')-0.25/24));'])

% obtaining the TCM timestamps between the first and second install timestamp

TCM_timestamps = []; % bad first installation in May 2015

% finding and storing the common values

indices = LTC_timestamps;

eval([soi{vvv} '.split_master{iii,1} = '...

soi{vvv} '.master(indices,:);'])

% stripping extra data off of end of July 2016 downloads

elseif ~isempty(regexp(dateup, '20160716')) ||...

~isempty(regexp(dateup, '20160717'))

% storing nan values for period of calibrations in July 2016

eval([soi{vvv} '.split_master{iii,1} = nan(10,8);'])

% normal conditions

else

% obtaining the LTC timestamps between the current

% (+1 hr for install time) and next install timestamp

eval(['LTC_timestamps = '...

'intersect('...

'find(' soi{vvv} '.master(:,1) > datenum('...

soi{vvv} '.deploy_dates{iii}, ''yyyymmdd HHMM'')+0.5/24),'...

'find(' soi{vvv} '.master(:,1) < datenum('...

soi{vvv} '.deploy_dates{iii+1}, ''yyyymmdd HHMM'')-0.25/24));'])

% obtaining the TCM timestamps between the current

%(+1 hr for install time) and next install timestamp

eval(['TCM_timestamps = '...

'intersect('...

'find(' soi{vvv} '.master(:,5) > datenum('...

soi{vvv} '.deploy_dates{iii}, ''yyyymmdd HHMM'')+0.5/24),'...

'find(' soi{vvv} '.master(:,5) < datenum('...

soi{vvv} '.deploy_dates{iii+1}, ''yyyymmdd HHMM'')-0.25/24));'])

% finding and storing the common values

% if ~isempty(TCM_timestamps) && ...

% abs(length(LTC_timestamps)-length(TCM_timestamps)) < 5

% indices = intersect(LTC_timestamps, TCM_timestamps);

% else

% indices = LTC_timestamps;

% end

% the above script forces a match between indices before saving the data.

% instead, we will take the set of indices that has more points and save

% those in the split_master. the above script assumed the TCMs would be

% more reliable than they were.

% - i.e., likely LTC will have more points, and we want as much data from

% all sensors, even if that means that the LTC data is unmatched with TCM

% data

if length(TCM_timestamps) > length(LTC_timestamps)

indices = TCM_timestamps;

else

indices = LTC_timestamps;

end

eval([soi{vvv} '.split_master{iii,1} = '...

soi{vvv} '.master(indices,:);'])

end

end

end

%% Creating empirical tilt curves

disp(['Creating the empirical tilt curves...'])

% making tilt curve for the 1p50b15 TCM

b15 = [0 0;... matrix of [tilt (deg), velocity (cm/s)] columns

5 4.7310;...

10 8.2004;...

20 13.8776;...

30 18.9240;...

40 25.2320;...

50 32.1708;...

60 41.6328;...

70 57.4028;...

80 100.9280;...

90 170.3160];

% making tilt curve for the 1p50 TCM

p50 = [0 0;... matrix of [tilt (deg), velocity (cm/s)] columns

5 8.8320;...

10 15.3088;...

20 25.9072;...

30 35.3280;...

40 47.1040;...

50 58.8800;...

60 77.7216;...

70 103.0400;...

80 160.1536;...

90 317.9520];

% figure(1)

% subplot(1,2,1)

% plot(b15(:,1), b15(:,2), 'marker','.', 'markersize', 20)

% xlabel('Tilt degrees(\circ)')

% ylabel('Velocity, [cm s^{-1}]')

% title('Ballasted TCM')

%

%

% subplot(1,2,2)

% plot(p50(:,1), p50(:,2), 'marker','.', 'markersize', 20)

% xlabel('Tilt degrees(\circ)')

% ylabel('Velocity, [cm s^{-1}]')

% title('Non-ballasted TCM')

%% translate from accel, g into deg then into q

disp(['Rotating and translating accel (g) to tilt then q...'])

%{

The section performs the following actions:

- normalizes the data first

- centers the data on the normalized V0

- then performs a YAW rotation

- all rotations also performed on V0 values (should end at origin

- calculates tilt (deg from vert) and velocity

- sets all velocities with direction > 0 as positive, and all those < 0 as

negative

- denoting downstream and upstream flow, repsectively

%}

% Rotate Cartesian coordinates so y-axis aligns with river flow direction

% y+ points downstream, X+ points toward the right bank, z+ points up

% assuming that Z is properly oriented in the veritical direction

% performing a "yaw" rotation

% ^^-> Heinesch 2006 - Coordinate rotation presentation (Rotations_ppt.tif)

% p/slide 9-11 - Rotations 1-3 matrix

rotXY = @(alpha)...

[cosd(alpha) sind(alpha) 0;...

-sind(alpha) cosd(alpha) 0;...

0 0 1];

rotXZ = @(beta)...

[cosd(beta) 0 sind(beta);...

0 1 0;...

-sind(beta) 0 cosd(beta)];

rotYZ = @(gamma)...

[1 0 0;...

0 cosd(gamma) sind(gamma);...

0 -sind(gamma) cosd(gamma)];

% ** we need to perform a YXZ rotation, or pitch, roll, then yaw rotations

% loading the calibration material

calib_path = [using_comp '\Dropbox\Field Work\Analysis\'...

'All Long Term Data\Configurate TCM\'];

% loop through sites and translate accel data into tilt

for vvv = 1:length(soi)

% displaying the current site being worked on

disp(['Working site ' soi{vvv} '...'])

eval(['splits = length(' soi{vvv} '.deploy_dates)-1;'])

for kkk = 1:splits

% obtaining data for current field installation

eval(['current_data = ' soi{vvv} '.split_master{kkk,1};'])

% grabbing date for titles of plots

eval(['title_date = ' soi{vvv} '.deploy_dates{kkk,1}(1:8);'])

% if ALL the values are nan, skip the calculations

% sum of valid data is greater than 100 points

if sum(~isnan(current_data(:,5))) > 20

% TCM has left-handed coordinate system

% convert to right-handed

ttt = current_data(:,5); % time stamps

XYZ_raw = [current_data(:,8) current_data(:,7) current_data(:,6)];

% [cross-channel up\down-stream vertical]

% obtaining the calibration/configuration coefficients to

% normalize the raw data before rotation

if strcmp(soi{vvv}, 'M2')

%obtaining proper coefs

load([calib_path soi{vvv} '_1p50b15_calib_coef.mat']);

% equation coefficients

emp_curve = b15;

elseif datenum(title_date, 'yyyymmdd') > datenum('20160716', 'yyyymmdd')...

&& strcmp(soi{vvv},'M1')

disp(['Ballasted logger moved to M1 in summer of 2016.'])

disp(['Using ballasted configuration coefficients.'])

%obtaining proper coefs

load([calib_path 'M2_1p50b15_calib_coef.mat']);

% equation coefficients

emp_curve = b15;

elseif strcmp(soi{vvv},'M1')

disp(['No cfg data available for M1'])

disp(['Using M3 configuration coefficients.'])

% obtaining M3 coefs

load([calib_path 'M3_1p50_calib_coef.mat']);

% equation coefficients

emp_curve = p50;

else

% obtaining correct coefs

load([calib_path soi{vvv} '_1p50_calib_coef.mat']);

% equation coefficients

emp_curve = p50;

end

% ************* % finding most commonly repeated data

% ************* % with X-,Y-,and Z-data all the same

iq_test = XYZ_raw;

ordered_TCM = nan(length(iq_test),2);

for jjj = 1:length(iq_test)

if isnan(ordered_TCM(jjj))

% find number of cells that all have same X, Y, & Z

xi = find(abs(iq_test(:,1) - iq_test(jjj,1)) < 1e-10);

xyi = xi(find(abs(iq_test(xi,2) - iq_test(jjj,2)) < 1e-10));

xyzi = xyi(find(abs(iq_test(xyi,3) - iq_test(jjj,3)) < 1e-10));

% track and store number of cells

if length(xyzi) >= 1

ordered_TCM(xyzi,1) = jjj;

ordered_TCM(xyzi,2) = length(xyzi);

elseif isnan(iq_test(jjj,1))

ordered_TCM(xyzi,1) = jjj;

ordered_TCM(xyzi,2) = nan;

% nans wil be removed from dataset to not impact 95%

else

error(['Data is not finding a match, even to itself.'])

end

end

end

% attach timestep counter to data

ordered_TCM = [[1:1:length(ordered_TCM)]', ordered_TCM];

[~,sorti] = sort(ordered_TCM(:,3), 'descend');

ordered_TCM = ordered_TCM(sorti,:);

ordered_TCM = ordered_TCM(~isnan(ordered_TCM(:,2)),:);

% finding most common 25% points

iqp100 = 0.25;

quant_num = round(length(ordered_TCM(:,1))*iqp100);

origin_prime = ordered_TCM(1:quant_num,1);

% finding median x,y,z values of most common 25% of points

if strcmp(soi{vvv}, 'A2')

V0X = nanmean(XYZ_raw(origin_prime, 1));

V0Y = nanmean(XYZ_raw(origin_prime, 2));

V0Z = nanmean(XYZ_raw(origin_prime, 3));

elseif strcmp(soi{vvv}, 'A3') && strcmp(title_date, '20161203')

V0X = nanmean(XYZ_raw(origin_prime, 1));

V0Y = nanmean(XYZ_raw(origin_prime, 2));

V0Z = nanmean(XYZ_raw(origin_prime, 3));

end

%********** % Normalizing the data

XYZ_norm = [(XYZ_raw(:,1)-QX0)./QX1,...

(XYZ_raw(:,2)-QY0)./QY1,...

(XYZ_raw(:,3)-QZ0)./QZ1];

% normalizing the zero velocity data

vz_norm = [(V0X - QX0)./QX1; (V0Y - QY0)./QY1; (V0Z - QZ0)./QZ1];

y_rot_pt = vz_norm;

% inverting XYZ matrix

XYZ_norm = XYZ_norm';

% ************* % rotating the XZ coordinate plane about Y!!- AXIS

% ************* % rotating about the most common 25% of points

centroid_slope = y_rot_pt(3)/y_rot_pt(1);

if centroid_slope > 0 % positive

beta = atand(centroid_slope)-90;

else % negative

beta = atand(centroid_slope)+90;

end

eval([soi{vvv} '.rotation_degs(kkk,1) = beta;'])

cartesian_y_rotate = rotXZ(beta)*XYZ_norm;

x_rot_pt = rotXZ(beta)*y_rot_pt;

vz1 = rotXZ(beta)*vz_norm;

% ************* % rotating the YZ coordinate plane about X!!- AXIS

% ************* % rotating about the most common 25% of points

centroid_slope = x_rot_pt(3)/x_rot_pt(2);

if centroid_slope > 0 % positive

gamma = atand(centroid_slope)-90;

else % negative

gamma = atand(centroid_slope)+90;

end

eval([soi{vvv} '.rotation_degs(kkk,2) = gamma;'])

cartesian_yx_rotate = rotYZ(gamma)*cartesian_y_rotate;

z_rot_pt = rotYZ(gamma)*x_rot_pt;

vz2 = rotYZ(gamma)*vz1;

% ************* % YAW rotation using median X&Y of the farthest 1 percent

% ************* % rotating the XY coordinate plane about Z!!- AXIS

% finding least common 5% of points

iqp100 = 0.95;

quant_num = round(length(ordered_TCM(:,1))*iqp100);

ind05 = ordered_TCM(quant_num:end,1);

ind95 = ordered_TCM(1:quant_num, 1);

least_common = cartesian_yx_rotate(:,ind05)';

% determine if there are two unique clusters of farthest one percent

disp([soi{vvv} ': ' title_date])

[clus_ind, Centroids] = kmeans(least_common(:,1:2), 2,...

'Replicates', 10, 'Display', 'final');

% determine rotation slope from centroid data

% We will rotate with centroid that is further from origin.

if sqrt(sum(Centroids(1,:).^2)) > sqrt(sum(Centroids(2,:).^2))

interp_pt = Centroids(1,:);

other_pt = Centroids(2,:);

if (vvv == 4 || vvv == 5) && iii == splits

interp_pt = Centroids(2,:);

other_pt = Centroids(1,:);

end

else

interp_pt = Centroids(2,:);

other_pt = Centroids(1,:);

if (vvv == 4 || vvv == 5) && iii == splits

interp_pt = Centroids(1,:);

other_pt = Centroids(2,:);

end

end

% Manual adjustments to problem sites

if strcmp(soi{vvv}, 'A2') && strcmp(title_date , '20160111')

interp_pt = Centroids(find(Centroids(:,2) < 0),:);

other_pt = Centroids(find(Centroids(:,2) > 0),:);

elseif strcmp(soi{vvv}, 'A4') && strcmp(title_date , '20160430')

interp_pt = Centroids(find(Centroids(:,2) > 0),:);

other_pt = Centroids(find(Centroids(:,2) < 0),:);

elseif strcmp(soi{vvv}, 'A5') && strcmp(title_date , '20150808')

interp_pt = Centroids(find(Centroids(:,2) < 0),:);

other_pt = Centroids(find(Centroids(:,2) > 0),:);

elseif strcmp(soi{vvv}, 'A5') && strcmp(title_date , '20160430')

interp_pt = Centroids(find(Centroids(:,1) > 0),:);

other_pt = Centroids(find(Centroids(:,1) < 0),:);

end

% obtaining clusters to plot

plot_clusters = {[least_common(clus_ind==1,1), least_common(clus_ind==1,2)];...

[least_common(clus_ind==2,1), least_common(clus_ind==2,2)]};

cluster_cols = {'om','oc'};

% find slope from selected interpolation point and rotate

centroid_slope = (interp_pt(2)-other_pt(2))/(interp_pt(1)-other_pt(1)); % obtaining the slope between centroids

quad_points = [interp_pt(2)/centroid_slope, interp_pt(2)];

if quad_points(1)>0 && quad_points(2)>0 % quadrant 1

alpha = atand(centroid_slope)-90;

plot_slope = [-0.25:0.05:0.25; centroid_slope*(-0.25:0.05:0.25)];

no_rot = 0;

elseif quad_points(1)<0 && quad_points(2)>0 % quadrant 2

alpha = atand(centroid_slope)+90;

plot_slope = [0.25:-0.05:-0.25; centroid_slope*(0.25:-0.05:-0.25)];

no_rot = 0;

elseif quad_points(1)<0 && quad_points(2)<0 % quadrant 3

alpha = atand(centroid_slope)+90;

plot_slope = [0.25:-0.05:-0.25; centroid_slope*(0.25:-0.05:-0.25)];

no_rot = 0;

elseif quad_points(1)>0 && quad_points(2)<0 % quadrant 4

alpha = atand(centroid_slope)-90;

plot_slope = [-0.25:0.05:0.25; centroid_slope*(-0.25:0.05:0.25)];

no_rot = 0;

end

loc_str = 'SouthOutside';

% rotating the XY coordinate plane about Z-axis

eval([soi{vvv} '.rotation_degs(kkk,3) = alpha;'])

cartesian_prime = rotXY(alpha)*cartesian_yx_rotate;

final_origin = rotXY(alpha)*z_rot_pt;

vz_final = rotXY(alpha)*vz2;

% Plotting Rotation Procedure

% SUBPLOT 1 - raw data

figure(fig)

subplot(1,3,1)

plot(XYZ_raw(:,1), XYZ_raw(:,2), '.k')

hold on

plot(XYZ_norm(1,:), XYZ_norm(2,:), '.b')

% plotting most common 25%

plot(y_rot_pt(1),y_rot_pt(2), '.g', 'markersize',10)

plot(V0X, V0Y, '*r')

plot([QX0 QX1], [QY0 QY1], '*m')

title([soi{vvv} ': ' title_date])

% plotting axes

plot([-50 50], [0 0], 'color', [0.5 0.5 0.5]) % x-axis

plot([0 0], [-50 50], 'color', [0.5 0.5 0.5]) % y-axis

axis([-1.5 1.5 -1.5 1.5])

if strcmp(soi{vvv}, 'A2')

legend('Raw data', 'Normalized Data',...

'Normalized Origin - Raw 25% most repeated',...

'original - Vzero', 'original - Q coeffs',...

'Location', loc_str)

else

legend('Raw data', 'Normalized Data',...

'Normalized Origin',...

'original - Vzero', 'original - Q coeffs',...

'Location', loc_str)

end

% SUBPLOT 2 - origin corrected data

subplot(1,3,2)

plot(cartesian_yx_rotate(1,:), cartesian_yx_rotate(2,:), '.b')

hold on

% plotting data of least common 5%

% plot(cartesian_yx_rotate(1,iq3d_ind), cartesian_yx_rotate(2,iq3d_ind), 'og')

title([soi{vvv} ': ' title_date])

% plot centroid/cluster information

plot(plot_clusters{1}(:,1), plot_clusters{1}(:,2), cluster_cols{1})

plot(plot_clusters{2}(:,1), plot_clusters{2}(:,2), cluster_cols{2})

plot(plot_slope(1,:), plot_slope(2,:), 'r')

plot(quad_points(1), quad_points(2), '.r', 'markersize', 20)

plot(Centroids(:,1), Centroids(:,2), 'kx',...

'MarkerSize',10,'LineWidth',2)

plot(final_origin(1), final_origin(2), '*g')

% plotting axes

plot([-50 50], [0 0], 'color', [0.5 0.5 0.5]) % x-axis

plot([0 0], [-50 50], 'color', [0.5 0.5 0.5]) % y-axis

axis([-1.5 1.5 -1.5 1.5])

legend('Pitch/Roll data', '5% Cluster 1', '5% Cluster 2', 'Rotation Line', ...

'Rotation Point','Cluster centroids','Final Origin',...

'Location', loc_str)

% SUBPLOT 3 - the final rotated data

subplot(1,3,3)

plot(cartesian_prime(1,:), cartesian_prime(2,:), '.b')

hold on

plot(vz_final(1), vz_final(2), '*r')

plot([-50 50], [0 0], 'color', [0.5 0.5 0.5]) % x-axis

plot([0 0], [-50 50], 'color', [0.5 0.5 0.5]) % y-axis

axis([-1.5 1.5 -1.5 1.5])

if no_rot

text(0.3, 0.45, 'No rotation occured.')

end

title([soi{vvv} ': ' title_date])

legend('Rotated Data', 'Vzero - final',...

'Location', loc_str)

hold on

if plot_check ~= 0

% save plot

% *** SAVING FIGURE BEFORE MOVING ON

% makes sure the saved pdf fits within a landscape document

set(gcf,'PaperOrientation','landscape');

set(gcf,'PaperUnits','normalized');

set(gcf,'PaperPosition', [0 0 1 1]);

% Saving within the main folder not an individual river name folder

% legend off

folders = ['Rotation Method plots\' soi{vvv} '\'];

if ~isdir([save_path folders])

mkdir([save_path folders]);

end

% save as .tif

eval(['print(gcf, ''-dtiff'' , [save_path folders '...

'''' soi{vvv} '_' title_date '.tif'']);'])

% closing figures

% close all;

end

fig = fig +1;

%%%%%%%%%%%%%%%%%%%%%%%%%% - Finished rotating data ^^^

%%%%%%%%%%%%%%%%%%%%%%%%%% - Now calculating tilt and q vvvv

% creating storage variables

theta = nan(length(cartesian_prime(1,:)), 1);

qdis = nan(length(cartesian_prime(1,:)), 1);

for iii = 1:length(cartesian_prime(1,:))

% finding the tilt degress

dots = dot(vz_final, cartesian_prime(:,iii));

norman = norm(cross(vz_final, cartesian_prime(:,iii)));

theta(iii) = atan2d(norman, dots); % degrees

if theta(iii) > 90

theta(iii) = 90;

end

if isnan(theta(iii))

qdis(iii) = nan;

elseif max(theta(iii) == emp_curve(:,1))

% recording discharge of an exact calculated tilt

ind = find(emp_curve(:,1) == theta(iii));

qdis(iii) = emp_curve(ind,2);

else

% finding nearest tilts in the empirical curve

nearest = [max(find(emp_curve(:,1) <= theta(iii))),...

min(find(emp_curve(:,1) >= theta(iii)))];

% finding discharge data

newq = interp1(emp_curve(nearest,1), emp_curve(nearest,2),...

[emp_curve(nearest(1),1) full(theta(iii)) emp_curve(nearest(2),1)]);

qdis(iii) = newq(2);

end

end

downstream_flow = nan(size(qdis));

crossstream_flow = nan(size(qdis));

% setting q positive (+Y) for downstream flow

% and negative (-Y) for upstream flow

cartesian_prime = cartesian_prime'; % transposing data for easier calculations

posi = find(cartesian_prime(:,2) > 0);

nega = find(cartesian_prime(:,2) < 0);

downstream_flow(posi) = qdis(posi).*cosd(...

atand(cartesian_prime(posi,1)./cartesian_prime(posi,2)));

downstream_flow(nega) = -1*qdis(nega).*cosd(...

atand(cartesian_prime(nega,1)./cartesian_prime(nega,2)));

% recording cross-stream velocities

% Right bank (X+) = positive q

% Left bank (X-) = negative q

posix = find(cartesian_prime(:,1) > 0);

negax = find(cartesian_prime(:,1) < 0);

crossstream_flow(posix) = qdis(posix).*cosd(...

atand(cartesian_prime(posix,2)./cartesian_prime(posix,1)));

crossstream_flow(negax) = -1*qdis(negax).*cosd(...

atand(cartesian_prime(negax,2)./cartesian_prime(negax,1)));

cartesian_prime = cartesian_prime'; % re-transposing data to original form

% removing obvious outliers

if strcmp(soi{vvv}, 'M3') && strcmp(title_date , '20150808')

% finding outlier

out_ind = find(cartesian_prime(1,:) > 0.7);

% replacing with nans everywhere

XYZ_norm(:,out_ind) = [nan; nan; nan];

cartesian_prime(:,out_ind) = [nan; nan; nan];

theta(out_ind) = nan;

qdis(out_ind) = nan;

downstream_flow(out_ind) = nan;

crossstream_flow(out_ind) = nan;

elseif strcmp(soi{vvv}, 'M4') && strcmp(title_date , '20150808')

% finding outlier

out_ind = find(cartesian_prime(1,:) > 0.35);

% replacing with nans everywhere

XYZ_norm(:,out_ind) = [nan; nan; nan];

cartesian_prime(:,out_ind) = [nan; nan; nan];

theta(out_ind) = nan;

qdis(out_ind) = nan;

downstream_flow(out_ind) = nan;

crossstream_flow(out_ind) = nan;

end

%%%% plotting the data if applicable

%7{

% plotting the X Y data

figure(fig)

plot(cartesian_prime(1,:), cartesian_prime(2,:), '.')

hold on

plot(cartesian_prime(1,nega), cartesian_prime(2,nega),'oc')

plot(vz_final(1), vz_final(2), '*r')

lims = [get(gca, 'xlim'); get(gca, 'ylim')];

plot([-50 50], [0 0], 'color', [0.5 0.5 0.5]) % x-axis

plot([0 0], [-50 50], 'color', [0.5 0.5 0.5]) % y-axis

set(gca, 'xlim', lims(1,:))

set(gca, 'ylim', lims(2,:))

ylabel('Y')

xlabel('X')

title([soi{vvv} ': ' title_date])

if ~isempty(nega)

legend('Normalized/Rotated Raw Data','Negative q',...

'V-zero','Rotated Calib Coeff', 'Location','SouthOutside')

else

legend('Normalized/Rotated Raw Data',...

'V-zero','Rotated Calib Coeff','Location','SouthOutside')

end

grid on

% saving figures of all major axes

if plot_check ~= 0;

% *** SAVING FIGURE BEFORE MOVING ON

% makes sure the saved pdf fits within a landscape document

set(gcf,'PaperOrientation','landscape');

set(gcf,'PaperUnits','normalized');

set(gcf,'PaperPosition', [0 0 1 1]);

% save as .tif

print(gcf, '-dtiff' , [save_path folders soi{vvv} '_' title_date '_xVy.tif'])

end

fig = fig +1;

% plotting the discharge data

figure(fig)

plot(ttt, downstream_flow, '.')

hold on

plot(get(gca, 'xlim'), [ 2 2], '--r')

plot(get(gca, 'xlim'), [-2 -2], '--r')

ylabel('Velocity, (cm/s)')

set(gca, 'xticklabel', datestr(get(gca, 'xtick'),'mm-dd-yy'))

title([soi{vvv} ': ' title_date])

grid on

% saving figures of all major axes

if plot_check ~= 0;

% *** SAVING FIGURE BEFORE MOVING ON

% makes sure the saved pdf fits within a landscape document

set(gcf,'PaperOrientation','landscape');

set(gcf,'PaperUnits','normalized');

set(gcf,'PaperPosition', [0 0 1 1]);

% save as .tif

print(gcf, '-dtiff' , [save_path folders soi{vvv} '_' title_date '_qVt.tif'])

end

fig = fig +1;

%}

%%%%%%%%%%%% Storing the data

% storing updated acceleration data, tilt data, and discharge data

eval([soi{vvv} '.split_master{kkk}(:,end+1:end+3) = XYZ_norm'';'])

eval([soi{vvv} '.split_master{kkk}(:,end+1:end+3) = cartesian_prime'';'])

eval([soi{vvv} '.split_master{kkk}(:,end+1) = full(theta);'])

eval([soi{vvv} '.split_master{kkk}(:,end+1) = full(qdis);'])

eval([soi{vvv} '.split_master{kkk}(:,end+1) = downstream_flow;'])

eval([soi{vvv} '.split_master{kkk}(:,end+1) = crossstream_flow;'])

% storing nans if the X, Y, and Z values are nan values

else

% storing nan values

eval([soi{vvv} '.split_master{kkk}(:,end+1:end+10)'...

'= nan(length(current_data(:,5)),10);'])

end

end

% updating headers

eval([soi{vvv} '.headers{end+1,1} = ''TCM X normalized?'';'])

eval([soi{vvv} '.headers{end+1,1} = ''TCM Y normalized?'';'])

eval([soi{vvv} '.headers{end+1,1} = ''TCM Z normalized?'';'])

eval([soi{vvv} '.headers{end+1,1} = ''X rotated accel,g'';'])

eval([soi{vvv} '.headers{end+1,1} = ''Y rotated accel,g'';'])

eval([soi{vvv} '.headers{end+1,1} = ''Z rotated accel,g'';'])

eval([soi{vvv} '.headers{end+1,1} = ''abs(degrees from vetrical)'';'])

eval([soi{vvv} '.headers{end+1,1} = ''abs(specific discharge), cm/s'';'])

eval([soi{vvv} '.headers{end+1,1} = ''Specific dis along downstream '...

'(Y) axis, (cm/s)'';'])

eval([soi{vvv} '.headers{end+1,1} = ''Specific dis cross-stream '...

'(X) axis, (cm/s)'';'])

% creating a note

eval([soi{vvv} '.downstream_flow_note = '...

'''+ is downstream, - is upstream'';'])

eval([soi{vvv} '.crossstream_flow_note = '...

'''+ is Right bank, - is left bank'';'])

% freeing up memory space

close all

end

%% save updated matlab structures

disp(['Saving cleaned LTC and rotated TCM data...'])

for vvv = 1:length(soi)

eval(['save([save_path ''' soi{vvv}...

'_calib.mat''], ''' soi{vvv} ''')'])

end

% updating user on completion of script

disp(['Done.'])

diary off

# B. Summary of Fast Fourier Transforms (FFTs)


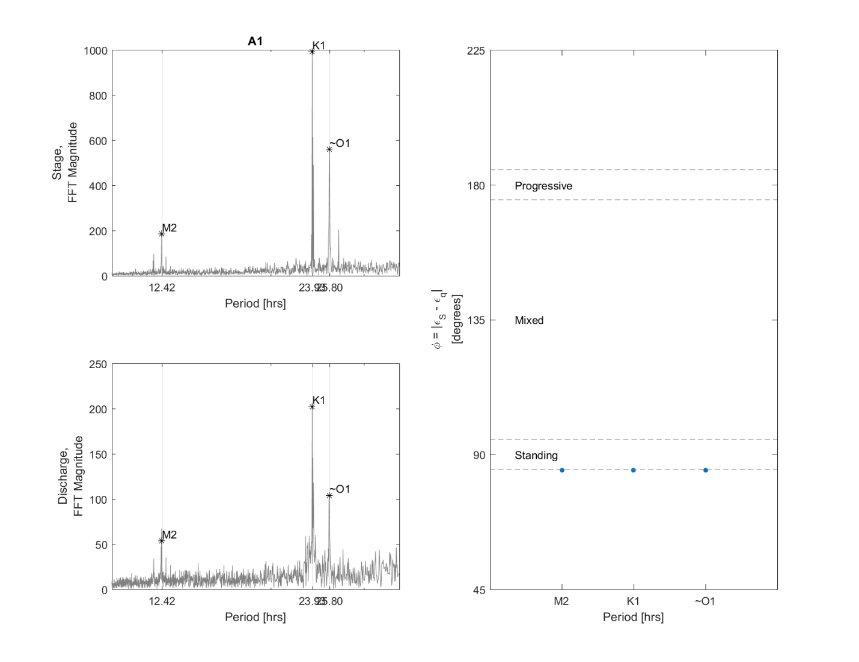


**Figure B.1**: The phase analysis for M-A site A1. The two subfigures (one atop the other) on the left-hand side of this figure, show the FFT magnitudes of the stage (left-top) and discharge (left-bottom) for the identified semidiurnal and diurnal harmonics analyzed for phase offset. The right-hand figure shows the calculated phase offset for each harmonic and compares it to the predicted offsets for a standing, mixed and progressive wave.


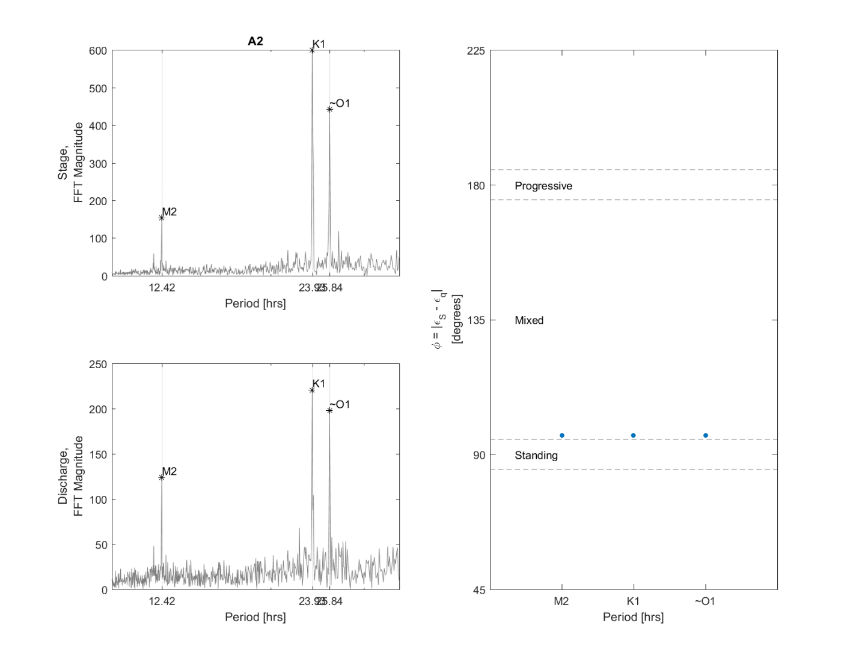


**Figure B.2**: The phase analysis for M-A site A2. The two subfigures (one atop the other) on the left-hand side of this figure, show the FFT magnitudes of the stage (left-top) and discharge (left-bottom) for the identified semidiurnal and diurnal harmonics analyzed for phase offset. The right-hand figure shows the calculated phase offset for each harmonic and compares it to the predicted offsets for a standing, mixed and progressive wave.


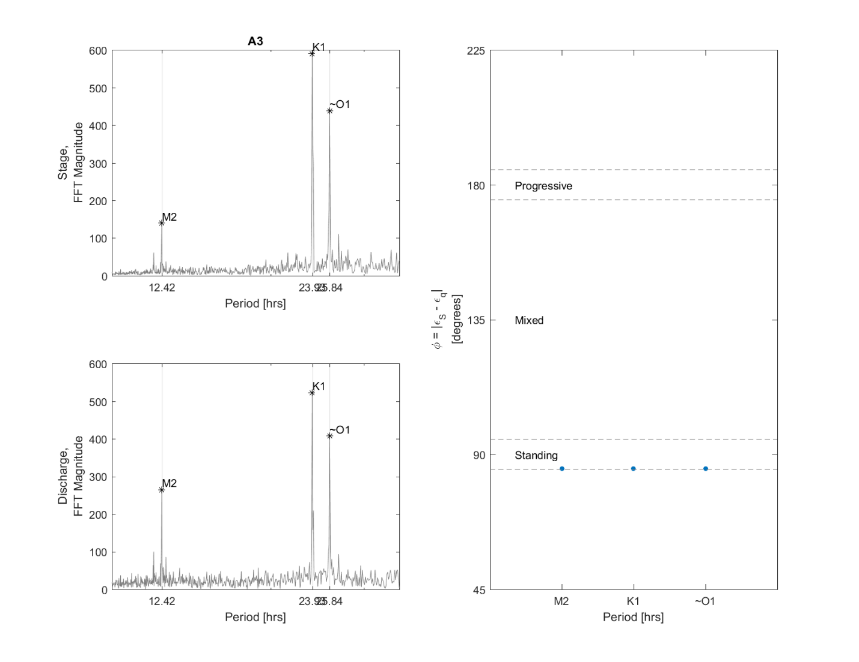


**Figure B.3**: The phase analysis for M-A site A3. The two subfigures (one atop the other) on the left-hand side of this figure, show the FFT magnitudes of the stage (left-top) and discharge (left-bottom) for the identified semidiurnal and diurnal harmonics analyzed for phase offset. The right-hand figure shows the calculated phase offset for each harmonic and compares it to the predicted offsets for a standing, mixed and progressive wave.


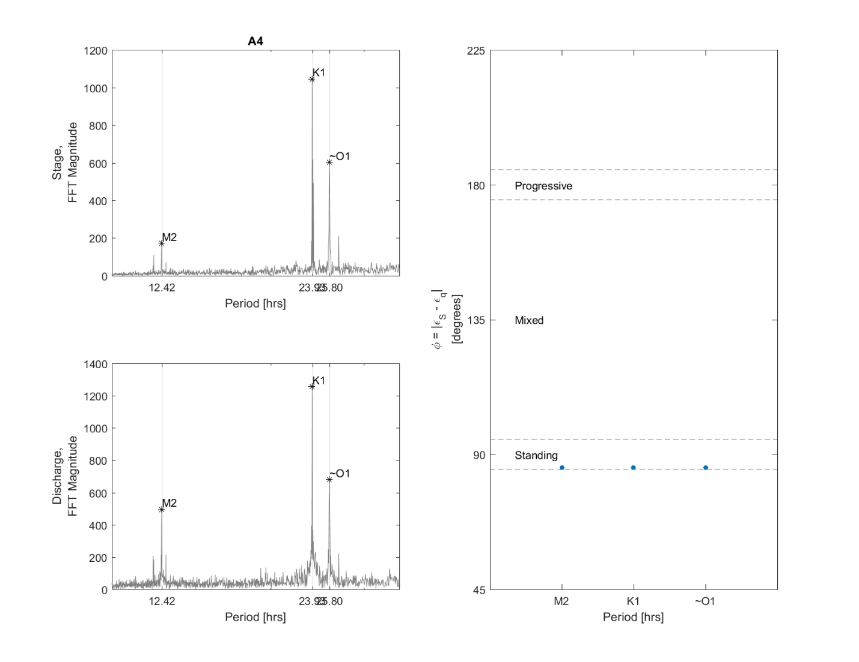


**Figure B.4**: The phase analysis for M-A site A4. The two subfigures (one atop the other) on the left-hand side of this figure, show the FFT magnitudes of the stage (left-top) and discharge (left-bottom) for the identified semidiurnal and diurnal harmonics analyzed for phase offset. The right-hand figure shows the calculated phase offset for each harmonic and compares it to the predicted offsets for a standing, mixed and progressive wave.


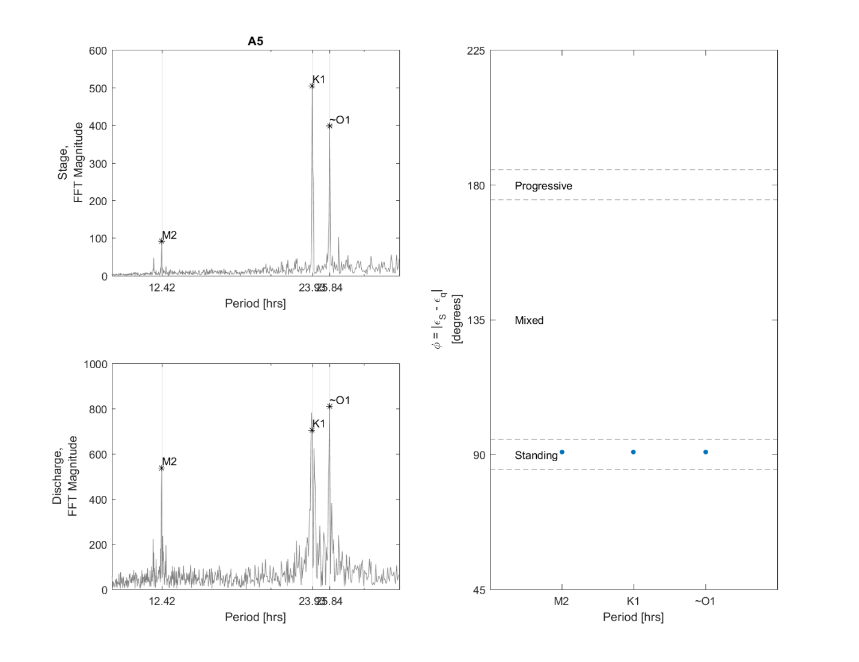


**Figure B.5**: The phase analysis for M-A site A5. The two subfigures (one atop the other) on the left-hand side of this figure, show the FFT magnitudes of the stage (left-top) and discharge (left-bottom) for the identified semidiurnal and diurnal harmonics analyzed for phase offset. The right-hand figure shows the calculated phase offset for each harmonic and compares it to the predicted offsets for a standing, mixed and progressive wave.


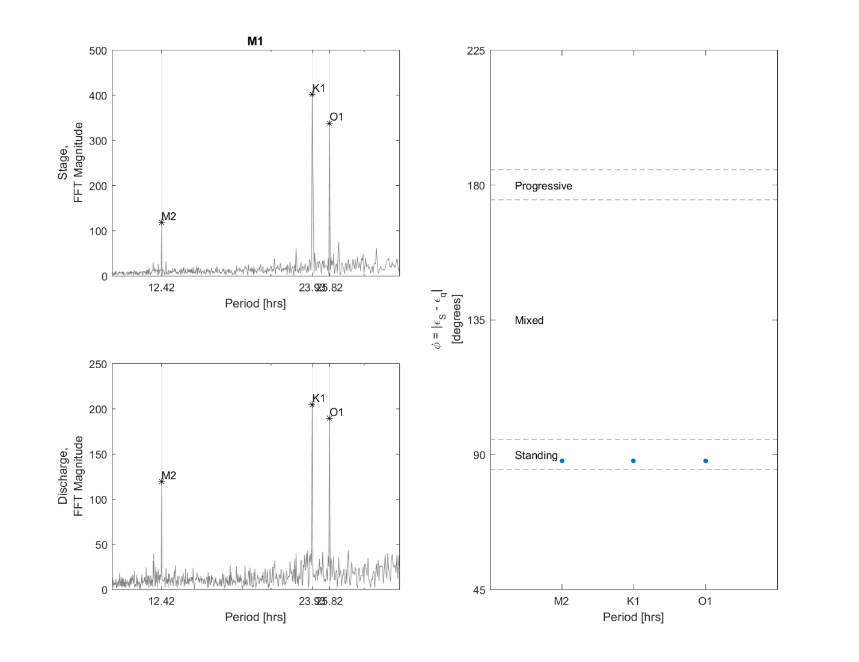


**Figure B.6**: The phase analysis for M-A site M1. The two subfigures (one atop the other) on the left-hand side of this figure, show the FFT magnitudes of the stage (left-top) and discharge (left-bottom) for the identified semidiurnal and diurnal harmonics analyzed for phase offset. The right-hand figure shows the calculated phase offset for each harmonic and compares it to the predicted offsets for a standing, mixed and progressive wave.


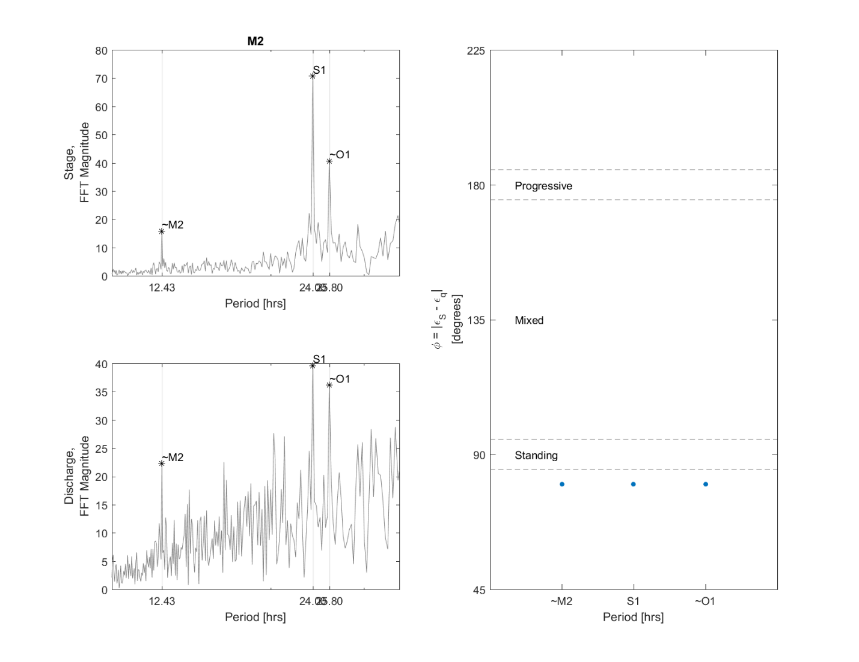


**Figure B.7**: The phase analysis for M-A site M2. The two subfigures (one atop the other) on the left-hand side of this figure, show the FFT magnitudes of the stage (left-top) and discharge (left-bottom) for the identified semidiurnal and diurnal harmonics analyzed for phase offset. The right-hand figure shows the calculated phase offset for each harmonic and compares it to the predicted offsets for a standing, mixed and progressive wave.


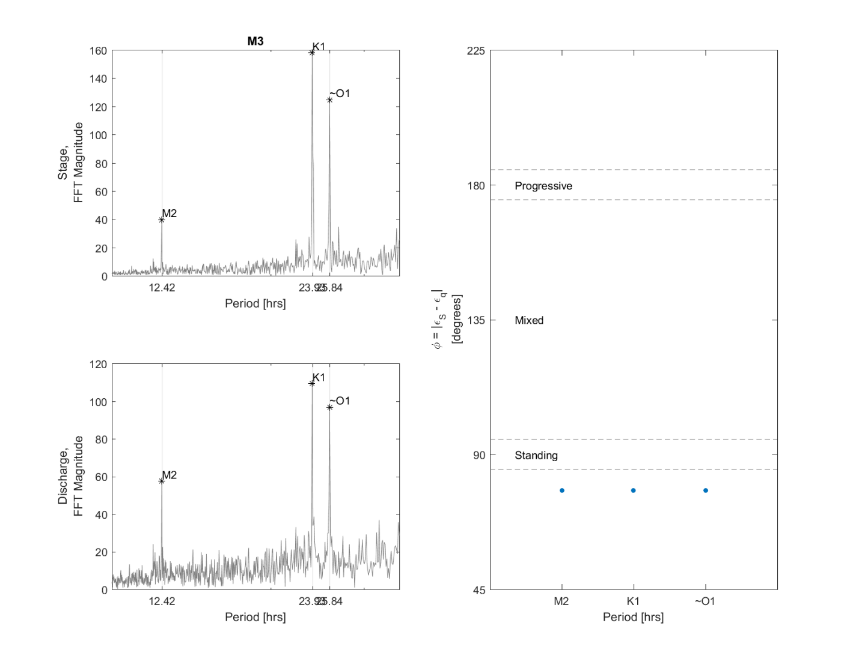


**Figure B.8**: The phase analysis for M-A site M3. The two subfigures (one atop the other) on the left-hand side of this figure, show the FFT magnitudes of the stage (left-top) and discharge (left-bottom) for the identified semidiurnal and diurnal harmonics analyzed for phase offset. The right-hand figure shows the calculated phase offset for each harmonic and compares it to the predicted offsets for a standing, mixed and progressive wave.


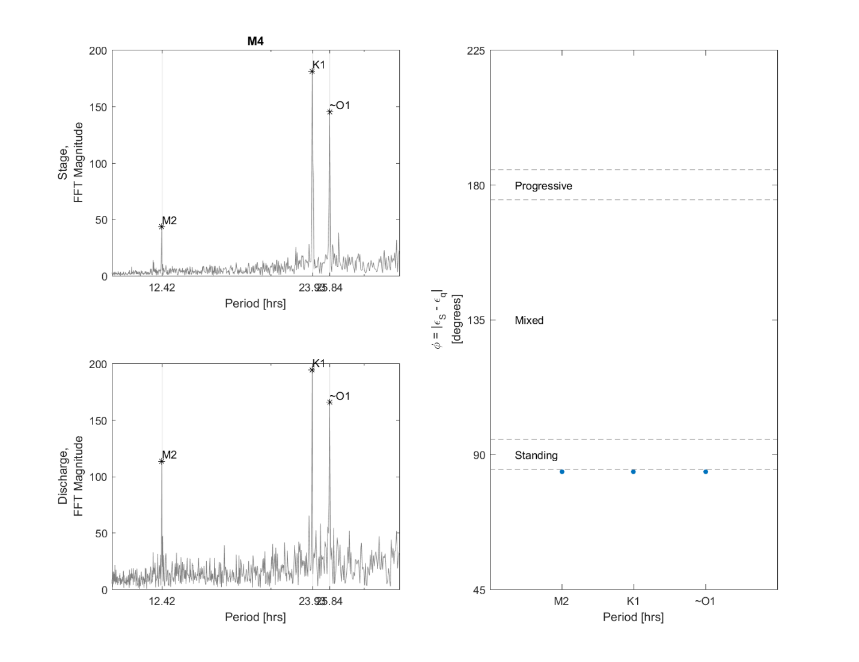


**Figure B.9**: The phase analysis for M-A site M4. The two subfigures (one atop the other) on the left-hand side of this figure, show the FFT magnitudes of the stage (left-top) and discharge (left-bottom) for the identified semidiurnal and diurnal harmonics analyzed for phase offset. The right-hand figure shows the calculated phase offset for each harmonic and compares it to the predicted offsets for a standing, mixed and progressive wave.


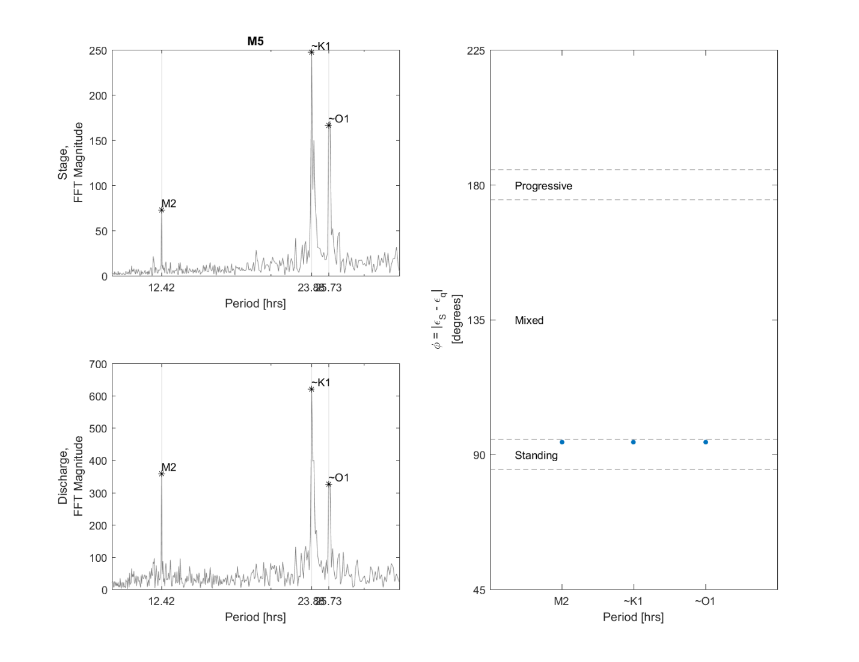


**Figure B.10**: The phase analysis for M-A site M5. The two subfigures (one atop the other) on the left-hand side of this figure, show the FFT magnitudes of the stage (left-top) and discharge (left-bottom) for the identified semidiurnal and diurnal harmonics analyzed for phase offset. The right-hand figure shows the calculated phase offset for each harmonic and compares it to the predicted offsets for a standing, mixed and progressive wave.


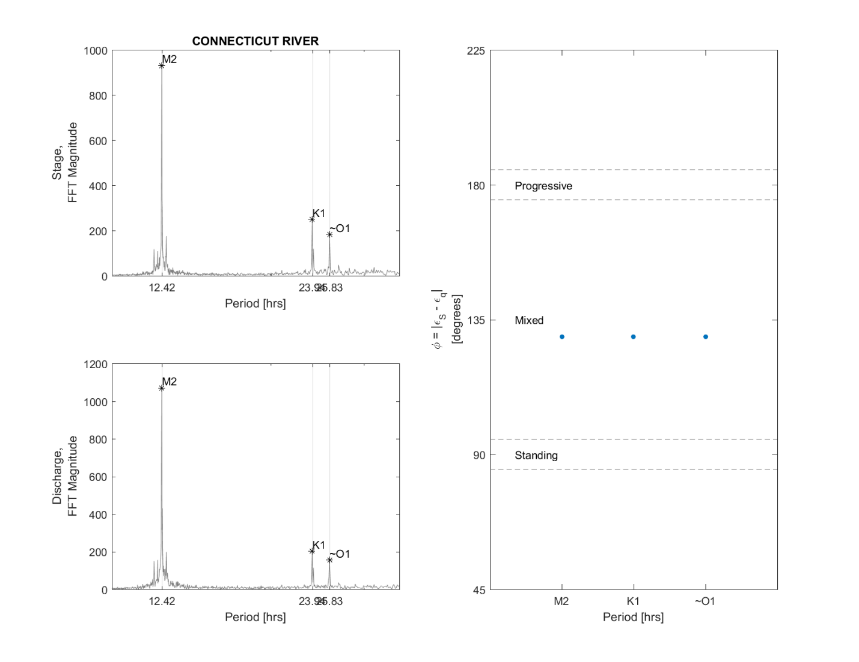


**Figure B.11**: The phase analysis for USGS site on the Connecticut River [site number: 01193050]. The two subfigures (one atop the other) on the left-hand side of this figure, show the FFT magnitudes of the stage (left-top) and discharge (left-bottom) for the identified semidiurnal and diurnal harmonics analyzed for phase offset. The right-hand figure shows the calculated phase offset for each harmonic and compares it to the predicted offsets for a standing, mixed and progressive wave.


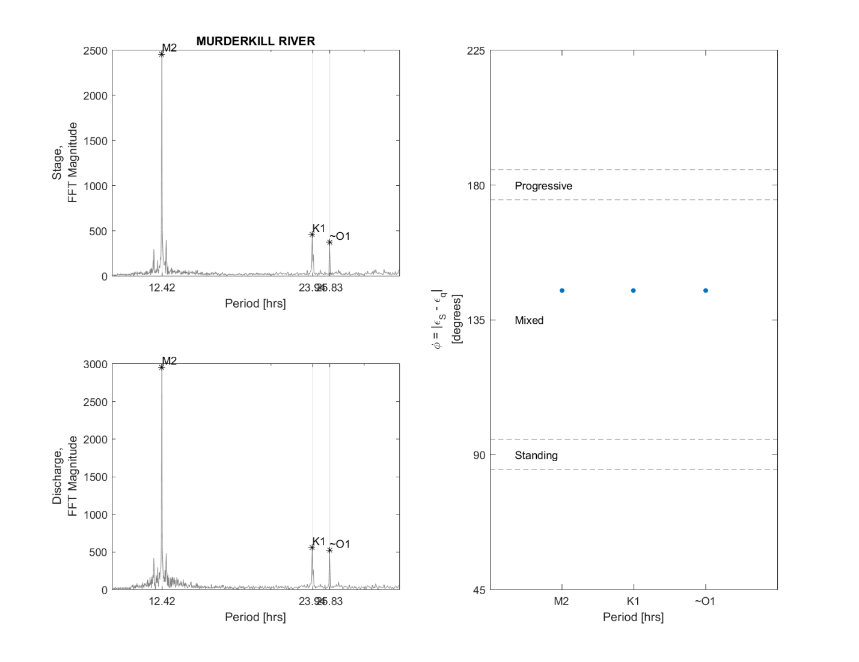


**Figure B.12**: The phase analysis for USGS site on the Murderkill River [site number: 01484085]. The two subfigures (one atop the other) on the left-hand side of this figure, show the FFT magnitudes of the stage (left-top) and discharge (left-bottom) for the identified semidiurnal and diurnal harmonics analyzed for phase offset. The right-hand figure shows the calculated phase offset for each harmonic and compares it to the predicted offsets for a standing, mixed and progressive wave.


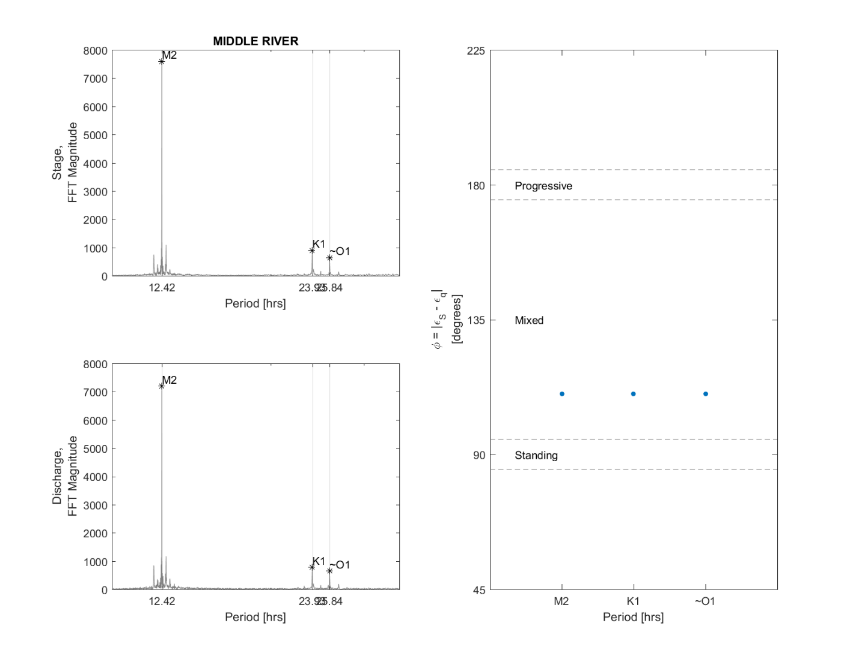


**Figure B.13:** The phase analysis for USGS site on the Middle River [site number: 02198950]. The two subfigures (one atop the other) on the left-hand side of this figure, show the FFT magnitudes of the stage (left-top) and discharge (left-bottom) for the identified semidiurnal and diurnal harmonics analyzed for phase offset. The right-hand figure shows the calculated phase offset for each harmonic and compares it to the predicted offsets for a standing, mixed and progressive wave.


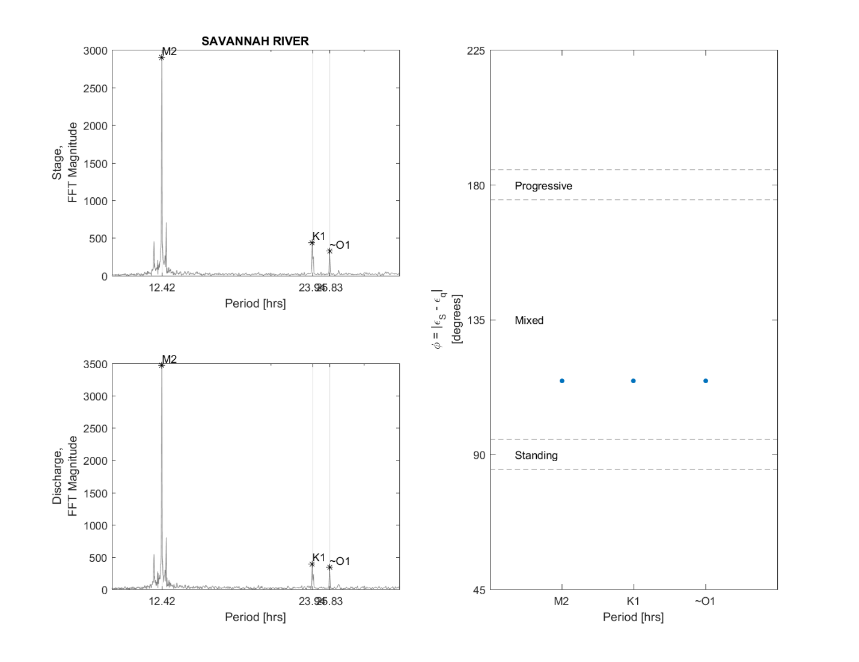


**Figure B.14**: The phase analysis for USGS site on the Savannah River [site number: 02198980]. The two subfigures (one atop the other) on the left-hand side of this figure, show the FFT magnitudes of the stage (left-top) and discharge (left-bottom) for the identified semidiurnal and diurnal harmonics analyzed for phase offset. The right-hand figure shows the calculated phase offset for each harmonic and compares it to the predicted offsets for a standing, mixed and progressive wave.


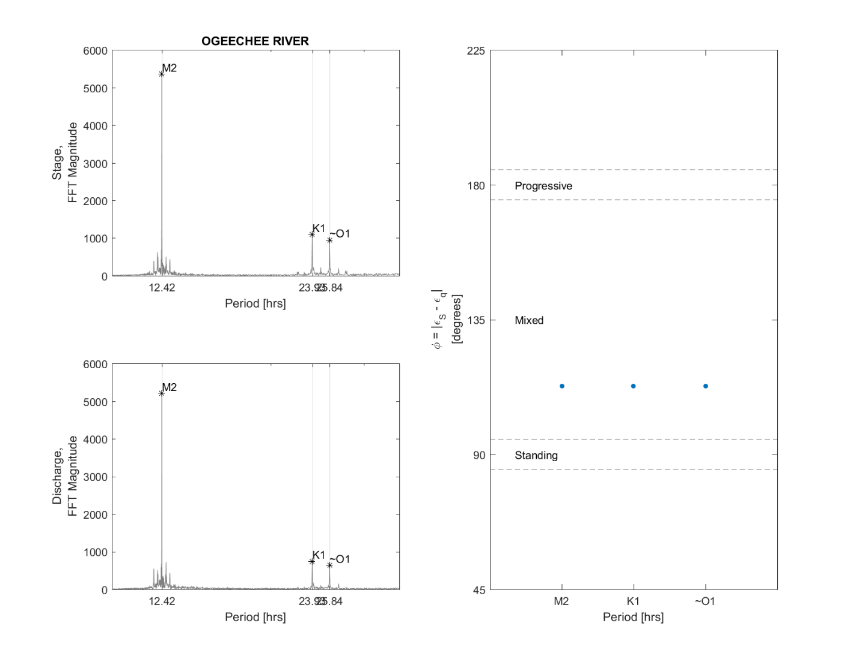


**Figure B.15**: The phase analysis for USGS site on the Ogeechee River [site number: 02203536]. The two subfigures (one atop the other) on the left-hand side of this figure, show the FFT magnitudes of the stage (left-top) and discharge (left-bottom) for the identified semidiurnal and diurnal harmonics analyzed for phase offset. The right-hand figure shows the calculated phase offset for each harmonic and compares it to the predicted offsets for a standing, mixed and progressive wave.


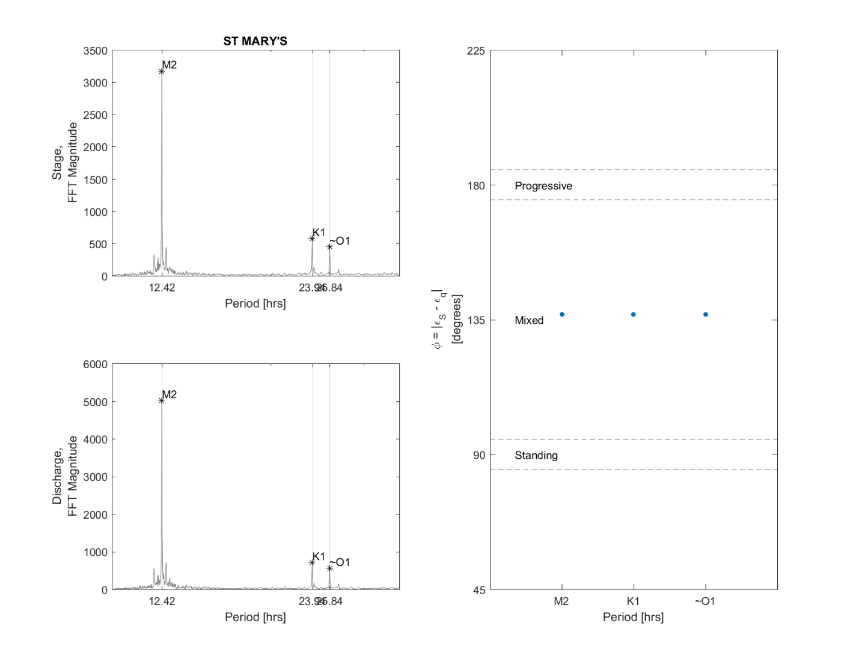


**Figure B.16**: The phase analysis for USGS site on the St. Mary’s River [site number: 02231254]. The two subfigures (one atop the other) on the left-hand side of this figure, show the FFT magnitudes of the stage (left-top) and discharge (left-bottom) for the identified semidiurnal and diurnal harmonics analyzed for phase offset. The right-hand figure shows the calculated phase offset for each harmonic and compares it to the predicted offsets for a standing, mixed and progressive wave.


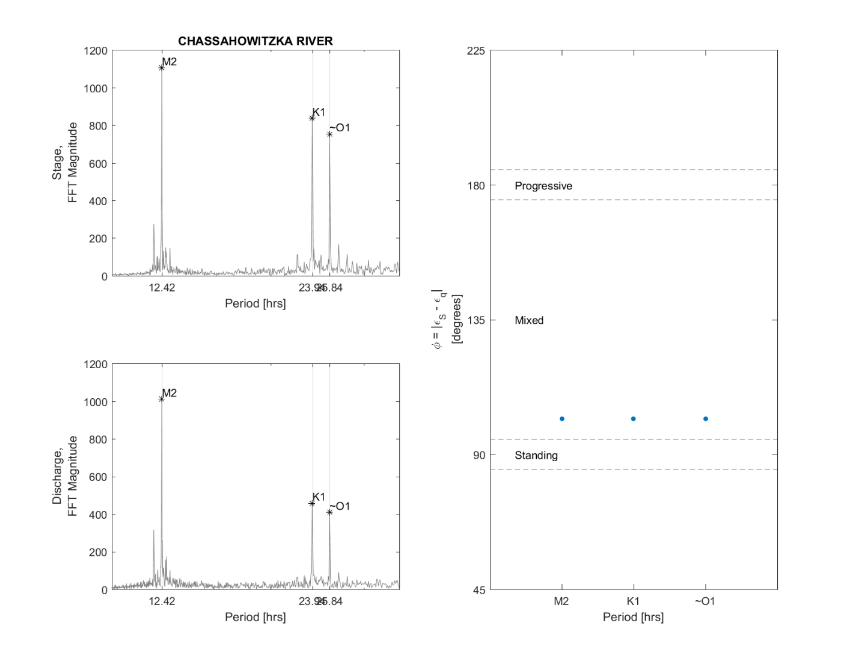


**Figure B.17**: The phase analysis for USGS site on the Chassahowitzka River [site number: 02310663]. The two subfigures (one atop the other) on the left-hand side of this figure, show the FFT magnitudes of the stage (left-top) and discharge (left-bottom) for the identified semidiurnal and diurnal harmonics analyzed for phase offset. The right-hand figure shows the calculated phase offset for each harmonic and compares it to the predicted offsets for a standing, mixed and progressive wave.


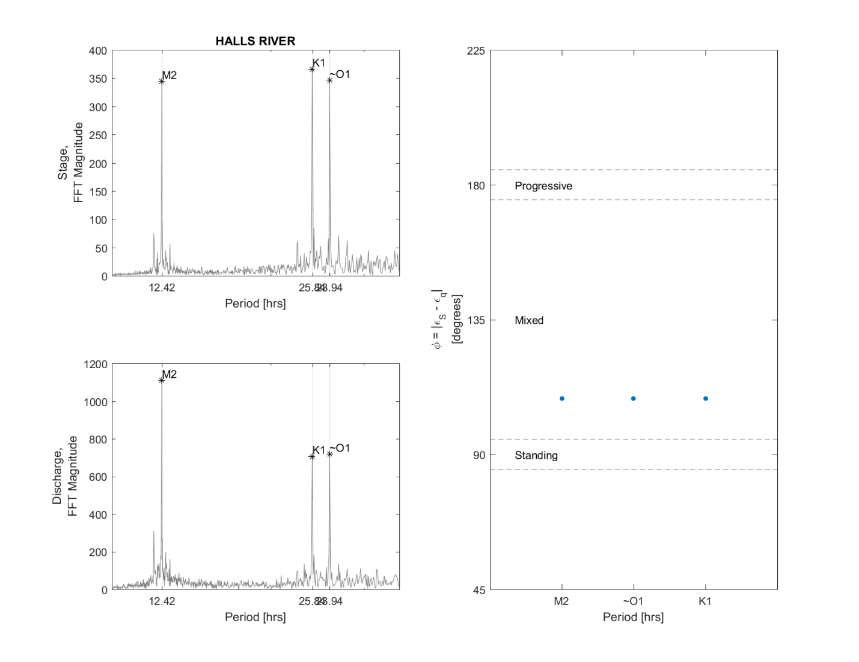


**Figure B.18**: The phase analysis for USGS site on the Halls River [site number: 02310689]. The two subfigures (one atop the other) on the left-hand side of this figure, show the FFT magnitudes of the stage (left-top) and discharge (left-bottom) for the identified semidiurnal and diurnal harmonics analyzed for phase offset. The right-hand figure shows the calculated phase offset for each harmonic and compares it to the predicted offsets for a standing, mixed and progressive wave.


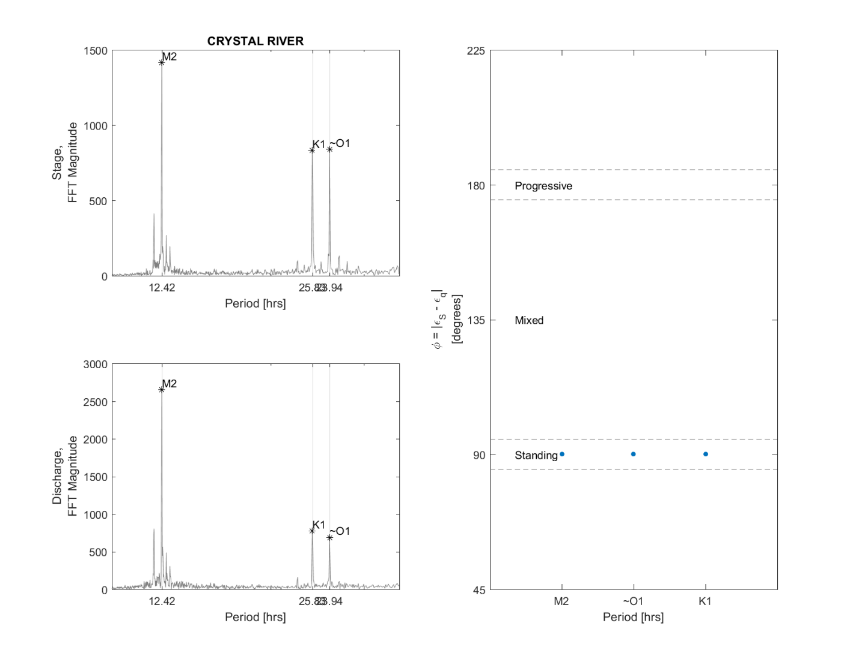


**Figure B.19**: The phase analysis for USGS site on the Crystal River [site number: 02310747]. The two subfigures (one atop the other) on the left-hand side of this figure, show the FFT magnitudes of the stage (left-top) and discharge (left-bottom) for the identified semidiurnal and diurnal harmonics analyzed for phase offset. The right-hand figure shows the calculated phase offset for each harmonic and compares it to the predicted offsets for a standing, mixed and progressive wave.


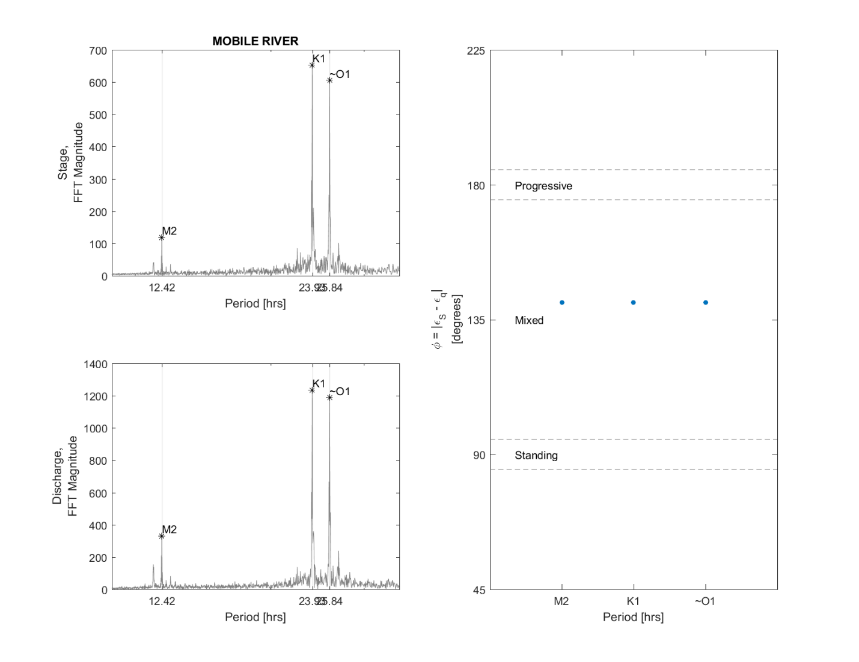


**Figure B.20**: The phase analysis for USGS site on the Mobile River [site number: 02470629]. The two subfigures (one atop the other) on the left-hand side of this figure, show the FFT magnitudes of the stage (left-top) and discharge (left-bottom) for the identified semidiurnal and diurnal harmonics analyzed for phase offset. The right-hand figure shows the calculated phase offset for each harmonic and compares it to the predicted offsets for a standing, mixed and progressive wave.


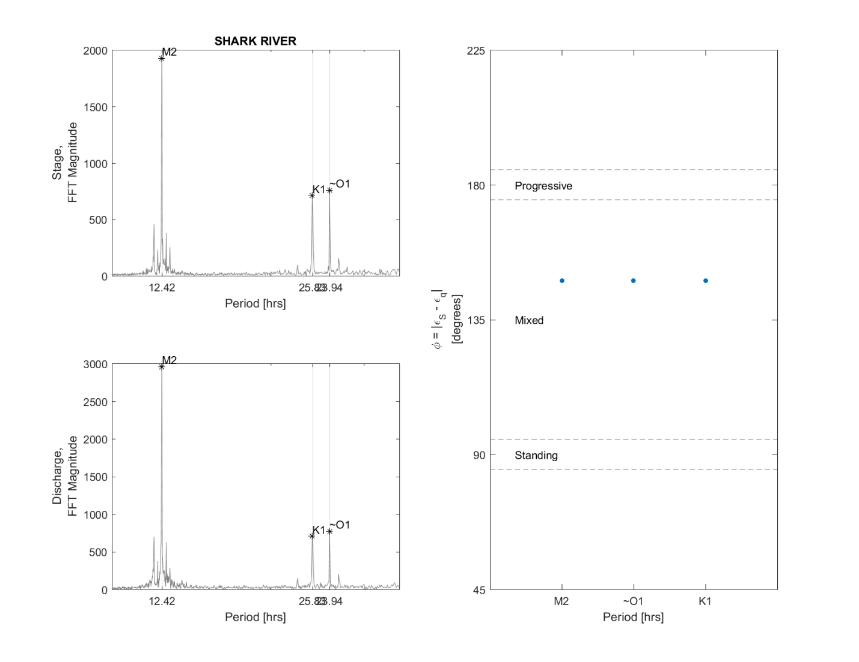


**Figure B.21**: The phase analysis for USGS site on the Shark River [site number: 252230081021300]. The two subfigures (one atop the other) on the left-hand side of this figure, show the FFT magnitudes of the stage (left-top) and discharge (left-bottom) for the identified semidiurnal and diurnal harmonics analyzed for phase offset. The right-hand figure shows the calculated phase offset for each harmonic and compares it to the predicted offsets for a standing, mixed and progressive wave.


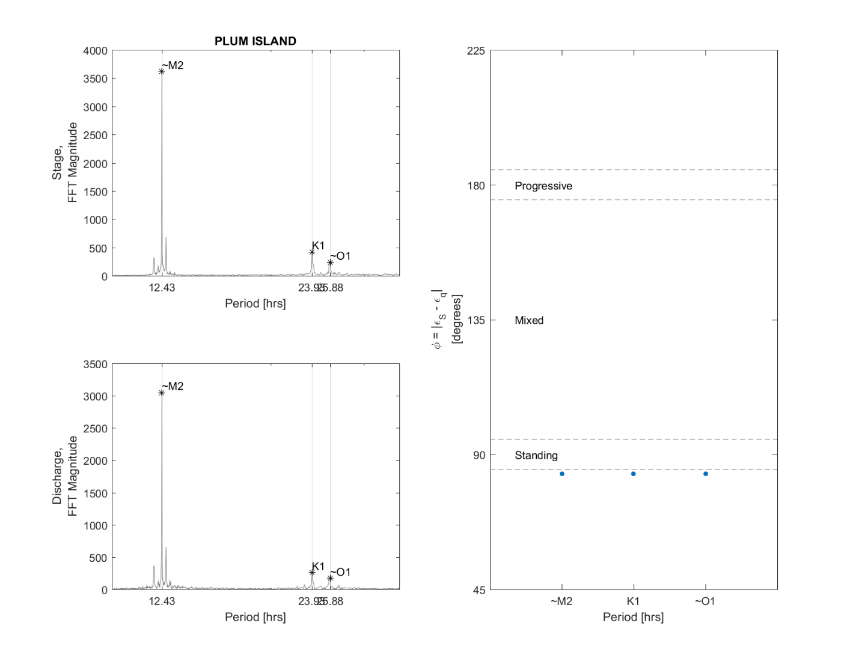


**Figure B.22** The phase analysis for USGS site on the Plum Island River [site number: 424752070491701]. The two subfigures (one atop the other) on the left-hand side of this figure, show the FFT magnitudes of the stage (left-top) and discharge (left-bottom) for the identified semidiurnal and diurnal harmonics analyzed for phase offset. The right-hand figure shows the calculated phase offset for each harmonic and compares it to the predicted offsets for a standing, mixed and progressive wave.

# C. Summary of baseflow regressions

The following figures are provide a visual representation of the best linear model of equation (3) fit to each M-A site and the twelve USGS sites. The exact value of the coefficients, k_1_-k_4_, are listed in Table 2.


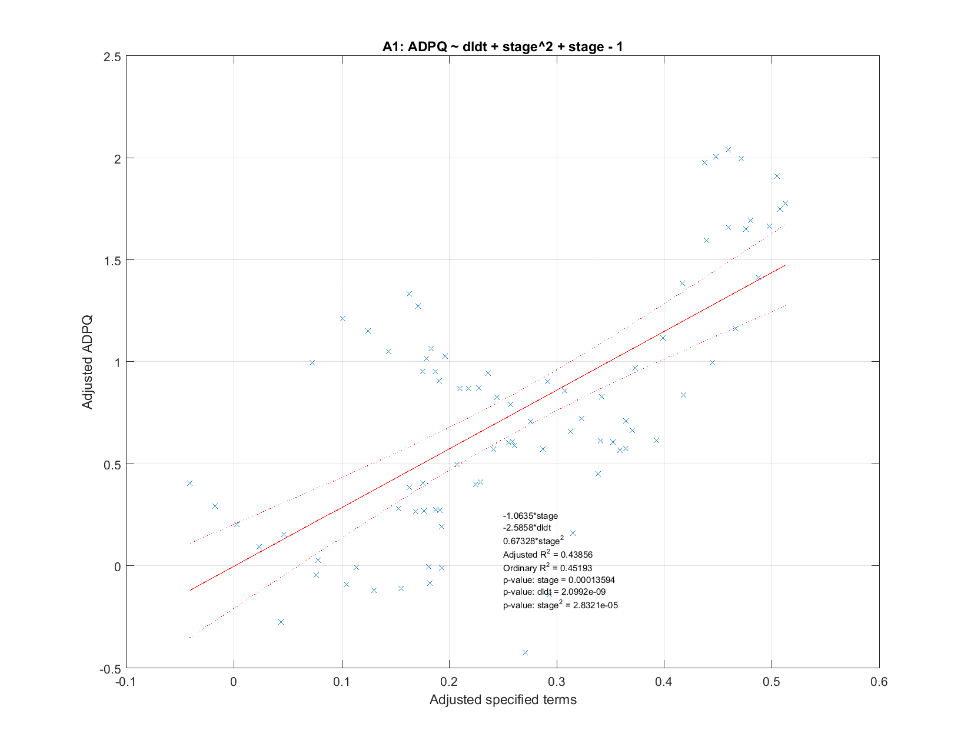


**Figure C.1**: The linear regression data for site A1.


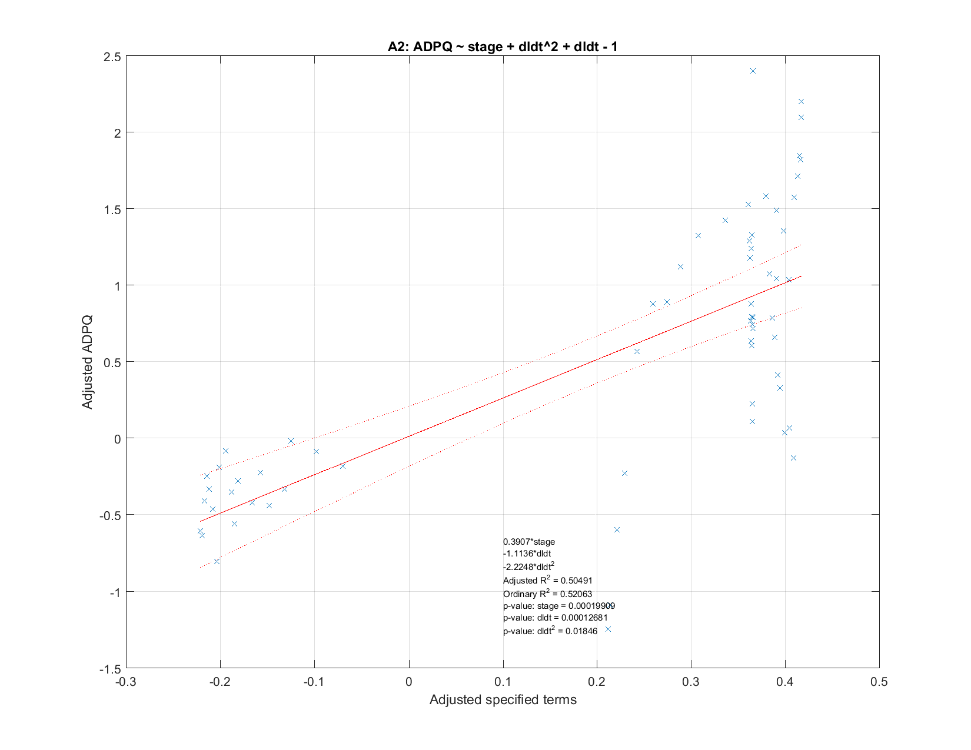


**Figure C.2**: The linear regression data for site A2.


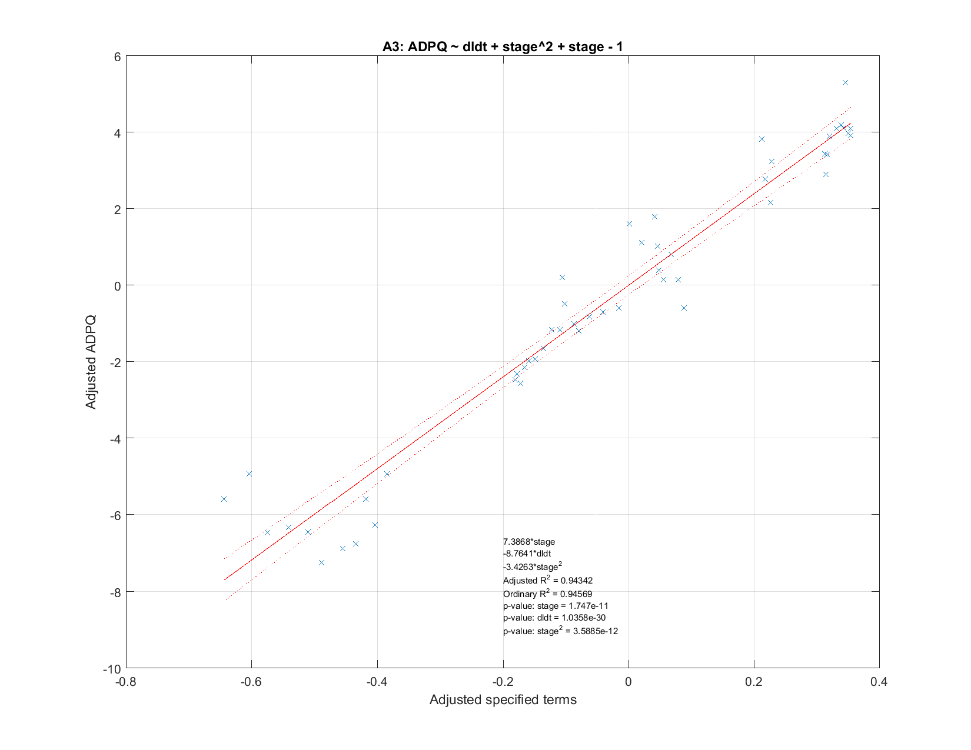


**Figure C.3**: The linear regression data for site A3.

**
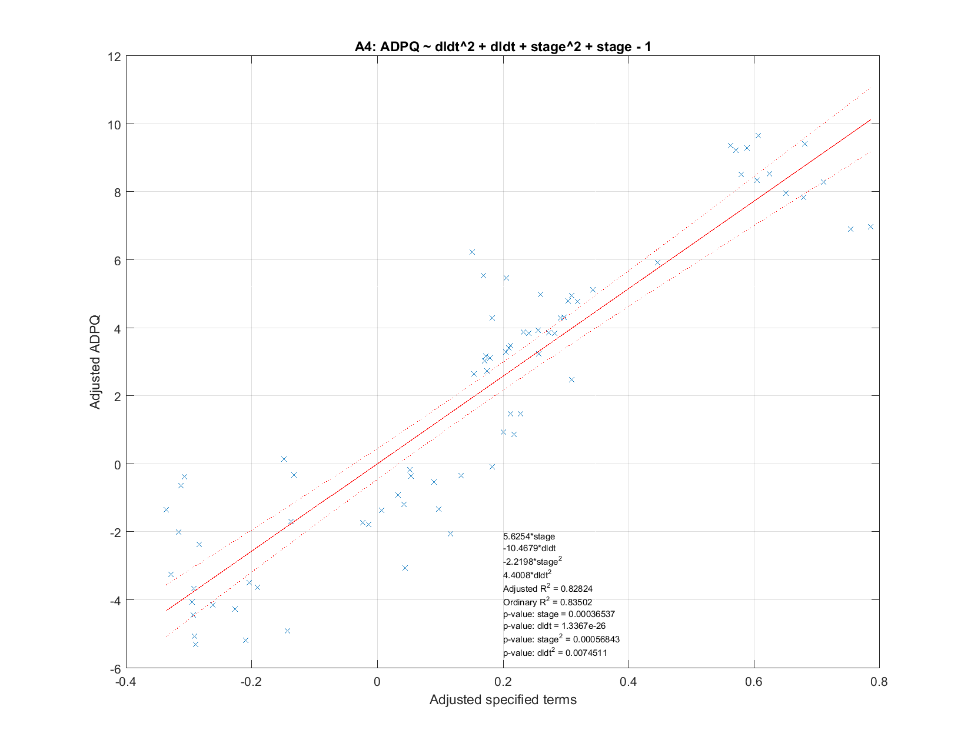
**

**Figure C.4**: The linear regression data for site A4.


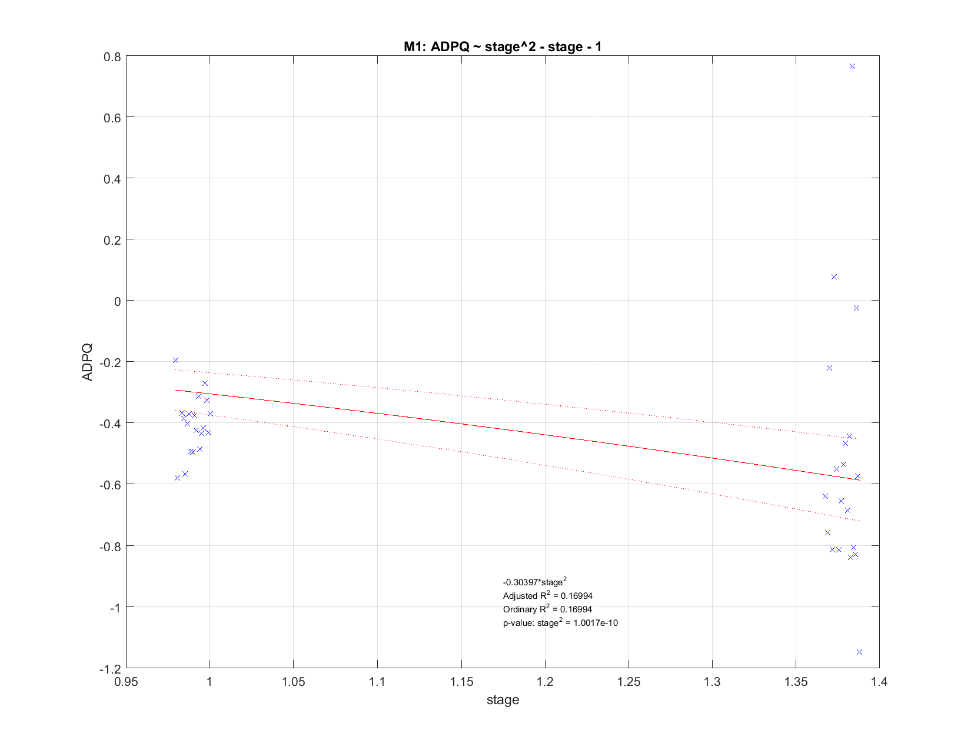


**Figure C.5**: The linear regression data for site M1.


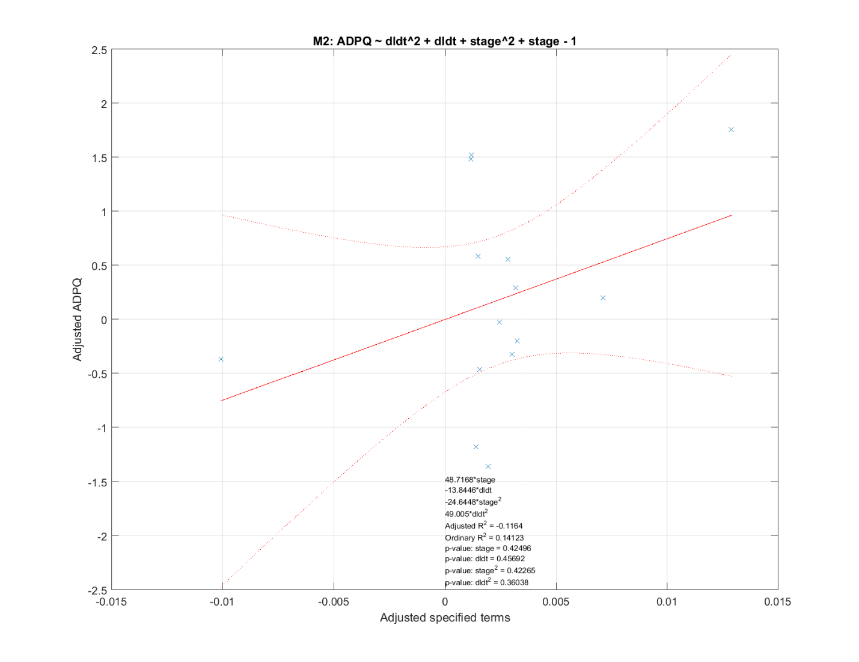


**Figure C.6**: The linear regression data for site M2. Because this linear regression produced an r^2^ less than 0, these results were not included in the manuscript. The poor nature of this fit is likely due to limited ADP observations and installation data. Evidence suggests that our installation was stolen.


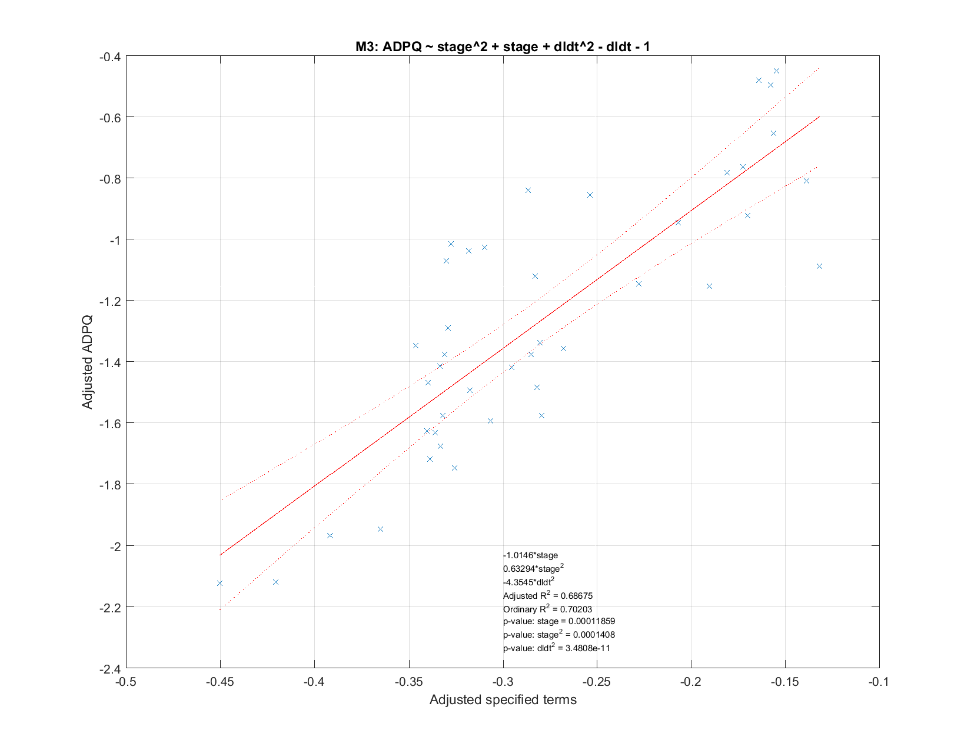


**Figure C.7**: The linear regression data for site M3.

**
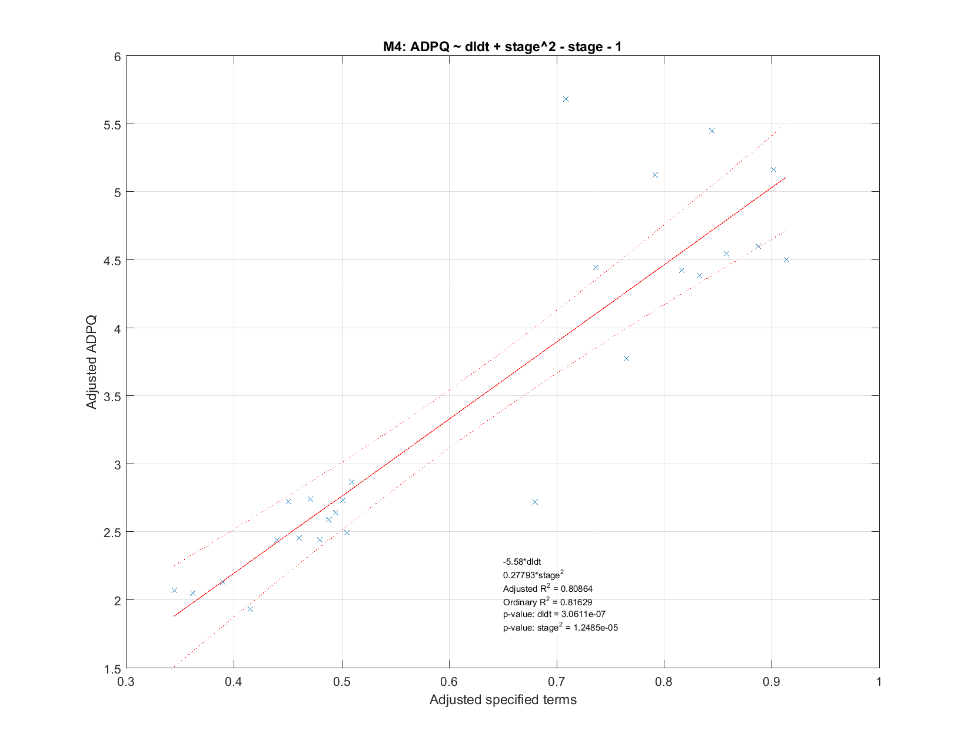
**

**Figure C.8**: The linear regression data for site M4.

**
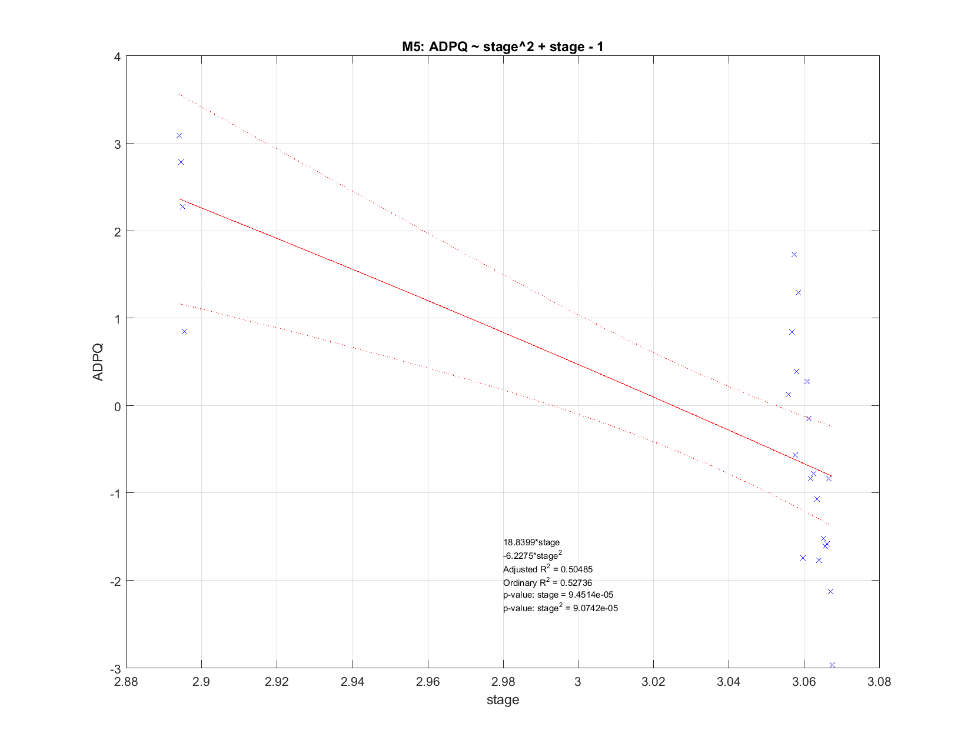
**

**Figure C.9**: The linear regression data for site M5.


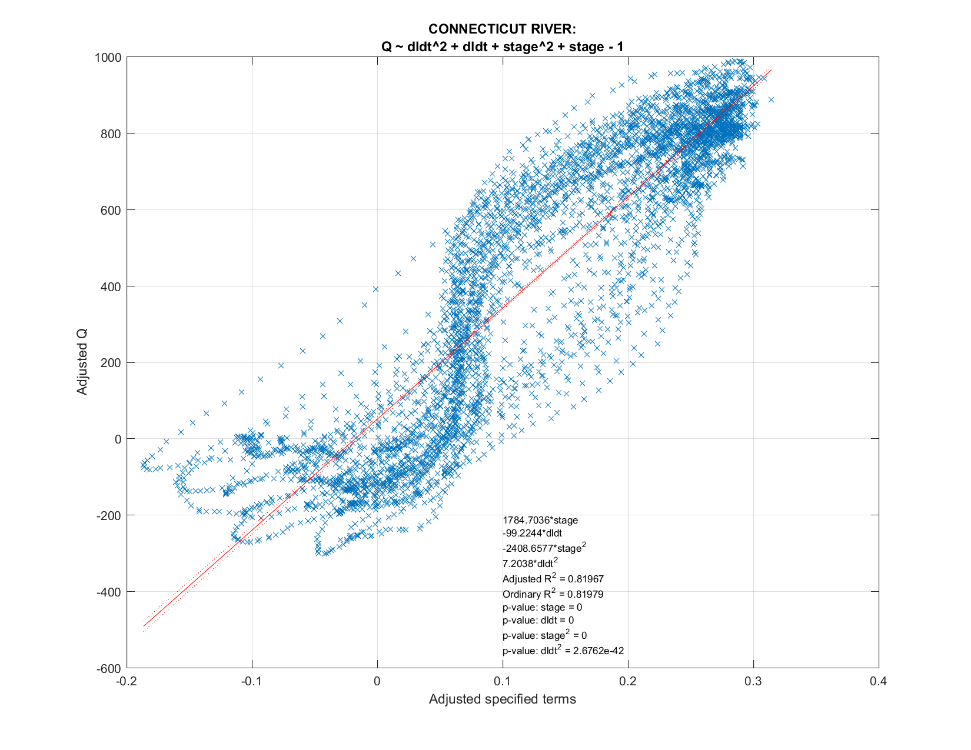


**Figure C.10**: The linear regression data for USGS site on the Connecticut River [site number: 01193050].


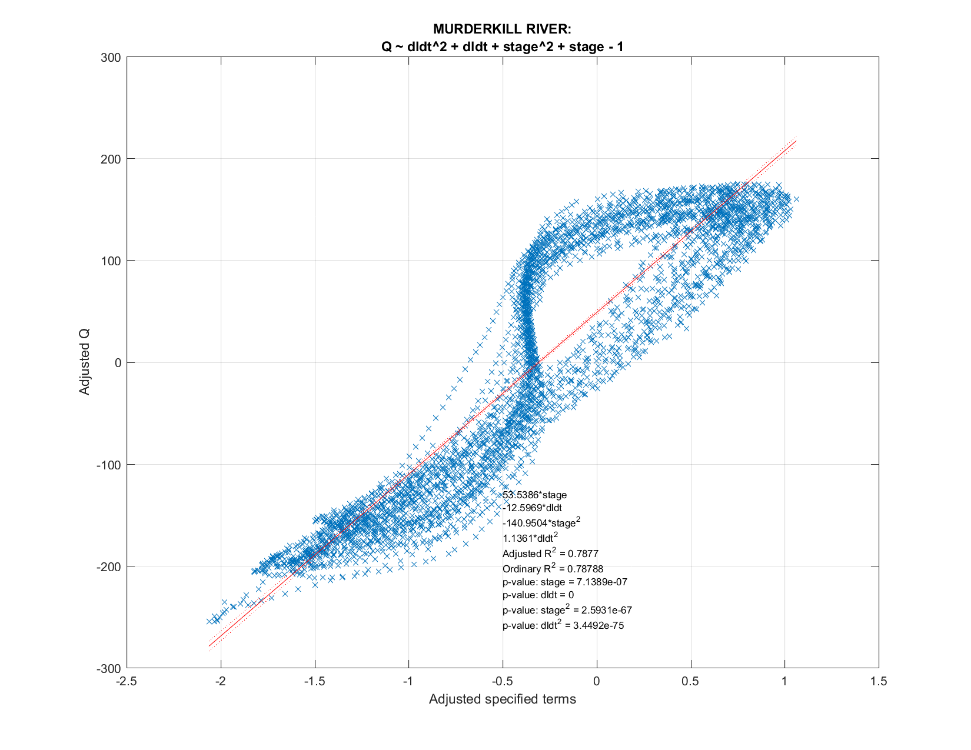


**Figure C.11**: The linear regression data for USGS site on the Murderkill River [site number: 01484085].


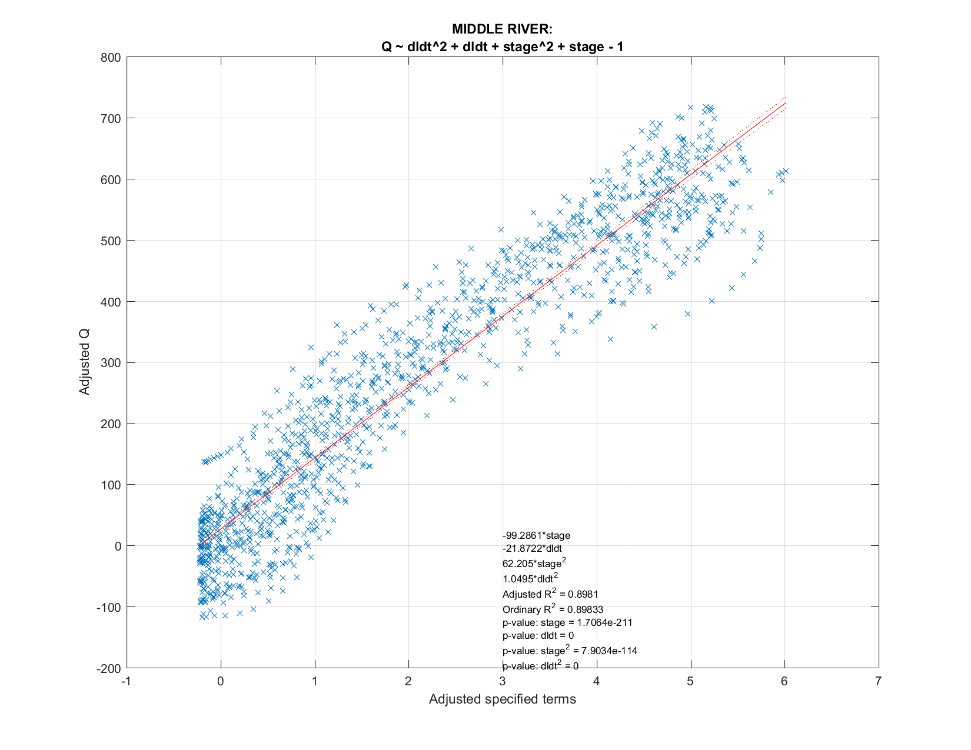


**Figure C.12**: The linear regression data for USGS site on the Middle River [site number: 02198950].


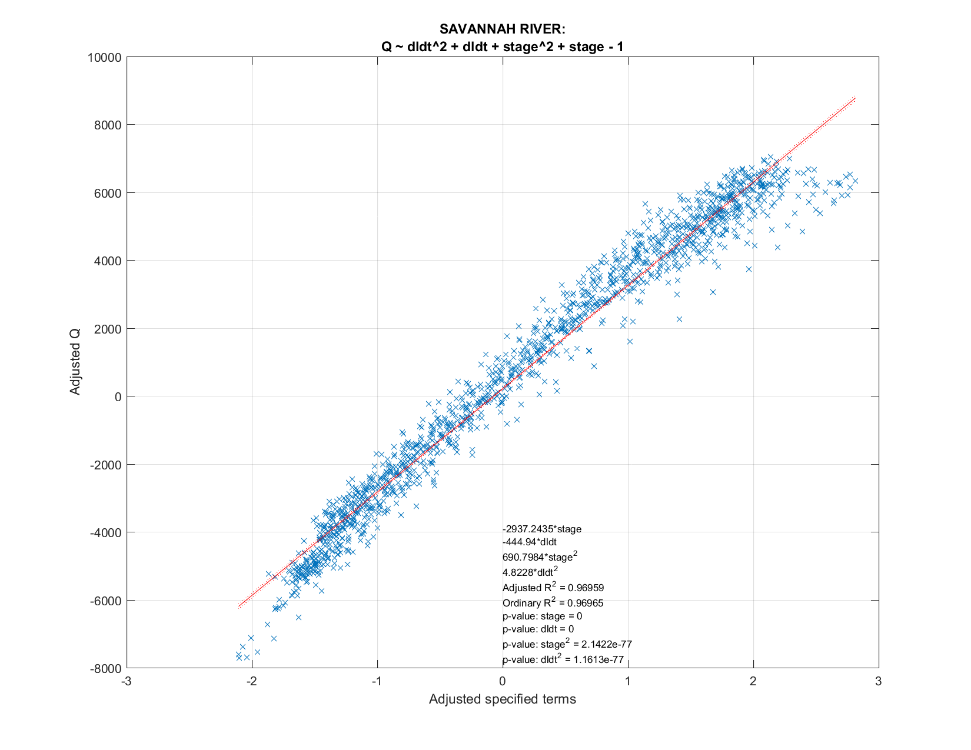


**Figure C.13**: The linear regression data for USGS site on the Savannah River [site number: 02198980].


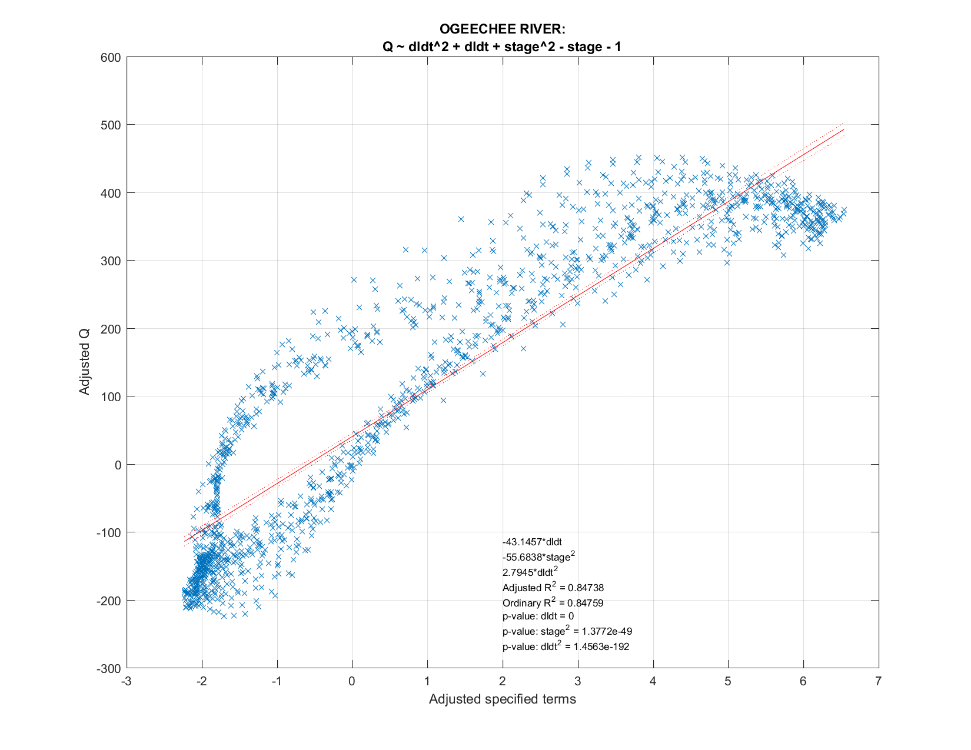


**Figure C.14**: The linear regression data for USGS site on the Ogeechee River [site number: 02203536].


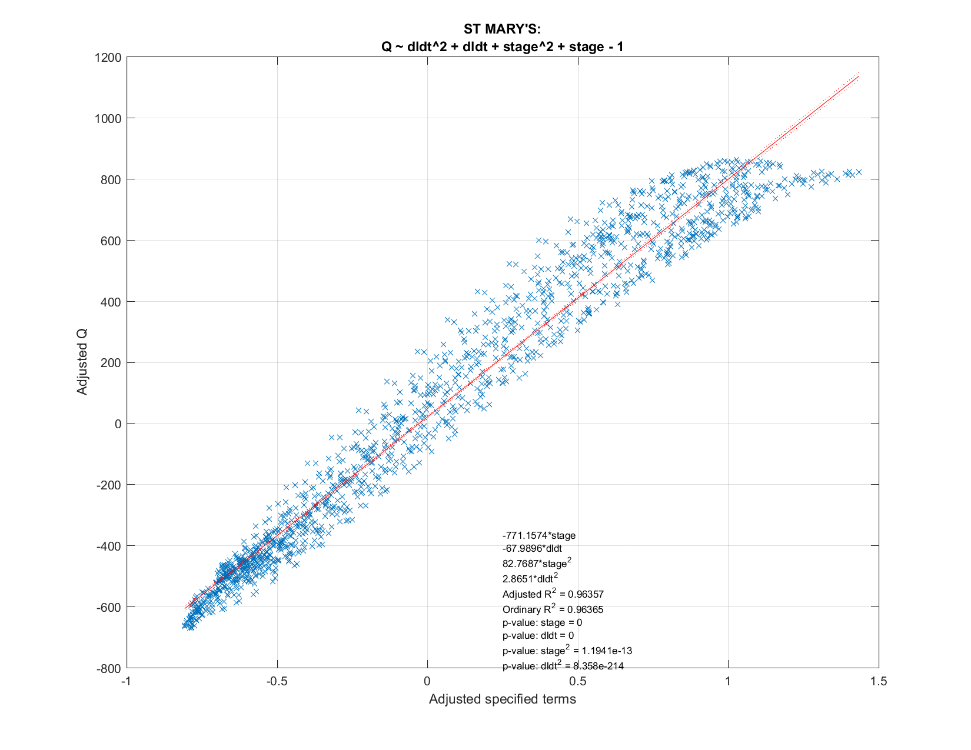


**Figure C.15**: The linear regression data for USGS site on the St. Mary’s River [site number: 02231254].


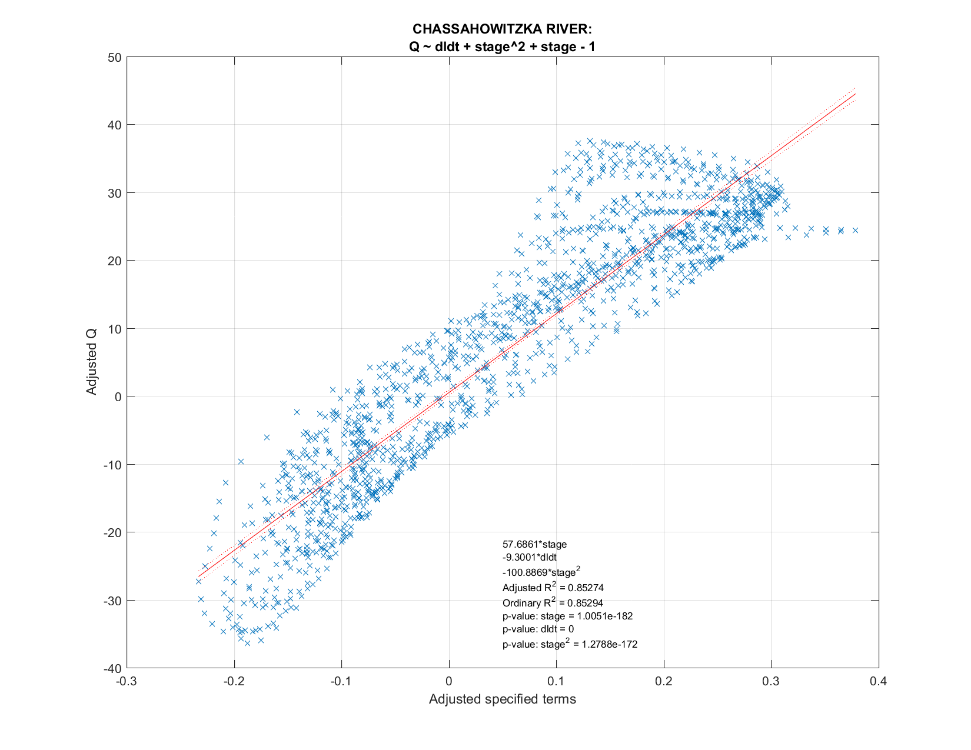


**Figure C.16**: The linear regression data for USGS site on the Chassahowitzka River [site number: 02310663].


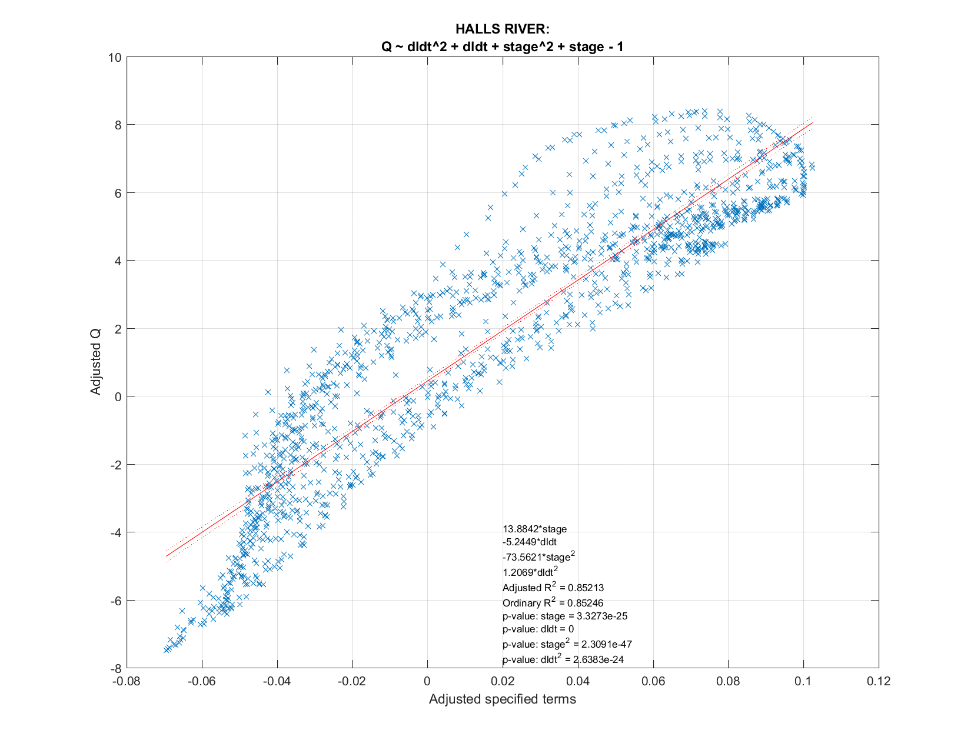


**Figure C.17**: The linear regression data for USGS site on the Halls River [site number: 02310689].


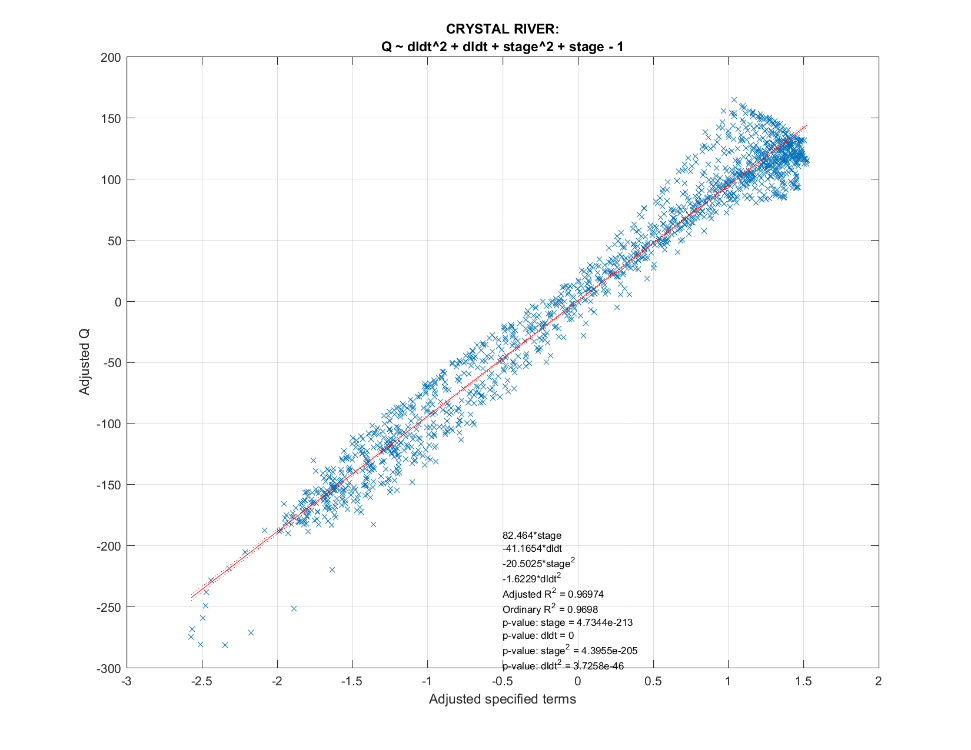


**Figure C.18**: The linear regression data for USGS site on the Crystal River [site number: 02310747].

**
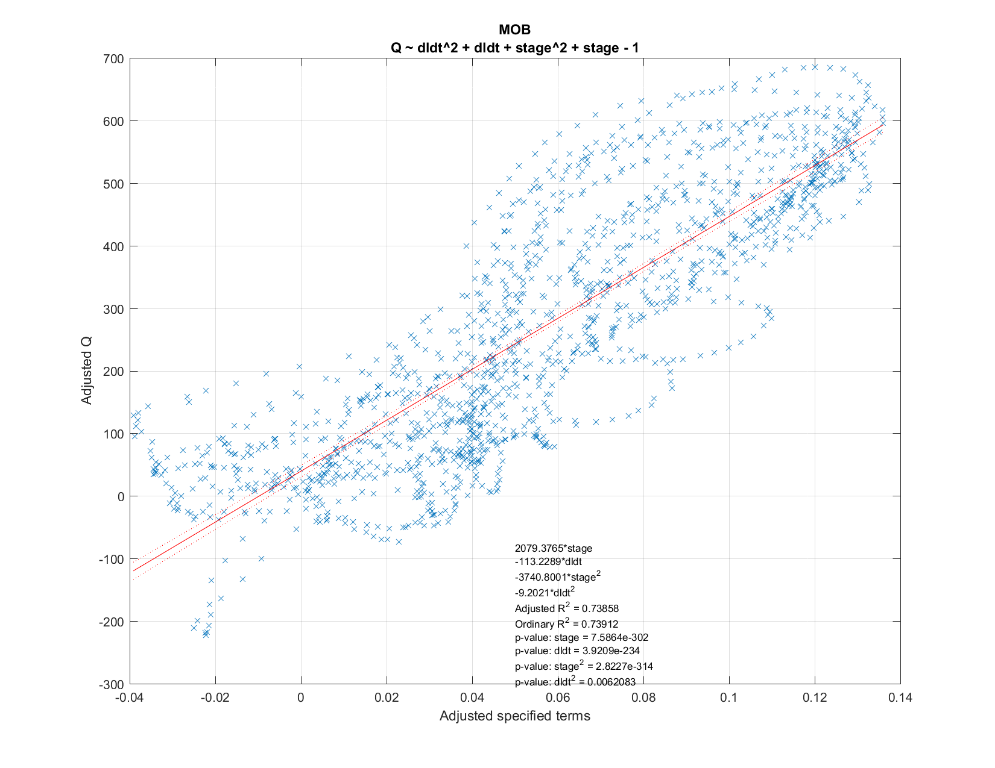
**

**Figure C.19**: The linear regression data for USGS site on the Mobile River [site number: 02470629].


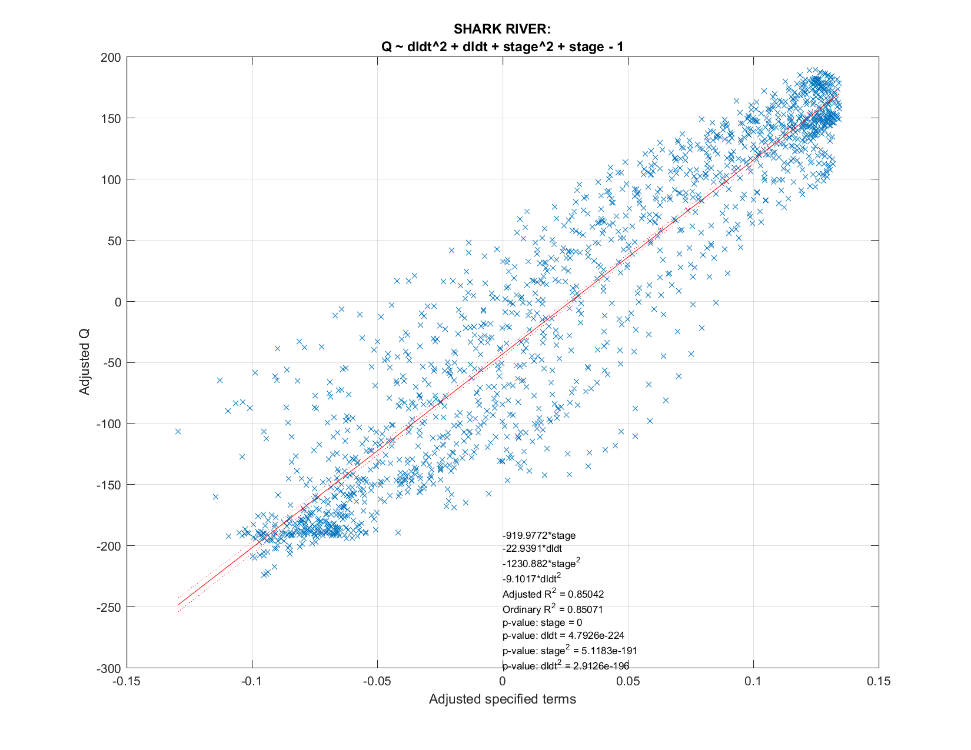


**Figure C.20**: The linear regression data for USGS site on the Shark River [site number: 252230081021300].


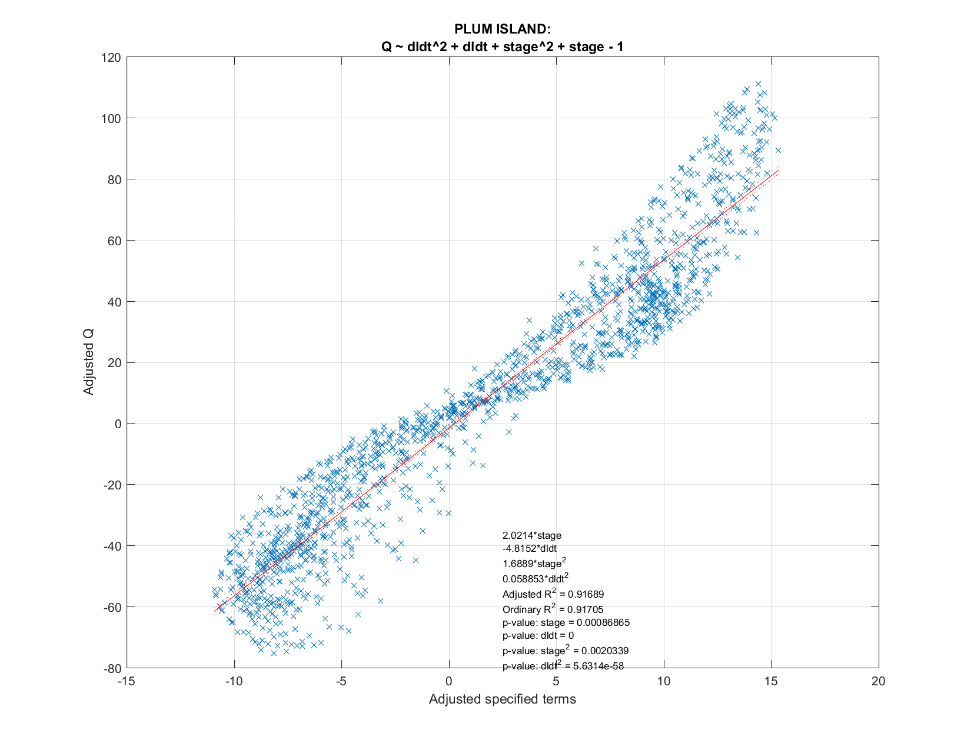


**Figure C.21**: The linear regression data for USGS site on the Shark River [site number: 424752070491701].

# D. M-A discharge time series

The following are the calculated discharge time series for each M-A site.


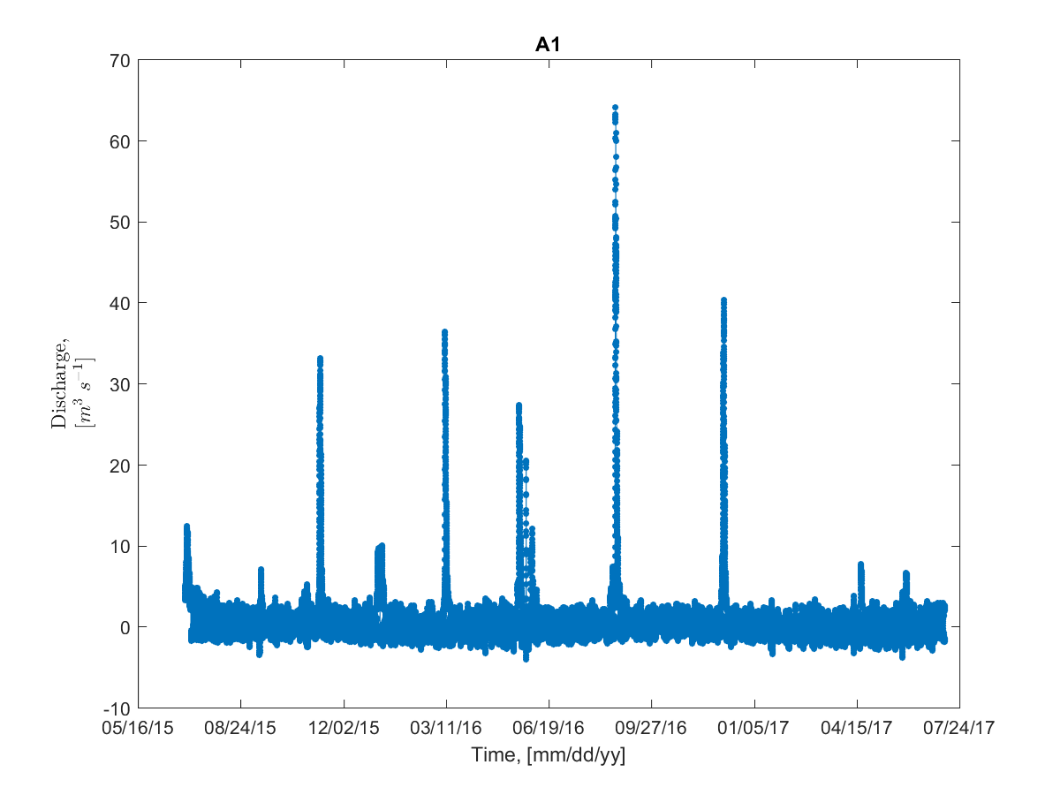


**Figure D.1**: The calculated discharge time series for M-A site: A1.


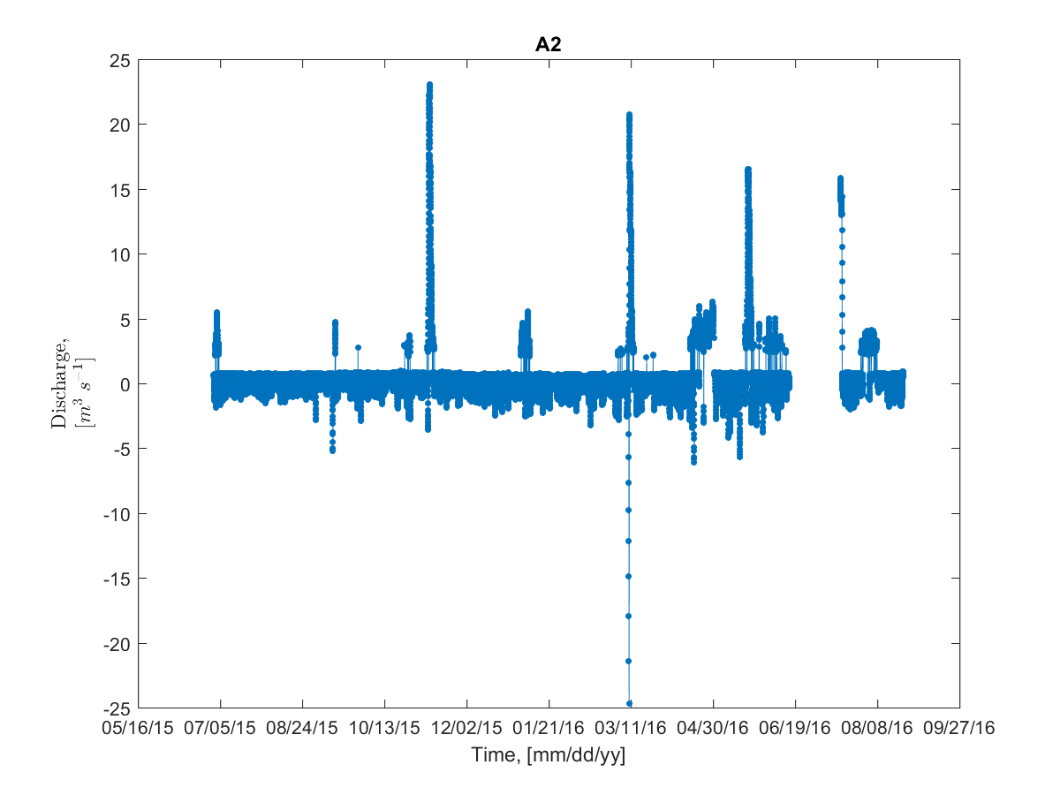


**Figure D.2**: The calculated discharge time series for M-A site: A2.


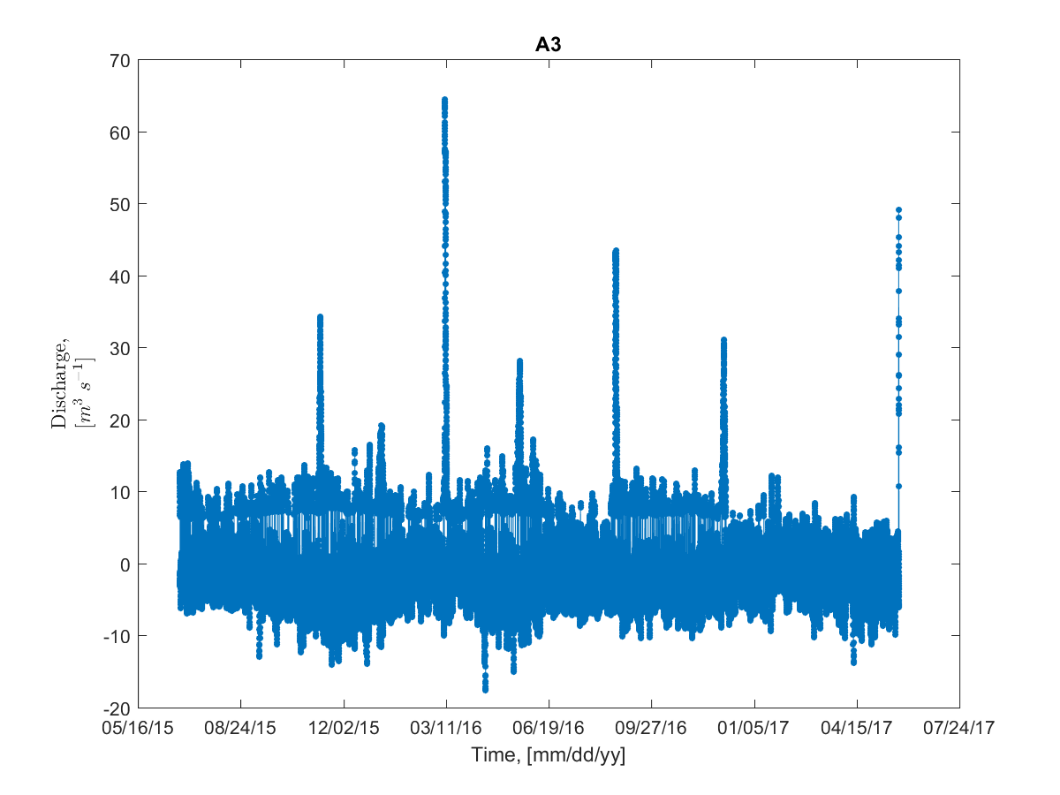


**Figure D.3**: The calculated discharge time series for M-A site: A3.


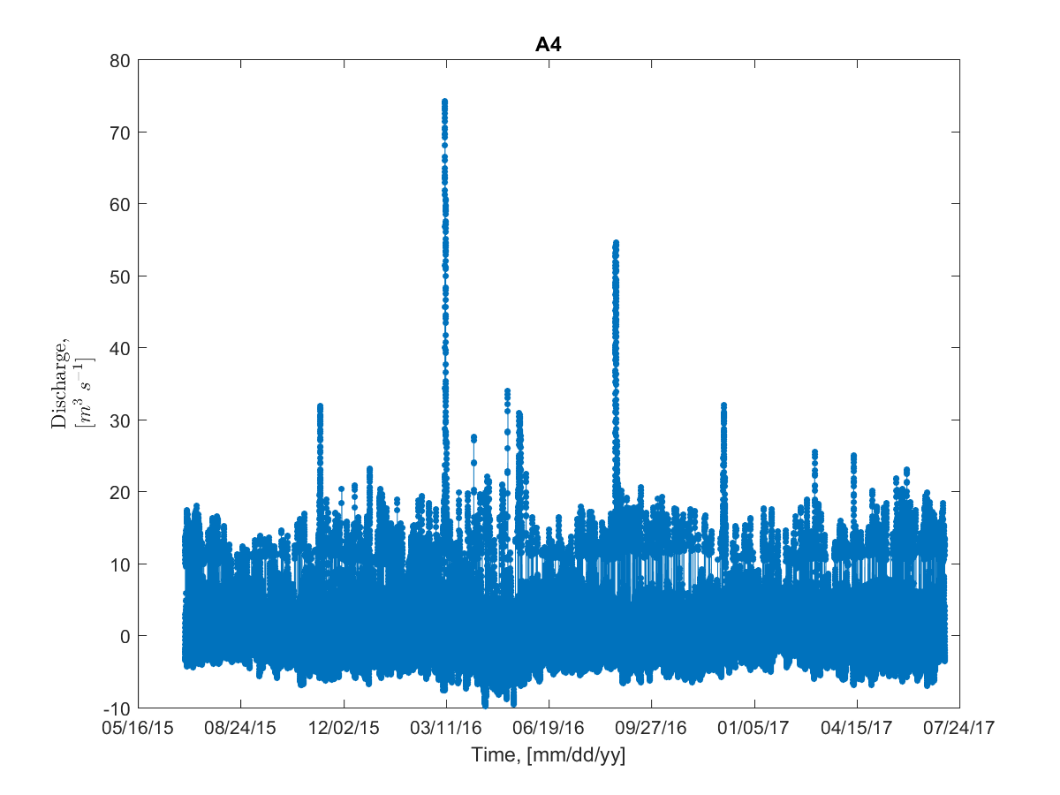


**Figure D.4**: The calculated discharge time series for M-A site: A4.


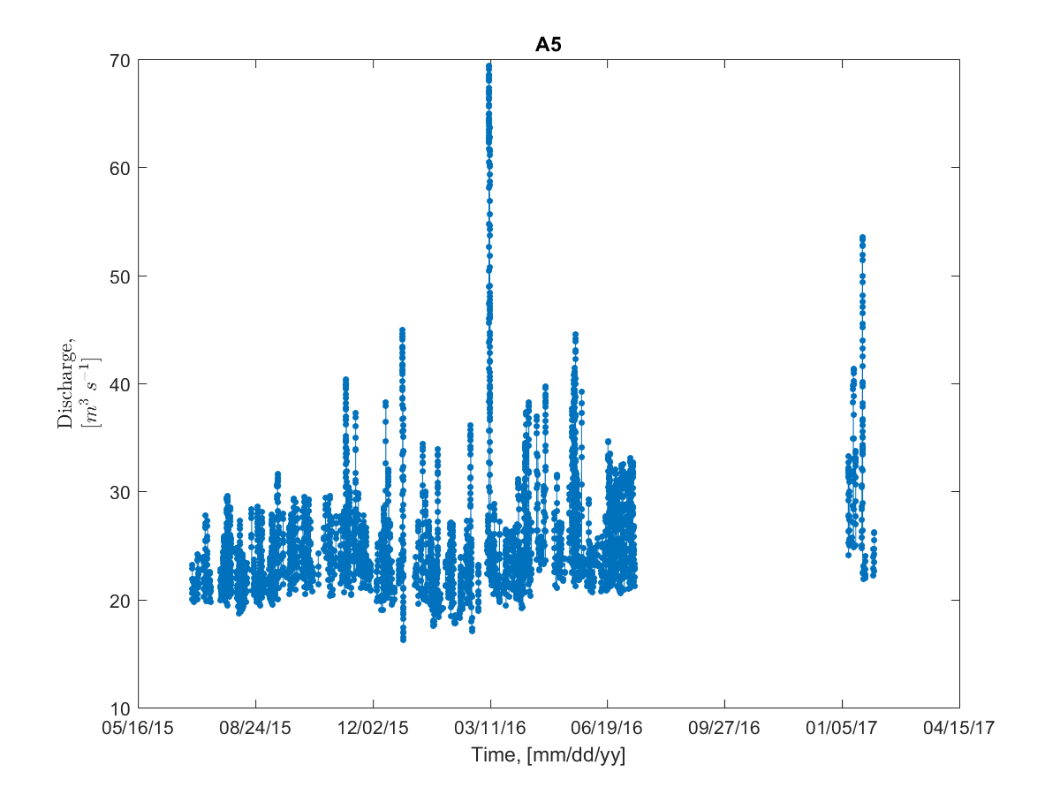


**Figure D.5**: The calculated discharge time series for M-A site: A5. There are no baseflow discharge conditions calculated for this site, because no ADP transects were completed at this site due to safety concerns. All that is presented are predicted storm discharges.


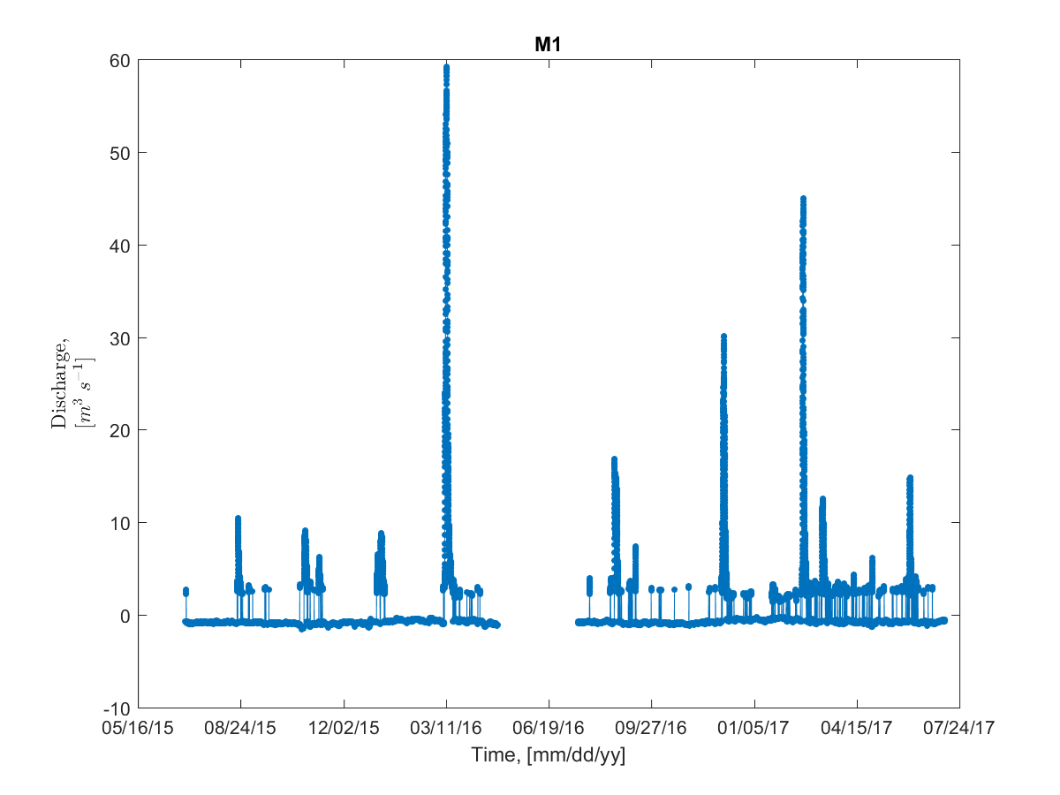


**Figure D.6**: The calculated discharge time series for M-A site: M1.


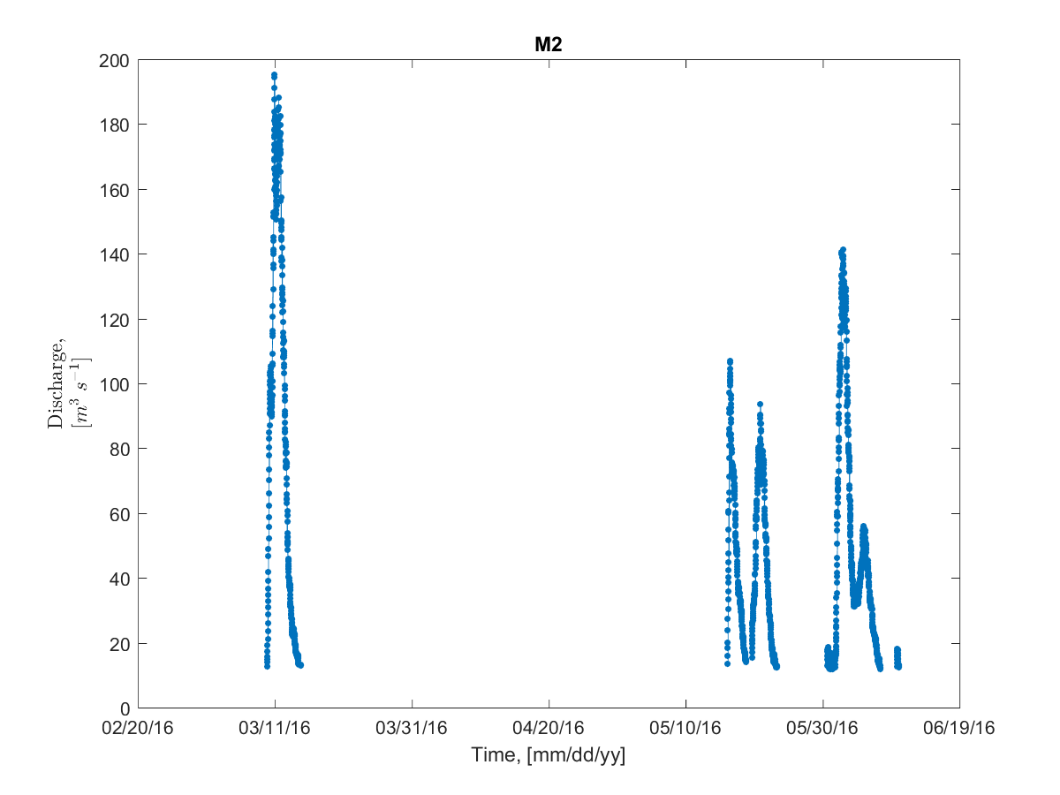


**Figure D.7**: The calculated discharge time series for M-A site: M2. Baseflow discharge was not calculated for this sites due to multiple sensor malfunctions and insufficient ADP transects. The discharges presented are only predictions of storm discharge.


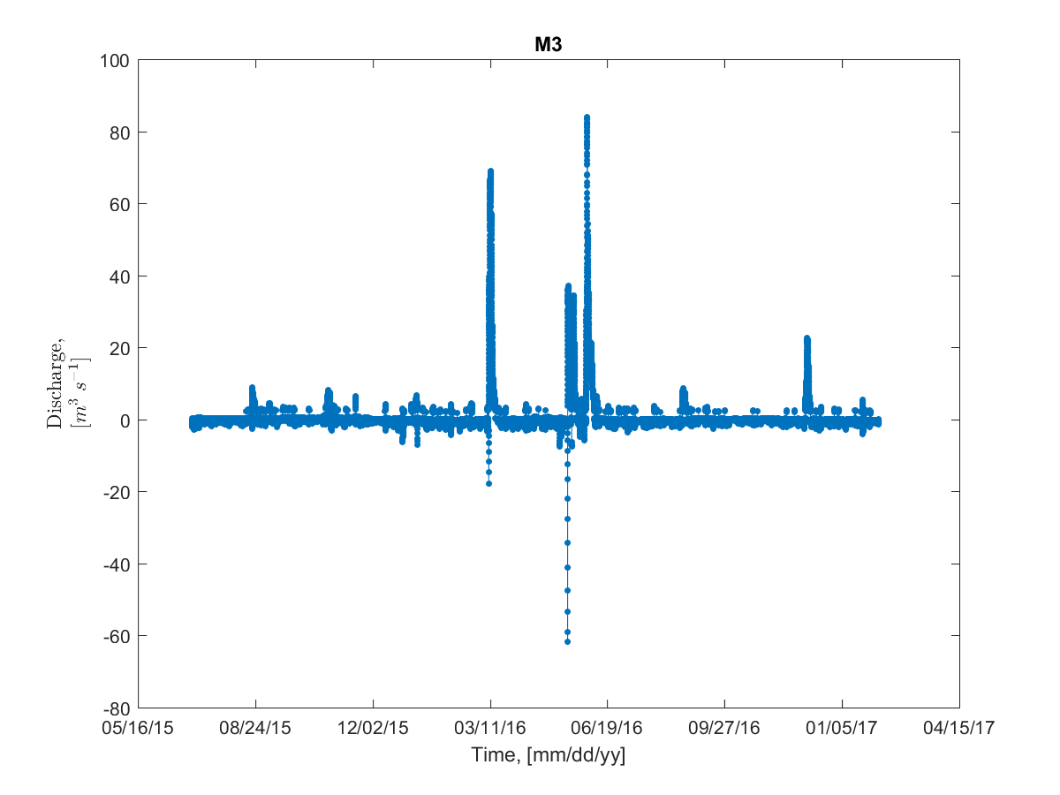


**Figure D.8**: The calculated discharge time series for M-A site: M3. The spike in upstream discharge near 16 May 2016 is related to the rising limb of storm dramatically altering the dS conditions before shifting the cross-sectional velocity towards storm conditions. This meant that the simplistic method for discerning storm v. baseflow periods calculated discharge with the baseflow equation (eq. 3) and coefficients producing a negative (i.e., upstream) discharge associated with sharp rise in stage (i.e., large magnitude positive dS).


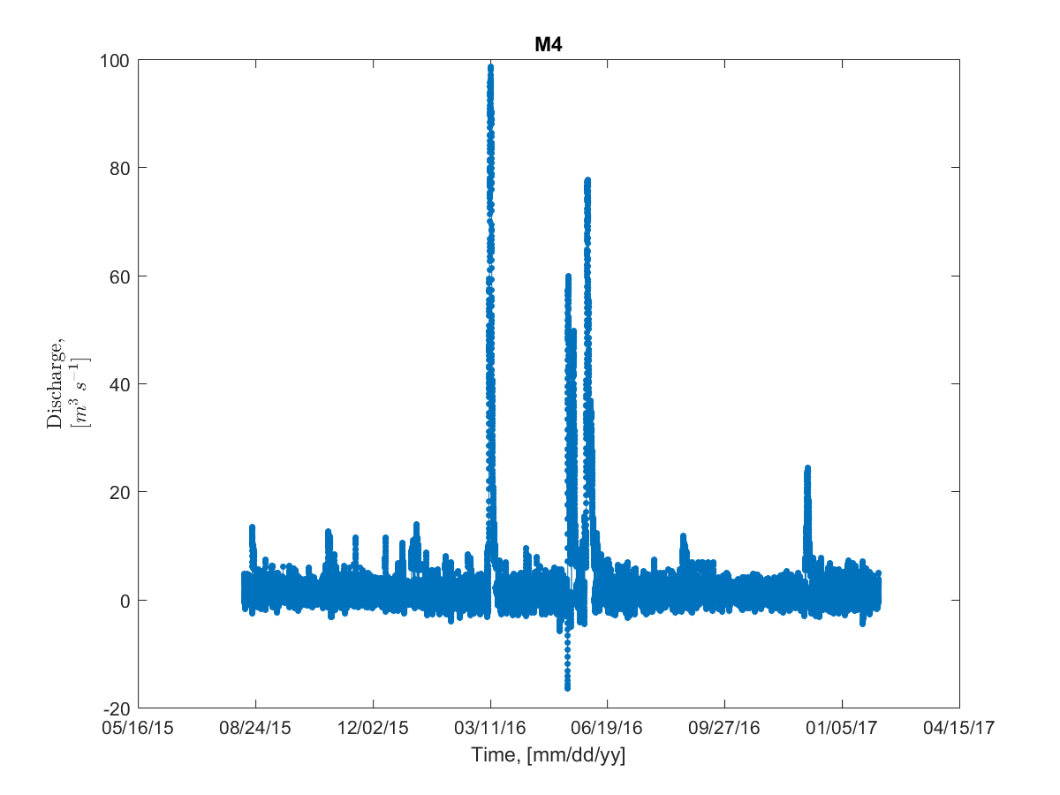


**Figure D.9**: The calculated discharge time series for M-A site: M4.


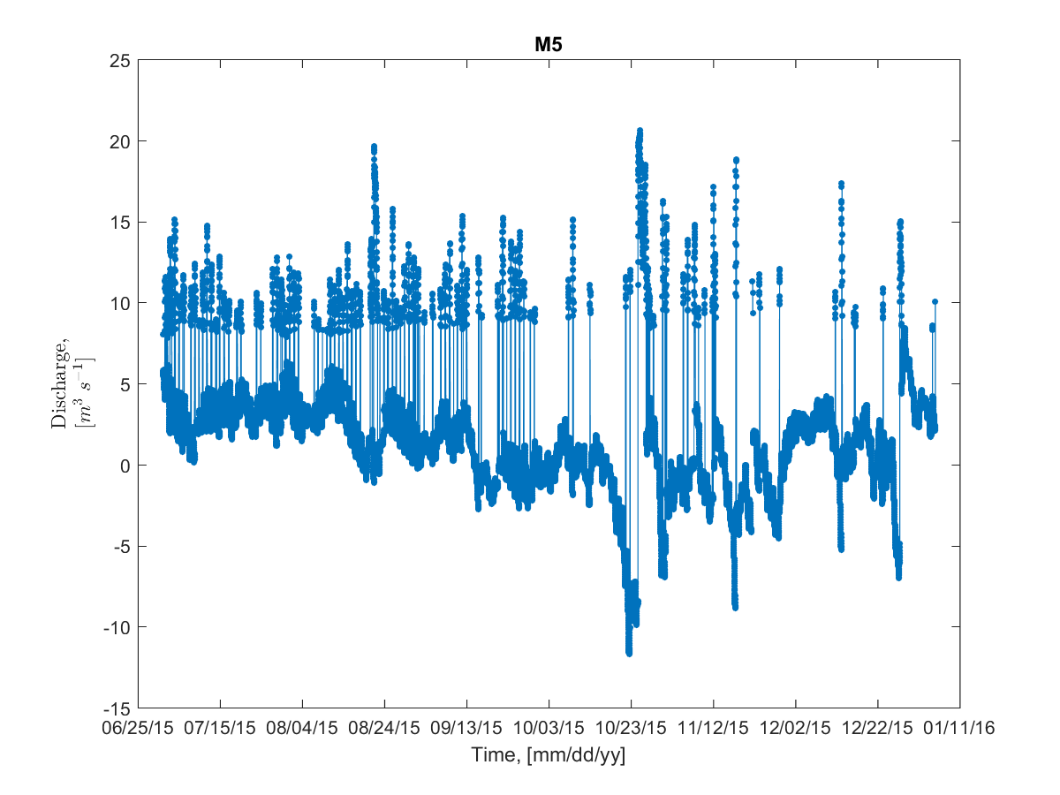


**Figure D.10**: The calculated discharge time series for M-A site: M5. Unfortunately, throughout much of the study period the TCM for this site was malfunctioning. Thus, this is a very incomplete record of discharge, one that may not have any actual storm periods.

# E. M-A assessment of standing wave

For systems exhibiting an endmember waveform, tidal baseflow may be estimated by fitting a linear model between observed discharge and the related variable (i.e., $\frac{dS}{dt}$ for standing waves, and stage for progressive waves). To demonstrate this point, we constructed linear models relating only $\frac{dS}{dt}$ conditions to ADP discharge observations for each of the eight standing wave M-A sites (Figure E.1). That is, we fit the model:

$Q_{ADP}={k'}_{4}\frac{{dS}_{i}}{dt}$ (E.1)

Each linear model assumes a y-intercept of zero, which assumes that no change in stage relates to zero discharge. For a standing wave system, a $\frac{dS}{dt}$ value of zero corresponds to either high or low tide (HW/LW), where stage momentarily halts before reversing directions. This moment of zero tidal discharge during the reversal of the stage is known as slack water (SW). These simplified models resulted in significant p-values (p < 0.05) and the expected inverse relationship between discharge and $\frac{dS}{dt}$. Several sites showed a strong relationship (i.e., R^2^ > 0.45) between $\frac{dS}{dt}$ and ADP observations (i.e., A2, A3, A4, M3 and M5). The exceptionally low R^2^ from site M1 is likely due to many ADP observations occurring under similar $\frac{dS}{dt}$ conditions, resulting in insufficient variance in the data.

The results of the full (eq. 3) and standing wave models (eq. E.1) are similar with regards to R^2^ and modeled coefficients, specifically k_4_ and $k_{4}^{'}$ (Table 2, Figure E.1). In both models, sites M4 and A3 exhibited the greatest R^2^, while the most upstream sites (A1 and M1) exhibited the lowest R^2^. The weak correlations at sites A1 and M1 was likely due to minimal discharge magnitude and lack of $\frac{dS}{dt}$ variance observed at site M1. The five M-A k_4_ values from Table 2 were very similar to the corresponding $k_{4}^{'}$ values in Figure E.1, especially for the Aransas River. A ratio of the coefficients, $\rho=k_{4}:k_{4}^{'}$, ranged from 0.62 at M4 to 1.03 at A3, with all of the Aransas River sites exhibiting ρ ≥ 0.70 (i.e., ρ_A1_ = 0.84, ρ_A2_ =0.70, ρ_A3_ = 1.03, and ρ_A4_ = 0.97). A ρ-value of 1.0 would indicate matching coefficients. The similarity of calculated k_4_ and $k_{4}^{'}$ terms confirms that the baseflow model (eq. 3) for these five sites is heavily dependent on the $\frac{dS}{dt}$ term, as expected for strongly standing wave systems.

Although experiencing a standing wave, baseflow models of sites M1 and M5 do not relate to $\frac{dS}{dt}$ (Table 2). Insufficient variability in $\frac{dS}{dt}$ conditions during ADP observations likely explains these odd relationships (Figure E.1e and E.1h). For M5, ADP observations clustered into two groups of $\frac{dS}{dt}$ conditions (Figure E.1h), where stage observations (R^2^ = 0.51, Table 2) explained more of the variability than $\frac{dS}{dt}$ (R^2^ = 0.47, Figure E.1h). More ADP transects over a greater diversity of $\frac{dS}{dt}$ likely would provide a relationship that coincides with standing wave theory.


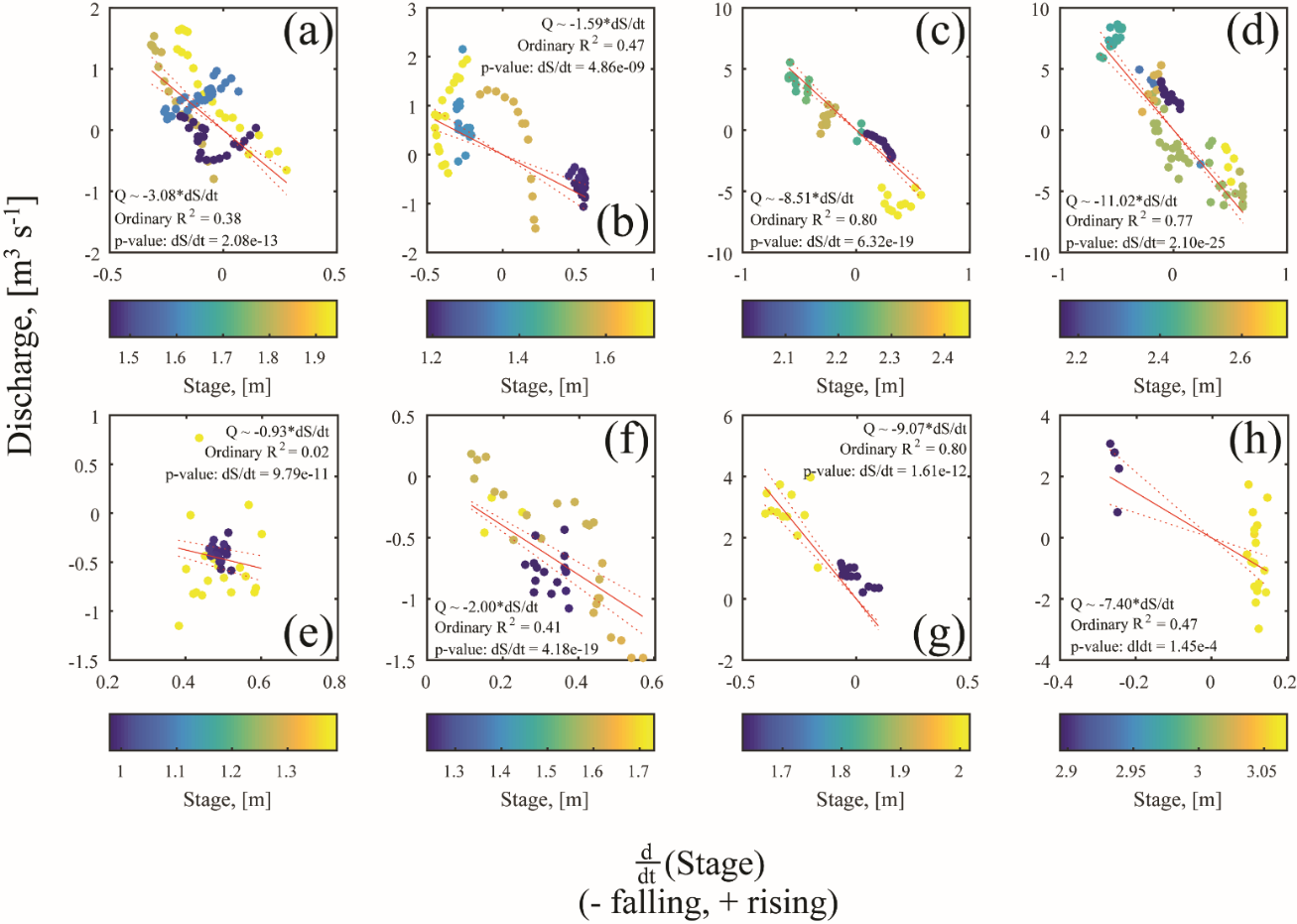


**Figure E.1:** Linear models relating the stage-rate-of-change to ADP discharge observations along the Aransas and Mission Rivers, Texas. Each linear model assumes the origin is the y-intercept. The Aransas River sites, A1-4, are depicted in the top row of subfigures (a-d), while the Mission River sites M1 and M3-5 are portrayed on the lower row (subfigures e-h), respectively. In general, each site shows a fair-to-strong relationship between discharge and stage-rate-of-change conditions, which reflects each site’s φ values and strongly standing wave-like character; for an ideal standing wave we should expect a linear relationship. The data are colored in reference to their corresponding stage values.

# F. Why not relate discharge to TCM velocity?

Figure F.1 displays the correlations and regressions between discharge and TCM velocity for sites A1 (Figure F.1a), A3 (Figure F.1b), and A4 (Figure F.1c). The gray dashed lines in each subfigure portray the 2 cm s^-1^ sensitivity of the TCM.

Each site’s regression coefficient is significantly different from zero when using an intercept at the origin, with all p < 0.05 (4.13x10^-12^, 2.48x10^-15^, and 1.18x10^-8^, respectively). However, the regression fit (i.e., R^2^) varies substantially between upstream (i.e., A1) and downstream (i.e., A3 and A4) sites. The downstream sites exhibit coefficients of determination of 0.73 and 0.48, respectively, as opposed to the R^2^ of 0.03 at site A1. The discrepancy in the R^2^ values between the sites is primarily due to the removal of unreliable velocity observations (i.e., | *v_i_* | *<* 2 cm s^-1^) at A1 because of insufficient instrument sensitivity. The overall larger magnitude of velocity observations at downstream sites led to better regressions.


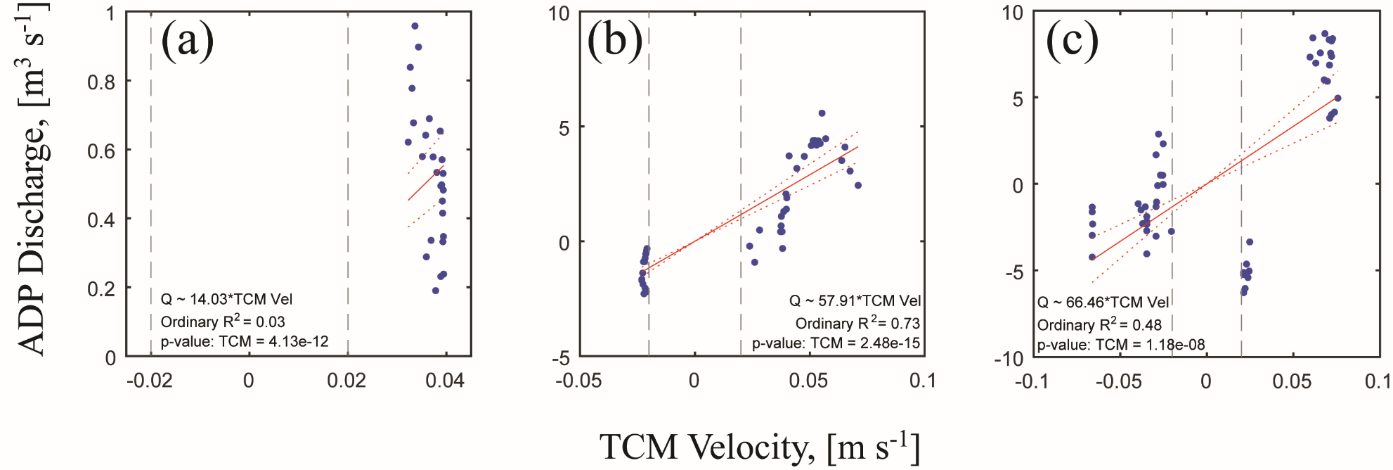


**Figure F.1:** Linear model relating TCM velocity to ADP discharge observations at three sites along the Aransas River, Texas. TCM velocities for sites A1 (a), A3 (b), and A4 (c) increase in magnitude with increased distance downstream (toward A4). The regressions for the two downstream sites, A3 and A4, present much better coefficients of determination (R^2^ = 0.73 and 0.48, respectively) than the upstream-most site, A1 (R^2^ = 0.03). This is primarily due to the velocity observed at A1 often falling below the TCM sensitivity and, therefore, being unreliable in the model.

# G. Table of Ecological and Climate Zones

**Table G.1:** ; List of analyzed Mission-Aransas and USGS tidal sites, site numbers, figure (1 and 3) labels, North American ecological region [US EPA 2015] and Köppen-Geiger climate type [Peel et al. 2007] of station, mean discharge between July 2015-July 2017, calculated φ (radians and degrees), and assessed waveform.

| **Site Name** | **Site Number** | **Label** | **North American  Ecological Region**  **(Level 1, 2, 3)** | **Köppen-Geiger climate type** | **Mean Discharge [m^3^ s^-1^]** | **Phase offset [Rad]** | **Phase Offset [deg]** | **Wave Type** |
| --- | --- | --- | --- | --- | --- | --- | --- | --- |
| Aransas R 1  (upstream) | M-A Site | A1 | Great Plains, Texas-Louisiana coastal plain, Western Gulf coastal plain | Warm Oceanic climate/ Humid subtropical climate (Cfa) | 0.270 ± 1.363**^+^** | $\frac{\pi}{2.122}$ | 84.84 | *Mixed |
| Aransas R 2 | M-A Site | A2 |  |  |  | $\frac{\pi}{1.868}$ | 96.34 | Mixed |
| Aransas R 3 | M-A Site | A3 |  |  |  | $\frac{\pi}{2.109}$ | 85.36 | Standing |
| Aransas R 4 | M-A Site | A4 |  |  |  | $\frac{\pi}{2.104}$ | 85.54 | Standing |
| Aransas R 5  (downstream) | M-A Site | A5 |  |  |  | $\frac{\pi}{1.983}$ | 90.77 | Standing |
| Mission R 1  (upstream) | M-A Site | M1 |  |  | 1.153 ± 5.509**^+^** | $\frac{\pi}{2.048}$ | 87.88 | Standing |
| Mission R 2 | M-A Site | M2 |  |  |  | ** | ** | ** |
| Mission R 3 | M-A Site | M3 |  |  |  | $\frac{\pi}{2.307}$ | 78.04 | *Mixed |
| Mission R 4 | M-A Site | M4 |  |  |  | $\frac{\pi}{2.136}$ | 84.28 | *Mixed |
| Mission R 5  (downstream) | M-A Site | M5 |  |  |  | $\frac{\pi}{1.911}$ | 94.20 | Standing |
|  |  |  |  |  |  |  |  |  |
| Connecticut River | 01193050 | CON | Eastern temperate forests, Mixed wood plains, Northeastern coastal zone | Warm continental climate/  Humid continental climate (Dfa) | 501.110 ± 520.754 | $\frac{\pi}{1.393}$ | 129.20 | Mixed |
| Plum Island River | 424752070491701 | PLU |  | Temperate continental climate/ Humid continental climate (Dfb) | 2.774 ± 57.366 | $\frac{\pi}{2.153}$ | 83.61 | *Mixed |
| Murderkill River | 01484085 | MUR | Eastern temperate forests, Mississippi alluvial and southeast USA coastal plains, Middle Atlantic coastal plain | Warm Oceanic climate/ Humid subtropical climate (Cfa) | 2.619 ± 128.330 | $\frac{\pi}{1.244}$ | 144.68 | Mixed |
| Middle River | 02198950 | MID | Eastern temperate forests, Mississippi alluvial and southeast USA coastal plains, Southern coastal plain |  | 30.114 ± 255.325 | $\frac{\pi}{1.632}$ | 110.31 | Mixed |
| Savannah River | 02198980 | SAV |  |  | 479.196 ± 4642.285 | $\frac{\pi}{1.571}$ | 114.55 | Mixed |
| Ogeechee River | 02203536 | OGE |  |  | 90.399 ± 253.478 | $\frac{\pi}{1.595}$ | 112.88 | Mixed |
| St. Mary's River | 02231254 | STM |  |  | 40.381 ± 559.885 | $\frac{\pi}{1.317}$ | 136.72 | Mixed |
| Chassahowitzka River | 02310663 | CHA |  |  | 2.462 ± 21.228 | $\frac{\pi}{1.766}$ | 101.90 | Mixed |
| Halls River | 02310689 | HAL |  |  | 0.906 ± 4.022 | $\frac{\pi}{1.658}$ | 108.59 | Mixed |
| Crystal River | 02310747 | CRY |  |  | 5.021 ± 129.103 | $\frac{\pi}{1.998}$ | 90.08 | Standing |
| Mobile River | 02470629 | MOB | Eastern temperate forests, Southeastern USA plains, Southeastern plains |  | 653.999 ± 530.600 | $\frac{\pi}{1.279}$ | 140.78 | Mixed |
| Shark River | 252230081021300 | SHA | Tropical wet forests, Everglades, Southern Florida coastal plain | Tropical savannah climate (Aw) | 9.603 ± 143.226 | $\frac{\pi}{1.217}$ | 147.92 | Mixed |

^+^ For M-A sites, discharge is reported from each river’s nearest USGS gauging station, which are non-tidal (Aransas ID: 08189700; Mission ID: 08189500).
* Phase offsets are likely of strongly standing character, even though slightly beyond ± 5 degree margin.
** Insufficient TCM velocity data to accurately determine φ.

# H. Table of NOAA Tidal Gauging nearest to sites

**Table H.1:** Reported mean ranges for the nearest tidal gauging sites to each river gauging site of interest [NOAA 2017b].

| **Site Name** | **Site Number** | **Mean Tidal Range (TR), [m]**** | | **NOAA Tidal Site** |
| --- | --- | --- | --- | --- |
| Connecticut Rv | 01193050 | 0.78 | New London, Thames River, CT - Station ID: 8461490 | |
| Plum Island Rv | 424752070491701 | 2.63 | Fort Point, NH - Station ID: 8423898 | |
| Murderkill Rv | 01484085 | 1.49 | Brandywine Shoal Light, DE - Station ID: 8555889 | |
| Middle Rv | 02198950 | 2.11 | Fort Pulaski, GA - Station ID: 8670870 | |
| Savannah Rv | 02198980 | 2.11 | Fort Pulaski, GA - Station ID: 8670871 | |
| Ogeechee Rv | 02203536 | 2.11 | Fort Pulaski, GA - Station ID: 8670872 | |
| St. Mary’s Rv | 02231254 | 1.83 | Fernandina Beach, FL - Station ID: 8720030 | |
| Chassahowitzka Rv | 02310663 | 0.86 | Cedar Key, FL - Station ID: 8727520 | |
| Halls Rv | 02310689 | 0.86 | Cedar Key, FL - Station ID: 8727521 | |
| Crystal Rv | 02310747 | 0.86 | Cedar Key, FL - Station ID: 8727522 | |
| Mobile Rv | 02470629 | 0.45 | Mobile State Docks, AL - Station ID: 8737048 | |
| Shark Rv | 252230081021300 | 0.22 | Vaca Key, Florida Bay, FL - Station ID: 8723970 | |
| Mission &Aransas Rv | n/a | 0.11 | Rockport, TX - Station ID: 8774770 | |

# I. Table of USGS Calibration and Validation dates

**Table I.1:** Dates selected for calibration and validation at each USGS site.

| **Site Name** | **Site Number** | **Calibration Dates** | **Validation Dates** |
| --- | --- | --- | --- |
| Connecticut Rv | 01193050 | 14 – 29 Aug. 2015 | 14 – 29 Aug. 2016 |
| Plum Island Rv | 424752070491701 | 23 Oct. – 08 Nov. 2015 | 05 – 20 Dec. 2016 |
| Murderkill Rv | 01484085 | 14 – 29 April 2015 | 04 – 19 Sept. 2016 |
| Middle Rv | 02198950 | 11 – 25 Jan. 2016 | 11 – 26 Jan. 2016 |
| Savannah Rv | 02198980 | 20 Apr. – 05 May 2016 | 20 Apr. – 05 May 2017 |
| Ogeechee Rv | 02203536 | 28 Apr. – 13 May 2016 | 14 – 29 June 2016 |
| St. Mary’s Rv | 02231254 | 20 Dec. 2015 – 04 Jan. 2016 | 16 – 31 Dec. 2016 |
| Chassahowitzka Rv | 02310663 | 20 Aug. – 04 Sep. 2015 | 20 Apr. – 05 May 2016 |
| Halls Rv | 02310689 | 27 Jun. – 11 Jul. 2016 | 09 – 24 Aug. 2015 |
| Crystal Rv | 02310747 | 07 – 22 Sept. 2015 | 03 – 18 Mar. 2016 |
| Mobile Rv | 02470629 | 13 – 28 Sept. 2016 | 04 – 19 Aug. 2015 |
| Shark Rv | 252230081021300 | 09 – 24 Mar. 2016 | 09 – 24 Dec. 2016 |

# J. Additional MATLAB model files

File 1: The following file was used to perform the phase analysis.

======== BEGIN FILE ========

%{

Phase analysis.

Author: Allan Jones

Date: 9/4/2017

%}

clear; clc; close all

% determining which computer is being used

cur_dir = cd; slashes = regexp(cur_dir, '\');

comp_using = cur_dir(1:slashes(3));

save_fig = 10;

write_save = 10;

%% SAVE INFO

save_folder = [date '_plots\'];

save_path = [comp_using 'Dropbox\Field Work\Analysis\'...

'Field Data Analysis\TCM rating curve\Figures\' save_folder];

if ~isdir([save_path])

mkdir([save_path]);

end

% write .csv files

if write_save ~= 0

csvpath = save_path;

% [comp_using 'Dropbox\Field Work\Analysis\'...

% 'Field Data Analysis\TCM rating curve\MA Regression Info\'];

labelcsv = fopen([csvpath 'all_sites_phase.csv'],'w');

% write headers

fprintf(labelcsv, ['Site Name, Site Number, Label, '...

'Phase Offset [deg], Wave Type\r\n']);

end

%initiate plot

figure(1e5)

%% M-A Analysis

% Loading all the calibrated TCM data

% creating final_master (combining all deployments) from site data

disp(['Loading calibrated TCM data and performing moving average...'])

soi = {'A1', 'A2', 'A3', 'A4', 'A5','M1', 'M2', 'M3', 'M4', 'M5'};

min15 = 15/(24*60);

%%%%%%%%%%%%%%%%% - ARANSAS RIVER

% site cleaning thresholds - [qlow, qhi, slow, shi]

sites.A1.thresholds = [-35, 250, 1.10, 2.7];

sites.A2.thresholds = [-35, 60, 1.10, 2.7];

sites.A3.thresholds = [-50, 90, 1.75, 3.1];

sites.A4.thresholds = [-50, 60, 1.75, 3.25];

sites.A5.thresholds = [-50, 40, 2.2, 3.75];

% site start and end timestamps

sites.A1.times = [datenum('20150701 00:00', 'yyyymmdd HH:MM'),...

datenum('20170701 00:00', 'yyyymmdd HH:MM')];

sites.A2.times = [datenum('20150701 00:00', 'yyyymmdd HH:MM'),...

datenum('20160701 00:00', 'yyyymmdd HH:MM')];

sites.A3.times = [datenum('20150701 00:00', 'yyyymmdd HH:MM'),...

datenum('20160701 00:00', 'yyyymmdd HH:MM')];

sites.A4.times = [datenum('20150701 00:00', 'yyyymmdd HH:MM'),...

datenum('20170701 00:00', 'yyyymmdd HH:MM')];

sites.A5.times = [datenum('20150701 00:00', 'yyyymmdd HH:MM'),...

datenum('20160701 00:00', 'yyyymmdd HH:MM')];

%%%%%%%%%%%%%%%%% - MISSION RIVER

% site cleaning thresholds - [qlow, qhi, slow, shi]

sites.M1.thresholds = [-35, 250, 0.75, 5.5];

sites.M2.thresholds = [-35, 250, 0.75, 5.5];

sites.M3.thresholds = [-20, 90, 1.00, 5.5];

sites.M4.thresholds = [-30, 90, 1.25, 5.5];

sites.M5.thresholds = [-50, 40, 2.2, 5.5];

% site start and end timestamps

sites.M1.times = [datenum('20160720 00:00', 'yyyymmdd HH:MM'),...

datenum('20170710 00:00', 'yyyymmdd HH:MM')];

sites.M2.times = [datenum('20160303 00:00', 'yyyymmdd HH:MM'),...

datenum('20160710 00:00', 'yyyymmdd HH:MM')];

sites.M3.times = [datenum('20150701 00:00', 'yyyymmdd HH:MM'),...

datenum('20160701 00:00', 'yyyymmdd HH:MM')];

sites.M4.times = [datenum('20150701 00:00', 'yyyymmdd HH:MM'),...

datenum('20160701 00:00', 'yyyymmdd HH:MM')];

sites.M5.times = [datenum('20150625 00:00', 'yyyymmdd HH:MM'),...

datenum('20160104 00:00', 'yyyymmdd HH:MM')];

% create a path for loading data

load_path = ['Dropbox\Field Work\Analysis\All Long Term Data\'...

'Matlab Cleaned Data\Calibrated TCM data\'];

data_saving = 'prelim_20170725\TCM matrix-only data\';

fftwindow = 25; % moving average window -> (dldt + stage - 1)

% 9 - +/- 1 hour

% 13 - +/- 1.5 hours

% 17 - +/- 2 hours

% 25 - +/- 3 hours

% 51 - +/- 6 hours -> 12 hours total

min15 = 15/(24*60);

%7{

% load each site's data and create a final_master matrix from deployments

% determine dl/dt for each site's data

for vvv = 1:length(soi) % [1, 3, 4] % 4 % 1:4 % [1, 3, 4] %

disp(['Loading site: ' soi{vvv}])

% loading saved TCM/LTC data

eval(['load([comp_using load_path data_saving '...

'soi{vvv} '...

'''_calib_mat_only.mat''])'])

% Making data ONE LARGE data variable

eval(['data = ' soi{vvv} '_mat_only;'])

all_data = [nanmean([data(:,1), data(:,5)],2),... % averaged LTC and TCM timestamps

data(:,17),... % TCM velocities [cm s-1]

data(:,2)]; % LTC stage [m]

% remove bizarre points

eval(['threshs = sites.' soi{vvv} '.thresholds;'])

idx = find( (all_data(:,2) < threshs(1)) | (all_data(:,2) > threshs(2)) );

all_data(idx, :) = [];

% stages

idx = find( (all_data(:,3) < threshs(3)) | (all_data(:,3) > threshs(4)) );

all_data(idx, :) = [];

% linearly interpolate "all data" onto 15 minute intervals

eval(['start = sites.' soi{vvv} '.times(1);'])

eval(['ender = sites.' soi{vvv} '.times(2);'])

timeline = [start-1:min15:ender+1]';

idata = nan( length(timeline), size(all_data,2) );

idata(:,1) = timeline;

idx = find( ~isnan(all_data(:,2)));

idata(:,2) = interp1(all_data(idx,1), all_data(idx,2), timeline);

idx = find( ~isnan(all_data(:,3)));

idata(:,3) = interp1(all_data(idx,1), all_data(idx,3), timeline);

% perform moving average on data

fftavgdata = nan( length(idata), size(idata,2) );

fftavgdata(:,1) = aej_moving_avg(idata(:,1), fftwindow);

fftavgdata(:,2) = aej_moving_avg(idata(:,2), fftwindow);

fftavgdata(:,3) = aej_moving_avg(idata(:,3), fftwindow);

dldt = [(idata(2:end,3) - idata(1:end-1,3))./...

(idata(2:end,1) - idata(1:end-1,1)); nan];

fftavgdata(:,4) = aej_moving_avg(dldt, fftwindow);

% select the data that is between longest continuous time period

idx = find( (fftavgdata(:,1) >= start) & (fftavgdata(:,1) < ender) );

annum = fftavgdata(idx,:);

% perform the RFFT on the annum data

%{

% sampling frequency = 15 minutes

% sF = min15;

stage_fft = fft( annum(:,3) );

velocity_fft = fft( annum(:,2) );

% determining the frequencies of the FFT

if rem(length(stage_fft)-1,2) == 0

aaa = 1+(length(stage_fft)-1)/2;

else

aaa = 1+(length(stage_fft)-2)/2;

end

freqs = (0:aaa)./(ender-start); % # of cycles/time periods [days]

periods = (1./freqs);

%% Identify phase offset

% isolate indices of important periods - [0, 12.4/24, 23.9/24, 25.8/24, 183, 366]

if strcmp(soi{vvv}(1), 'A') || strcmp(soi{vvv}, 'M3')

imppds = [0, 12.423/24, 23.935/24, 25.800/24, 183, 366]; % from NOAA

else

% for the incomplete datasets of the Mission, we want thetwo

% longest frequencies to help isolate the proper semidiurnal and

% diurnal frquencies.

imppds = [0, 12.423/24, 23.935/24, 25.800/24, (ender-start)/2, (ender-start)];

end

ipds = ones(size(imppds));

for iii = 2:length(ipds)

[~, ipds(iii)] = min( abs(periods - imppds(iii)) );

end

% determine phase offset from indices(2:4)

stage_phase = (angle(stage_fft(ipds(2:4))));

velocity_phase = (angle(velocity_fft(ipds(2:4))));

%{

for iii = 1:length(stage_phase)

if stage_phase(iii) < 0

stage_phase(iii) = 2*pi-abs(stage_phase(iii));

end

if velocity_phase(iii) < 0

velocity_phase(iii) = 2*pi-abs(velocity_phase(iii));

end

end

%}

phase_offset = abs(stage_phase - velocity_phase);

phi_value = mean(phase_offset);

if phi_value > pi+(5/180)*pi

% re-write offset if greater than pi

% a phase of +(3*pi/2), and -pi/2 are the same, but unique to FFT

% absolute value should often eliminate that problem, but not always

phi_value = 2*pi - phi_value;

end

%}

[phi_value, mthm] = FXN_USGS_phase( save_path, save_fig,...

'MA_FFTphase\',...

annum(:,1), annum(:,2), annum(:,3), soi{vvv} );

%% Writing M-A phi

% writing phi data to .csv

wave_type = 'Mixed';

if write_save ~= 0

if phi_value*(180/pi) <= 95 & phi_value*(180/pi) >= 85

wave_type = 'Standing';

elseif phi_value*(180/pi) <= 185 & phi_value*(180/pi) >=175

wave_type = 'Progressive';

elseif phi_value*(180/pi) <= 85

wave_type = '*Standing';

end

if strcmp(soi{vvv},'M2') %|| strcmp(soi{vvv},'M5')

fprintf(labelcsv, '%s, %s, %s, %s, %s\r\n',...

soi{vvv},...

'M-A Site',...

soi{vvv},...

'-**',...

'-**');

else

fprintf(labelcsv, '%s, %s, %s, %.4f, %s\r\n',...

soi{vvv},...

'M-A Site',...

soi{vvv},...

phi_value*(180/pi),...

wave_type);

end

end

%% plot data

if ~strcmp(soi{vvv},'M2') % && ~strcmp(soi{vvv},'M5')

figure(1e5)

plot(mthm,... % max( abs([stage_fft(ipds(2:end-2)); velocity_fft(2:end-2)]) ),...

phi_value, '.k', 'markersize', 15)

hold on

text(mthm,... % max( abs([stage_fft(ipds(2:end-2)); velocity_fft(2:end-2)]) ),...

phi_value, soi{vvv}, 'VerticalAlignment', 'bottom',...

'HorizontalAlignment', 'center', 'color', 'k')

end

end

%}

%% USGS analysis

% path to the .txt files

USGSpath = [comp_using 'Dropbox\Field Work\Analysis\Field Data Analysis\'...

'TCM rating curve\USGS Tidal Sites\USGS Tidal Dnlds\TXT files'];

% obtaining the filenames from the

filenames = dir(USGSpath);

% cleaning odd structure fields

filenames(1) = []; filenames(1) = [];

%% looping through the filenames

for fff = 1:length(filenames) % [1, 2, 16, 17] % 2 %

% obtaining sitenumber and rivername from filenames

underscore = regexp(filenames(fff).name, '_');

sitenum = filenames(fff).name(1:underscore(1)-1);

rvname = filenames(fff).name(underscore(1)+1:underscore(3)-1);

% update to user

disp(['Obtaining data for USGS ' sitenum ': ' strrep(rvname, '_', ' ')])

% obtaining the site data

data = obtain_rv_data([USGSpath '\' filenames(fff).name],...

rvname,...

sitenum);

data = data{2}; % keeping only [time, discharge, stage]

% find longest time period of unified data

% (matches length of shorter dataset)

% checking if dates have "nan" gaps

%{

if ~isempty(find(diff(find(~isnan(data(:,3))))>1)) ||...

~isempty(find(diff(find(~isnan(data(:,2))))>1))

% find returns the value before

snan = find(~isnan(data(:,3)));

istage = [[snan(1); snan(find(diff(snan)>1)+1)] ,...

[snan(find(diff(snan)>1)); snan(end)],...

ones(length(find(diff(snan)>1))+1,1)];

% removing very short gaps (< 1day) in data

iii = 2;

while iii <= size(istage, 1)

if data(istage(iii,1),1) - data(istage(iii-1,2),1) < 1.5

istage(iii-1,2) = istage(iii,2);

istage(iii,:) = [];

else

iii = iii + 1;

end

end

% find longest stage data

[msl.value, msl.idx] = max( istage(:,2) - istage(:,1) );

% find returns the value before

dnan = find(~isnan(data(:,2)));

idis = [[dnan(1); dnan(find(diff(dnan)>1)+1)],...

[dnan(find(diff(dnan)>1)); dnan(end)],...

2.*ones(length(find(diff(dnan)>1))+1,1)];

% removing very short gaps (< 1day) in data

iii = 2;

while iii <= size(idis, 1)

if data(idis(iii,1),1) - data(idis(iii-1,2),1) < 1.5

idis(iii-1,2) = idis(iii,2);

idis(iii,:) = [];

else

iii = iii + 1;

end

end

% find longest stage data

[mdl.value, mdl.idx] = max( idis(:,2) - idis(:,1) );

% is one longest data within the other?

% discharge within stage

if (istage(msl.idx,2) >= idis(mdl.idx,2) &&...

istage(msl.idx,1) <= idis(mdl.idx,1))

% use shorter dataset to "grab" data - discharge

start = data(idis(mdl.idx,1),1);

ender = data(idis(mdl.idx,2),1);

% flip the signs: stage within discharge

elseif (istage(msl.idx,2) <= idis(mdl.idx,2) &&...

istage(msl.idx,1) >= idis(mdl.idx,1))

% use shorter dataset to "grab" data - stage

start = data(istage(msl.idx,1),1);

ender = data(istage(msl.idx,2),1);

else % longest time periods do not overlap...

% choosing later start and earlier end

if istage(msl.idx,1) > idis(mdl.idx,1) % after

start = data(istage(msl.idx,1),1);

else

start = data(idis(mdl.idx,1),1);

end

if istage(msl.idx,2) < idis(mdl.idx,2) % before

ender = data(istage(msl.idx,2),1);

else

ender = data(idis(mdl.idx,2),1);

end

% error('ERROR: The data sets do not overlap...')

end

else

% if no "nan"-values are present

istage = find(~isnan(data(:,3)));

start = data(istage(1),1);

ender = data(istage(end),1);

end

% % would prefer multiples of whole year data

% % && setting important periods

% imppds = [0, 12.423/24, 23.935/24, 25.800/24, 183, 366]; % from NOAA - others: 12/24, 12.65/24,

% if rem(round(ender-start), 366) > 10 % NOT nearly annual or two year

% if (ender-start) > 350

% ender = start + 366;

% else

% imppds = [0, 12.423/24, 23.935/24, 25.800/24,...

% (ender-start)/2, ender-start]; % others: 12/24, 12.65/24,

% end

% end

%

%}

%% empirically select best time period

if ~isempty(regexp(lower(rvname), 'plum'))

start = datenum('20150825', 'yyyymmdd');

ender = datenum('20160706', 'yyyymmdd');

elseif ~isempty(regexp(lower(rvname), 'shark'))

start = datenum('20151001', 'yyyymmdd');

ender = datenum('20161002', 'yyyymmdd');

elseif ~isempty(regexp(lower(rvname), 'conn'))

start = datenum('20150824', 'yyyymmdd');

ender = datenum('20160825', 'yyyymmdd');

elseif ~isempty(regexp(lower(rvname), 'murder'))

start = datenum('20151001', 'yyyymmdd');

ender = datenum('20161002', 'yyyymmdd');

elseif ~isempty( regexp(strrep(rvname, '_',''), 'MID') ) % Middle River

start = datenum('20150701', 'yyyymmdd');

ender = datenum('20170702', 'yyyymmdd');

elseif ~isempty( regexp(strrep(rvname, '_',''), 'LIT') ) % Little Back - REMOVED

% - likely not enough data for a valid phase analysis - REMOVED

start = datenum('20170326', 'yyyymmdd');

ender = datenum('20170409', 'yyyymmdd');

elseif ~isempty( regexp(strrep(rvname, '_',''), 'SAV') ) % Savannah River

start = datenum('20151005', 'yyyymmdd');

ender = datenum('20161006', 'yyyymmdd');

elseif ~isempty( regexp(strrep(rvname, '_',''), 'OGE') ) % Ogeechee River

start = datenum('20150701', 'yyyymmdd');

ender = datenum('20170702', 'yyyymmdd');

elseif ~isempty( regexp(strrep(rvname, '_',''), 'STM') ) % St Mary's River

start = datenum('20150701', 'yyyymmdd');

ender = datenum('20160701', 'yyyymmdd');

elseif ~isempty( regexp(strrep(rvname, '_',''), 'CHA') ) % Chassahowitza River

% - likely not enough data for a valid phase analysis

start = datenum('20160702', 'yyyymmdd'); % '20161115'

ender = datenum('20170703', 'yyyymmdd');

elseif ~isempty( regexp(strrep(rvname, '_',''), 'HAL') ) % Halls River

% - likely not enough data for a valid phase analysis

start = datenum('20160701', 'yyyymmdd');

ender = datenum('20170702', 'yyyymmdd');

elseif ~isempty( regexp(strrep(rvname, '_',''), 'CRY') ) % Crystal River

start = datenum('20150825', 'yyyymmdd');

ender = datenum('20160826', 'yyyymmdd');

elseif ~isempty( regexp(strrep(rvname, '_',''), 'MOB') ) % Mobile River

% - strong seasonaility that may obscur (semi-)diurnal signal

start = datenum('20150701', 'yyyymmdd');

ender = datenum('20170702', 'yyyymmdd');

elseif ~isempty( regexp(strrep(rvname, '_',''), 'WAX') ) % Wax Lake - REMOVED

% - strong/odd (semi-)annual cycle, invalid phase analysis - REMOVED

start = datenum('20150701', 'yyyymmdd');

ender = datenum('20170702', 'yyyymmdd');

% Columbia Slough- BAD DATA!!

% EAST FORK - BAD DATA!!

% Little Back - REMOVED

% Wax Lake - REMOVED

else

start = [];

ender = [];

end

%% after having selected the proper time periods for an FFT, perform the FFT

% % remove odd timing in raw data

% idx = find(diff(data(:,1)) < min15/4);

% data(idx,:) = [];

% linearly interpolate "data" onto 15 minute intervals

% closing any data gaps...

timeline = [start:min15:ender]';

idata = nan( length(timeline), size(data,2)+1 );

idata(:,1) = timeline;

idx = find( ~isnan(data(:,2)) );

idata(:,2) = interp1q(data(idx,1), data(idx,2), timeline);

idx = find( ~isnan(data(:,3)) );

idata(:,3) = interp1q(data(idx,1), data(idx,3), timeline);

dldt = [(idata(2:end,3) - idata(1:end-1,3))./...

(idata(2:end,1) - idata(1:end-1,1)); nan];

idata(:,4) = dldt;

% performing a moving average on the data

fftavg = nan( size(idata) );

fftavg(:,1) = aej_moving_avg( idata(:,1), fftwindow );

fftavg(:,2) = aej_moving_avg( idata(:,2), fftwindow );

fftavg(:,3) = aej_moving_avg( idata(:,3), fftwindow );

fftavg(:,4) = aej_moving_avg( idata(:,4), fftwindow );

[phi_value, mthm] = FXN_USGS_phase( save_path, save_fig,...

'USGS_FFTphase\',...

fftavg(:,1), fftavg(:,2), fftavg(:,3), rvname );

%% writing phase offset data

wave_type = 'Mixed';

if write_save ~= 0

if phi_value*(180/pi) <= 95 & phi_value*(180/pi) >= 85

wave_type = 'Standing';

elseif phi_value*(180/pi) <= 185 & phi_value*(180/pi) >=175

wave_type = 'Progressive';

elseif phi_value*(180/pi) <= 85

wave_type = '*Standing';

end

labeler = strrep(rvname, '_', '');

fprintf(labelcsv, '%s, %s, %s, %.4f, %s\r\n',...

strrep(rvname, '_', ' '),...

sitenum,...

labeler(1:3),...

phi_value*(180/pi),...

wave_type);

end

%% Plot phase offset

% if ~isempty(regexp(lower(rvname), 'plum')) ||...

% ~isempty(regexp(lower(rvname), 'shark')) ||...

% ~isempty(regexp(lower(rvname), 'conn')) ||...

% ~isempty(regexp(lower(rvname), 'murder'))

% marker = 'p';

% ms = 8;

% else

% marker = '.';

% ms = 15;

% end

figure(1e5)

plot(mthm, phi_value,...

'k', 'markersize', 15, 'marker', '.')

hold on

labeler = strrep(rvname, '_', '');

text(mthm, phi_value,...

upper(labeler(1:3)),...

'VerticalAlignment', 'bottom',...

'HorizontalAlignment','center',...

'color', 'k')

end

%% add plot fixings

figure(1e5)

% plot labels

plot(get(gca, 'xlim'), [(95/180)*pi, (95/180)*pi], '--', 'color', [0.5,0.5,0.5])

plot(get(gca, 'xlim'), [(85/180)*pi, (85/180)*pi], '--', 'color', [0.5,0.5,0.5])

plot(get(gca, 'xlim'), [(175/180)*pi, (175/180)*pi], '--', 'color', [0.5,0.5,0.5])

plot(get(gca, 'xlim'), [(185/180)*pi, (185/180)*pi], '--', 'color', [0.5,0.5,0.5])

ylabel({'Avg. Phase Offset, (\phi)', '[degrees]'})

% Labeling "Wave Types"

xxx = get(gca, 'xlim');

text(xxx(1), pi/2, ['Standing'])

text(xxx(1), 3*pi/4, ['Mixed'])

text(xxx(1), pi, ['Progressive'])

set(gca, 'ylim', [pi/4, 5*pi/4])

set(gca, 'ytick', [0, pi/4, pi/2, (3/4)*pi, pi,...

(5/4)*pi, (3/2)*pi, (7/4)*pi, 2*pi])

set(gca, 'yticklabel', {'0', '45', '90', '135', '180',...

'225', '270','315', '360'})

% x-axis

xlabel({'Maximum Tidal', 'Harmonic Magnitude'})

set(gca, 'xscale', 'log')

% saving figures of all major axes

if save_fig ~= 0;

% *** SAVING FIGURE BEFORE MOVING ON

% makes sure the saved pdf fits within a landscape document

set(gcf,'PaperOrientation','landscape');

set(gcf,'PaperUnits','normalized');

set(gcf,'PaperPosition', [0 0 1 1]);

% save as .tif

print(gcf, '-dpdf' , [save_path 'all_phaseOFFSET_oneplot.pdf'])

end

%% close .csv file

if write_save ~= 0

fclose(labelcsv);

end

%% empirically determine best times for analysis

% close all

% rvname

% plotyy(data(:,1), data(:,2), data(:,1), data(:,3))

% set(gca, 'xticklabel', datestr(get(gca, 'xtick'),'mm-dd-yy'))

======== END FILE ========

File 2: The following function file computes a moving average.

======== BEGIN FILE ========

function [movavg] = aej_moving_avg(input_data, window_size)

%{

A function that returns a moving average that is the same length as the

incoming data.

Requires a numeric 'window size' to average over.

The function returns a column of data that the moving average has taken

place upon.

Author: Allan Jones

Date: 02/22/2017

%}

movavg = [];

for iii = 1:length(input_data)

% recording nans - no bias at ends, but same length array

if iii < window_size/2 || iii + floor(window_size/2) > length(input_data)

movavg = [movavg; nan];

% normal averaging window for majority of cells

else

movavg = [movavg;...

mean(input_data(iii-floor(window_size/2):iii+floor(window_size/2)))];

end

end

% return the data

return

======== END FILE ========

File 3: The following function file obtains the discharge and stage data from downloaded textfiles from the USGS NWIS.

======== BEGIN FILE ========

function [rv_data] = obtain_rv_data(river_file_path, river_name, site_number)

%{

The purpose of this function is to tease through the river .txt file and

obtain the discharge and stage data. The river .txt files must be downloaded

via the python script (proper setup). This function returns a cell matrix

composed of {datetime [converted]}.

You need to supply the file path to the river file you want downloaded.

Author: Allan Jones

Date: October 8, 2014

%}

%% Input parser

p = inputParser;

p.FunctionName = 'obtain_rv_data';

% Required variables

addRequired(p, 'river_file_path', @isstr);

addRequired(p, 'river_name', @isstr);

addRequired(p, 'site_number', @isstr);

% Parsing variables

parse(p, river_file_path, river_name, site_number);

rv_path = p.Results.river_file_path;

rv_name = p.Results.river_name;

site_num = p.Results.site_number;

%% Retrieve the data from the file

rv_fid = fopen([rv_path], 'r');

% Settiing up the counter and starting line variables

start_line = [];

idum = 1;

max_len = 0;

% Reading the first line of the file

file_lines{idum,1} = fgets(rv_fid);

% WHILE loops through and records the file's lines

% and finds the start of the data

while ~isempty(file_lines{idum,1})

idum = idum + 1;

% Reads the next line

file_lines{idum,1} = fgets(rv_fid);

% Breaks the file after the last line of data

if ~ischar(file_lines{idum,1})

break

% Finds and records the starting line of the data

elseif ischar(file_lines{idum,1}) &&...

isempty(regexp(file_lines{idum,1},'#')) && ...

~isempty(regexp(file_lines{idum,1},'USGS')) && ...

~isempty(regexp(file_lines{idum,1},site_num)) && ...

isempty(start_line)

start_line = idum;

% Finds the order of the variables (stage v discharge) displayed in file

%{

elseif ~isempty(regexp(lower(file_lines{idum,1}),'gag')) &&...

isempty(start_line)

g_line = idum;

elseif ~isempty(regexp(lower(file_lines{idum,1}),'dis')) &&...

isempty(start_line)

d_line = idum;

end

%}

elseif ~isempty(regexp(file_lines{idum,1},...

['# Data provided for site ' site_num]))

dummy = fgets(rv_fid); % gets variable header line

aaa = 1;

while length(dummy) > 3

dummy = fgets(rv_fid); % gets first variable line

if ~isempty(regexp(dummy, 'Gage'))

gage_col = aaa;

elseif ~isempty(regexp(dummy, 'Discharge'))

dis_col = aaa;

end

aaa = aaa + 1;

end

end

if length(file_lines{idum,1}) > max_len && ~isempty(start_line)

max_len = length(file_lines{idum,1});

end

end

fclose(rv_fid);

% Regaining memory

clear rv_fid

% Setting order for variables displayed in file

%{

if g_line < d_line

var_order = {'stage' 'discharge'};

else

var_order = {'discharge' 'stage'};

end

%}

% Separates the actual data from the remainder and creates matrix

rv_rawdata = file_lines(start_line:end-1,1);

% Finds indices within each string '-', ':', A, P, or T which separate data

commas = regexp(rv_rawdata, ',');

% hyphen = regexp(rv_rawdata, '-');

% colon = regexp(rv_rawdata, ':');

% AAA = regexp(rv_rawdata, 'A');

% PPP = regexp(rv_rawdata, 'P');

% TTT = regexp(rv_rawdata, 'T');

% for loop that creates discharge, stage and elapsed cell martices

% need a series of if statements for 1 or 2 a's and 1 or 2 p's

dis_col = 4 + dis_col + (dis_col-1);

gage_col = 4 + gage_col + (gage_col-1); % adding datetime columns

for iii = 1:length(rv_rawdata)

% Pulling date from string

datetime{iii,1} = rv_rawdata{iii}(commas{iii,1}(2)+1:commas{iii,1}(3)-1);

% obtaining the discharge and stage data

stage{iii,1} = rv_rawdata{iii}(commas{iii,1}(gage_col-1)+1:commas{iii,1}(gage_col)-1);

discharge{iii,1} = rv_rawdata{iii}(commas{iii,1}(dis_col-1)+1:commas{iii,1}(dis_col)-1);

end

% Regaining memory

clear hyphen colon AAA PPP TTT

% use str2double and datenum on the completed cell arrays

elapsed = datenum(datetime, 'yyyy-mm-dd HH:MM');

stage = str2double(stage);

discharge = str2double(discharge);

% converting the variables to metric units

stage = stage.*.3048; %convert stage from ft to meters

discharge = discharge.*(.3048^3); %convert discharge from cubic feet/sec to cubic meters/sec

converted = [elapsed discharge stage];

% double check the lengths of datetime and converted matrices

% if they are not equal, report an error

if length(datetime) ~= length(converted)

error(['ERROR: The lengths of the ''datetime'' and river data '...

'vectors are NOT the same.'])

end

% The cell matrix to be returned containing {datetime [converted]}

rv_data = {datetime converted};

end

======== END FILE ========

File 4: The following file performs the baseflow tidal rating curve analysis and saves the resulting coefficients, k_1_-k_4_.

======== BEGIN FILE ========

%{

The baseflow tidal rating curve analysis. Simplified script.

Author: Allan Jones

Date: 6 September 2017

%}

clear; clc; close all

% determining which computer is being used

cur_dir = cd; slashes = regexp(cur_dir, '\');

comp_using = cur_dir(1:slashes(3));

save_fig = 0;

write_save = 0;

fig = 1;

addnotes = '';

%% SAVE INFO

save_folder = [date '_plots\'];

save_path = [comp_using '\Dropbox\Field Work\Analysis\'...

'Field Data Analysis\TCM rating curve\Figures\' save_folder];

if ~isdir([save_path])

mkdir([save_path]);

end

% write .csv files

if write_save ~= 0

csvpath = save_path;

% [comp_using 'Dropbox\Field Work\Analysis\'...

% 'Field Data Analysis\TCM rating curve\MA Regression Info\'];

kscsv = fopen([csvpath 'tidalratingcurve_coefficents.csv'],'w');

% write headers

fprintf(kscsv, ['Site Label,'...

'k1 +/- SE (tStat; p), k2 +/- SE (tStat; p), '...

'k3 +/- SE (tStat; p), k4 +/- SE (tStat; p), Rsquared\r\n']);

end

%% Loading all the calibrated TCM data

% creating final_master (combining all deployments) from site data

disp(['Loading calibrated TCM data and performing moving average...'])

soi = {'A1', 'A2', 'A3', 'A4', 'A5','M1', 'M2', 'M3', 'M4', 'M5'};

min15 = 15/(24*60);

%%%%%%%%%%%%%%%%% - ARANSAS RIVER

% site cleaning thresholds - [qlow, qhi, slow, shi]

sites.A1.thresholds = [-35, 250, 1.10, 2.7];

sites.A2.thresholds = [-35, 60, 1.10, 2.7];

sites.A3.thresholds = [-50, 90, 1.75, 3.1];

sites.A4.thresholds = [-50, 60, 1.75, 3.25];

sites.A5.thresholds = [-50, 40, 2.2, 3.75];

% site start and end timestamps

sites.A1.times = [datenum('20150701 00:00', 'yyyymmdd HH:MM'),...

datenum('20170701 00:00', 'yyyymmdd HH:MM')];

sites.A2.times = [datenum('20150701 00:00', 'yyyymmdd HH:MM'),...

datenum('20160701 00:00', 'yyyymmdd HH:MM')];

sites.A3.times = [datenum('20150701 00:00', 'yyyymmdd HH:MM'),...

datenum('20160701 00:00', 'yyyymmdd HH:MM')];

sites.A4.times = [datenum('20150701 00:00', 'yyyymmdd HH:MM'),...

datenum('20170701 00:00', 'yyyymmdd HH:MM')];

sites.A5.times = [datenum('20150701 00:00', 'yyyymmdd HH:MM'),...

datenum('20160701 00:00', 'yyyymmdd HH:MM')];

%%%%%%%%%%%%%%%%% - MISSION RIVER

% site cleaning thresholds - [qlow, qhi, slow, shi]

sites.M1.thresholds = [-35, 250, 0.75, 5.5];

sites.M2.thresholds = [-35, 250, 0.75, 5.5];

sites.M3.thresholds = [-20, 90, 1.00, 5.5];

sites.M4.thresholds = [-30, 90, 1.25, 5.5];

sites.M5.thresholds = [-50, 40, 2.2, 5.5];

% site start and end timestamps

sites.M1.times = [datenum('20160720 00:00', 'yyyymmdd HH:MM'),...

datenum('20170710 00:00', 'yyyymmdd HH:MM')];

sites.M2.times = [datenum('20160303 00:00', 'yyyymmdd HH:MM'),...

datenum('20160710 00:00', 'yyyymmdd HH:MM')];

sites.M3.times = [datenum('20150701 00:00', 'yyyymmdd HH:MM'),...

datenum('20160701 00:00', 'yyyymmdd HH:MM')];

sites.M4.times = [datenum('20150701 00:00', 'yyyymmdd HH:MM'),...

datenum('20160701 00:00', 'yyyymmdd HH:MM')];

sites.M5.times = [datenum('20150625 00:00', 'yyyymmdd HH:MM'),...

datenum('20160104 00:00', 'yyyymmdd HH:MM')];

%% Gathering all ADP data

disp(['Gathering all ADP data and storing record of ADP transects...'])

ADP_dat = gather_ADP_data();

%% Record of ADP transects

aranADPtimes = ... {datenums (bracketing AD obs), site str; repeat};

{...[datenum('10-Aug-2015'), datenum('12-Aug-2015')], ['Ahr24'];... ['Ahr24'] or ['A2']?

...[datenum('27-Feb-2016'), datenum('29-Feb-2016')], ['Ahr24'];... ['Ahr24'] or ['A2']?

...STORM CONDITIONS/(not baseflow) [datenum('201506220845', 'yyyymmddHHMM'), datenum('201506221015', 'yyyymmddHHMM')], ['A1'];...

...STORM CONDITIONS/(not baseflow) [datenum('201506221000', 'yyyymmddHHMM'), datenum('201506221115', 'yyyymmddHHMM')], ['A3'];...

...STORM CONDITIONS/(not baseflow) [datenum('201506221100', 'yyyymmddHHMM'), datenum('201506221215', 'yyyymmddHHMM')], ['A4'];...

[datenum('201508081200', 'yyyymmddHHMM'), datenum('201508081400', 'yyyymmddHHMM')], ['A3'];...

[datenum('201508081415', 'yyyymmddHHMM'), datenum('201508081545', 'yyyymmddHHMM')], ['A4'];...

[datenum('201601111100', 'yyyymmddHHMM'), datenum('201601111230', 'yyyymmddHHMM')], ['A4'];...

[datenum('201601111245', 'yyyymmddHHMM'), datenum('201601111400', 'yyyymmddHHMM')], ['A3'];...

[datenum('201601111530', 'yyyymmddHHMM'), datenum('201601111645', 'yyyymmddHHMM')], ['A1'];...

[datenum('201606150900', 'yyyymmddHHMM'), datenum('201606151300', 'yyyymmddHHMM')], ['A4'];...

[datenum('201607161145', 'yyyymmddHHMM'), datenum('201607161415', 'yyyymmddHHMM')], ['A4'];...

[datenum('201612010830', 'yyyymmddHHMM'), datenum('201612011045', 'yyyymmddHHMM')], ['A4'];...

[datenum('201612011045', 'yyyymmddHHMM'), datenum('201612011245', 'yyyymmddHHMM')], ['A3'];...

[datenum('201612011230', 'yyyymmddHHMM'), datenum('201612011420', 'yyyymmddHHMM')], ['A2'];...

[datenum('201612011415', 'yyyymmddHHMM'), datenum('201612011600', 'yyyymmddHHMM')], ['A1'];...

[datenum('201612020900', 'yyyymmddHHMM'), datenum('201612021115', 'yyyymmddHHMM')], ['A1'];...

[datenum('201612021130', 'yyyymmddHHMM'), datenum('201612021315', 'yyyymmddHHMM')], ['A2'];...

[datenum('201612021300', 'yyyymmddHHMM'), datenum('201612021515', 'yyyymmddHHMM')], ['A3'];...

[datenum('201612021445', 'yyyymmddHHMM'), datenum('201612021645', 'yyyymmddHHMM')], ['A4'];...

[datenum('201701100800', 'yyyymmddHHMM'), datenum('201701101000', 'yyyymmddHHMM')], ['A4'];...

[datenum('201701100945', 'yyyymmddHHMM'), datenum('201701101120', 'yyyymmddHHMM')], ['A3'];...

[datenum('201701101145', 'yyyymmddHHMM'), datenum('201701101330', 'yyyymmddHHMM')], ['A2'];...

[datenum('201701101315', 'yyyymmddHHMM'), datenum('201701101500', 'yyyymmddHHMM')], ['A1'];...

[datenum('201702020845', 'yyyymmddHHMM'), datenum('201702021030', 'yyyymmddHHMM')], ['A4'];...

[datenum('201702021030', 'yyyymmddHHMM'), datenum('201702021200', 'yyyymmddHHMM')], ['A3'];...

[datenum('201702021215', 'yyyymmddHHMM'), datenum('201702021345', 'yyyymmddHHMM')], ['A2'];...

[datenum('201702021400', 'yyyymmddHHMM'), datenum('201702021545', 'yyyymmddHHMM')], ['A1'];...

};

% if the interpolation method works, need to extend the mission river adp

% times by 30 minutes like we did with the Aransas (15 min before and

% after)

missADPtimes = ... {datenums (bracketing ADP obs), site str; repeat};

{...STORM CONDITIONS/(not baseflow) [datenum('201506180930', 'yyyymmddHHMM'), datenum('201506181100', 'yyyymmddHHMM')], ['M1'];...

...STORM CONDITIONS/(not baseflow) [datenum('201506181115', 'yyyymmddHHMM'), datenum('201506181200', 'yyyymmddHHMM')], ['M3'];...

...STORM CONDITIONS/(not baseflow) [datenum('201506181230', 'yyyymmddHHMM'), datenum('201506181345', 'yyyymmddHHMM')], ['M5'];...

[datenum('201508140915', 'yyyymmddHHMM'), datenum('201508141115', 'yyyymmddHHMM')], ['M5'];...

[datenum('201508141200', 'yyyymmddHHMM'), datenum('201508141450', 'yyyymmddHHMM')], ['M3'];...

[datenum('201508141330', 'yyyymmddHHMM'), datenum('201508141500', 'yyyymmddHHMM')], ['M2'];...

[datenum('201601121030', 'yyyymmddHHMM'), datenum('201601121145', 'yyyymmddHHMM')], ['M5'];...

[datenum('201601121340', 'yyyymmddHHMM'), datenum('201601121515', 'yyyymmddHHMM')], ['M2'];...

[datenum('201606160845', 'yyyymmddHHMM'), datenum('201606161245', 'yyyymmddHHMM')], ['M5'];...

[datenum('201607171045', 'yyyymmddHHMM'), datenum('201607171345', 'yyyymmddHHMM')], ['M2'];...

[datenum('201701110915', 'yyyymmddHHMM'), datenum('201701111100', 'yyyymmddHHMM')], ['M4'];...

[datenum('201701111115', 'yyyymmddHHMM'), datenum('201701111300', 'yyyymmddHHMM')], ['M3'];...

[datenum('201701111315', 'yyyymmddHHMM'), datenum('201701111500', 'yyyymmddHHMM')], ['M1'];...

[datenum('201702030900', 'yyyymmddHHMM'), datenum('201702031100', 'yyyymmddHHMM')], ['M4'];...

[datenum('201702031100', 'yyyymmddHHMM'), datenum('201702031300', 'yyyymmddHHMM')], ['M3'];...

[datenum('201702031300', 'yyyymmddHHMM'), datenum('201702031500', 'yyyymmddHHMM')], ['M1'];...

};

%% Beginning M-A Analysis

% create a path for loading data

load_path = ['Dropbox\Field Work\Analysis\All Long Term Data\'...

'Matlab Cleaned Data\Calibrated TCM data\'];

data_saving = 'prelim_20170725\TCM matrix-only data\';

window_size = 13; % moving average window -> best = 13 (dldt + stage - 1)

% 9 - +/- 1 hour

% 13 - +/- 1.5 hours

% 17 - +/- 2 hours

% 25 - +/- 3 hours

% 51 - +/- 6 hours -> 12 hours total

counter = 1;

counter2 = 1;

% load each site's data and create a final_master matrix from deployments

% determine dl/dt for each site's data

lroptions = {'Q ~ dldt^2 + dldt + stage^2 + stage - 1',...

'Q ~ dldt^2 + dldt + stage^2 - stage - 1',...

'Q ~ dldt^2 + dldt - 1',...

'Q ~ dldt^2 - dldt - 1',...

'Q ~ dldt + stage^2 + stage - 1',...

'Q ~ dldt + stage^2 - stage - 1',...

'Q ~ dldt - 1',...

'Q ~ stage^2 + stage + dldt^2 - dldt - 1',...

'Q ~ stage^2 + stage - 1',...

'Q ~ stage^2 - stage - 1',...

'Q ~ stage + dldt^2 + dldt - 1',...

'Q ~ stage + dldt^2 - dldt - 1',...

'Q ~ stage - 1',...

'Q ~ stage + dldt - 1',...

'Q ~ dldt^2 - dldt + stage^2 - stage - 1'};

for vvv = 1:length(soi) % [1, 3, 4] % 4 % 1:4 % [1, 3, 4] %

disp(['Loading site: ' soi{vvv}])

% loading saved TCM/LTC data

eval(['load([comp_using load_path data_saving '...

'soi{vvv} '...

'''_calib_mat_only.mat''])'])

% Making data ONE LARGE data variable

eval(['data = ' soi{vvv} '_mat_only;'])

all_data = [nanmean([data(:,1), data(:,5)],2),... % averaged LTC and TCM timestamps

data(:,17),... % TCM velocities [cm s-1]

data(:,2)]; % LTC stage [m]

% remove bizarre points

eval(['threshs = sites.' soi{vvv} '.thresholds;'])

% velocities < -50 cm/s || > 90 cm/s

idx = find( (all_data(:,2) < threshs(1)) | (all_data(:,2) > threshs(2)) );

all_data(idx, :) = [];

% stages

idx = find( (all_data(:,3) < threshs(3)) | (all_data(:,3) > threshs(4)) );

all_data(idx, :) = [];

% obtaining a movin average of raw (uninterpolated) data

avgdata = nan( length(all_data), size(all_data,2)+1 );

avgdata(:,1) = aej_moving_avg(all_data(:,1), window_size);

avgdata(:,2) = aej_moving_avg(all_data(:,2), window_size);

avgdata(:,3) = aej_moving_avg(all_data(:,3), window_size);

dldt = [(all_data(2:end,3) - all_data(1:end-1,3))./...

(all_data(2:end,1) - all_data(1:end-1,1)); nan];

avgdata(:,4) = aej_moving_avg(dldt, window_size);

%% Interpolate stage, dldt, and velocity data to ADP

% create a variable to store [time, Q, q, stage, dldt]

if strcmp( soi{vvv}(1), 'A')

adptimes = aranADPtimes;

else

adptimes = missADPtimes;

end

% loop through 'adptimes' to find indices of given site's ADP transects

idx = [];

for iii = 1:length(adptimes)

if strcmp(adptimes{iii,2}, soi{vvv})

idx = [idx; iii];

end

end

% loop through indices and obtain the data between timestamps

data2Q = [];

for iii = 1:length(idx)

jjj = idx(iii);

% obtaining ADP timestamps

hrs = adptimes{jjj,1}(1); hrsend = adptimes{jjj,1}(2);

% finding indices

idat = find( (avgdata(:,1) >= hrs) & (avgdata(:,1) < hrsend) );

iADP = find( (ADP_dat(:,1) >= hrs) & (ADP_dat(:,1) < hrsend) );

% interpolate the velocity, stage and dldt to Q timestamps

if ~isempty(idat)

% interpolate TCM velocity data

interp_TCM = interp1q(avgdata(idat,1),... % site timeline

avgdata(idat,2)./100,... % TCM q, converted from cm/s to m/s

ADP_dat(iADP,1)); % "new" ADP timeline

% interpolate stage data

interp_stage = interp1q(avgdata(idat,1),... % site timeline

avgdata(idat,3),... % stage [m]

ADP_dat(iADP,1)); % "new" ADP timeline

% interpolate dldt data

interp_dldt = interp1q(avgdata(idat,1),... % site tiplot(meline

avgdata(idat,4),... % dl/dt

ADP_dat(iADP,1)); % "new" ADP timeline

else

disp(['No site data found for the following date: ' datestr(hrs)])

interp_TCM = nan(length(iADP),1);

interp_stage = nan(length(iADP),1);

interp_dldt = nan(length(iADP),1);

end

% store interpolated data into storage variable

holder = [ADP_dat(iADP,1),... % ADP time

ADP_dat(iADP,2),... % ADP Q

interp_TCM,... % TCM velocity [m/s]

interp_stage,... % stage [m]

interp_dldt,... % dldt

];

% removing velocities that are "too slow" to accurately resolve:

% (< |2 cm/s| )

less2cm = find( abs(holder(:,3)) <= (2/100) );

holder(less2cm,3) = 0;

kkk = find(isnan(holder(:,3)));

holder(kkk,3)= 0;

% finally store the data in a storage variable

data2Q = [data2Q; holder];

end

%% perform linear regression to ADP Q - multiple tests

%7{

% options:

% ADPQ ~ dldt + stage + TCMq - 1

% ADPQ ~ dldt + TCMq - 1

% ADPQ ~ dldt - 1

% ADPQ ~ TCMq - 1

% initializing variable that stores the "best data"

best = [];

bestr2 = -100.00; % adjusted rsquared

if ~isempty(data2Q)

% % normaizing stage - 0 to 1

% data2Q(:,2) = [(data2Q(:,2) - min(data2Q(:,2)))./...

% (max(data2Q(:,2)) - min(data2Q(:,2)))];l

% creating linear regression table

tbl = table(data2Q(:,1), data2Q(:,2), data2Q(:,3),...

data2Q(:,4),... data2Q(:,4).*abs(cos(mean(phase_offset))),...

data2Q(:,5),... data2Q(:,5).*abs(sin(mean(phase_offset))),...

'VariableNames',...

{'time', 'Q', 'TCMq', 'stage', 'dldt'});

plot_notes = ['']; % ['** Does not include q < 2 cm s^{-1}']

idx = 1;

for ooo = 1:length(lroptions)

% plotting the site relationship

adp_lm = fitlm(tbl, lroptions{ooo});

% storing the Linear Model fits data

if all(adp_lm.Coefficients.pValue <= 0.05) &... p < 0.05 and...

adp_lm.Rsquared.Adjusted > bestr2 % and new r^2 > old r^2

% resetting the "best" variables

best.k1 = zeros(1,4);

best.k2 = zeros(1,4);

best.k3 = zeros(1,4);

best.k4 = zeros(1,4);

for aaa = 1:length(adp_lm.CoefficientNames)

if strcmp(adp_lm.CoefficientNames{aaa}, 'stage^2')

best.k1 = table2array(adp_lm.Coefficients(aaa,:));

elseif strcmp(adp_lm.CoefficientNames{aaa}, 'stage')

best.k2 = table2array(adp_lm.Coefficients(aaa,:));

elseif strcmp(adp_lm.CoefficientNames{aaa}, 'dldt^2')

best.k3 = table2array(adp_lm.Coefficients(aaa,:));

elseif strcmp(adp_lm.CoefficientNames{aaa}, 'dldt')

best.k4 = table2array(adp_lm.Coefficients(aaa,:));

end

end

bestr2 = adp_lm.Rsquared.Adjusted;

idx = ooo;

end

end

% plotting "best" fit

adp_lm = fitlm(tbl, lroptions{idx})

figure(fig)

% makes sure the saved pdf fits within a landscape document

set(gcf,'PaperOrientation','landscape');

set(gcf,'PaperUnits','normalized');

set(gcf,'PaperPosition', [0 0 1 1]);

% plot linear model

plot(adp_lm)

xticks = get(gca, 'xtick');

yticks = get(gca, 'ytick');

title([soi{vvv} ': ' lroptions{idx}])

grid on

legend off

num_factors = length(regexp(adp_lm.Formula.LinearPredictor, '+'))+1;

if num_factors == 1

text(mean(xticks), yticks(2), {...

[num2str(adp_lm.Coefficients.Estimate(1)) '*' adp_lm.CoefficientNames{1}];...

['Adjusted R^2 = ' num2str(adp_lm.Rsquared.Adjusted)];...

['Ordinary R^2 = ' num2str(adp_lm.Rsquared.Ordinary)];...

['p-value: ' adp_lm.CoefficientNames{1} ' = ' num2str(adp_lm.Coefficients.pValue(1))];...

plot_notes...

}, 'fontsize', 8)

elseif num_factors == 2

text(mean(xticks), yticks(2), {...

[num2str(adp_lm.Coefficients.Estimate(1)) '*' adp_lm.CoefficientNames{1}];...

[num2str(adp_lm.Coefficients.Estimate(2)) '*' adp_lm.CoefficientNames{2}];...

['Adjusted R^2 = ' num2str(adp_lm.Rsquared.Adjusted)];...

['Ordinary R^2 = ' num2str(adp_lm.Rsquared.Ordinary)];...

['p-value: ' adp_lm.CoefficientNames{1} ' = ' num2str(adp_lm.Coefficients.pValue(1))];...

['p-value: ' adp_lm.CoefficientNames{2} ' = ' num2str(adp_lm.Coefficients.pValue(2))];...

plot_notes...

}, 'fontsize', 8)

elseif num_factors == 3

text(mean(xticks), yticks(2), {...

[num2str(adp_lm.Coefficients.Estimate(1)) '*' adp_lm.CoefficientNames{1}];...

[num2str(adp_lm.Coefficients.Estimate(2)) '*' adp_lm.CoefficientNames{2}];...

[num2str(adp_lm.Coefficients.Estimate(3)) '*' adp_lm.CoefficientNames{3}];...

['Adjusted R^2 = ' num2str(adp_lm.Rsquared.Adjusted)];...

['Ordinary R^2 = ' num2str(adp_lm.Rsquared.Ordinary)];...

['p-value: ' adp_lm.CoefficientNames{1} ' = ' num2str(adp_lm.Coefficients.pValue(1))];...

['p-value: ' adp_lm.CoefficientNames{2} ' = ' num2str(adp_lm.Coefficients.pValue(2))];...

['p-value: ' adp_lm.CoefficientNames{3} ' = ' num2str(adp_lm.Coefficients.pValue(3))];...

plot_notes...

}, 'fontsize', 8)

elseif num_factors == 4

text(mean(xticks), yticks(2), {...

[num2str(adp_lm.Coefficients.Estimate(1)) '*' adp_lm.CoefficientNames{1}];...

[num2str(adp_lm.Coefficients.Estimate(2)) '*' adp_lm.CoefficientNames{2}];...

[num2str(adp_lm.Coefficients.Estimate(3)) '*' adp_lm.CoefficientNames{3}];...

[num2str(adp_lm.Coefficients.Estimate(4)) '*' adp_lm.CoefficientNames{4}];...

['Adjusted R^2 = ' num2str(adp_lm.Rsquared.Adjusted)];...

['Ordinary R^2 = ' num2str(adp_lm.Rsquared.Ordinary)];...

['p-value: ' adp_lm.CoefficientNames{1} ' = ' num2str(adp_lm.Coefficients.pValue(1))];...

['p-value: ' adp_lm.CoefficientNames{2} ' = ' num2str(adp_lm.Coefficients.pValue(2))];...

['p-value: ' adp_lm.CoefficientNames{3} ' = ' num2str(adp_lm.Coefficients.pValue(3))];...

['p-value: ' adp_lm.CoefficientNames{4} ' = ' num2str(adp_lm.Coefficients.pValue(4))];...

plot_notes...

}, 'fontsize', 8)

elseif num_factors == 4

text(mean(xticks), yticks(2), {...

[num2str(adp_lm.Coefficients.Estimate(1)) '*' adp_lm.CoefficientNames{1}];...

[num2str(adp_lm.Coefficients.Estimate(2)) '*' adp_lm.CoefficientNames{2}];...

[num2str(adp_lm.Coefficients.Estimate(3)) '*' adp_lm.CoefficientNames{3}];...

[num2str(adp_lm.Coefficients.Estimate(4)) '*' adp_lm.CoefficientNames{4}];...

['Adjusted R^2 = ' num2str(adp_lm.Rsquared.Adjusted)];...

['Ordinary R^2 = ' num2str(adp_lm.Rsquared.Ordinary)];...

['p-value: ' adp_lm.CoefficientNames{1} ' = ' num2str(adp_lm.Coefficients.pValue(1))];...

['p-value: ' adp_lm.CoefficientNames{2} ' = ' num2str(adp_lm.Coefficients.pValue(2))];...

['p-value: ' adp_lm.CoefficientNames{3} ' = ' num2str(adp_lm.Coefficients.pValue(3))];...

['p-value: ' adp_lm.CoefficientNames{4} ' = ' num2str(adp_lm.Coefficients.pValue(4))];...

plot_notes...

}, 'fontsize', 8)

end

% saving figures of all major axes

if save_fig ~= 0;

% *** SAVING FIGURE BEFORE MOVING ON

% save as .tif

% print(gcf, '-dpdf' , [save_path 'Qrelation_' soi{vvv} addnotes '.pdf'])

print(gcf, '-dtiff' , [save_path 'MA_Q_relate\Qrelation_' soi{vvv} addnotes '.tif'])

end

fig = fig +1;

end

%% Saving the data

%7{

% if not M2, M5 or A5

eval([soi{vvv} 'lr.data2Q = data2Q;'])

if ~strcmp(soi{vvv}, 'A5') && ~strcmp(soi{vvv}, 'M2')

eval([soi{vvv} 'lr.k_stage2 = best.k1(1);'])

eval([soi{vvv} 'lr.k_stage = best.k2(1);'])

eval([soi{vvv} 'lr.k_dldt2 = best.k3(1);'])

eval([soi{vvv} 'lr.k_dldt = best.k4(1);'])

else

eval([soi{vvv} 'lr.k_stage2 = nan;'])

eval([soi{vvv} 'lr.k_stage = nan;'])

eval([soi{vvv} 'lr.k_dldt2 = nan;'])

eval([soi{vvv} 'lr.k_dldt = nan;'])

end

eval(['save([save_path ''' soi{vvv} '_relate2Q.mat''], ''' soi{vvv} 'lr'')'])

%}

%% Writing a .CSV of the final

if write_save ~= 0

if strcmp(soi{vvv}, 'A5')

fprintf(kscsv, '%s, -*, -*, -*, -*, -*\r\n', soi{vvv});

elseif strcmp(soi{vvv}, 'M2') %|| strcmp(soi{vvv}, 'M5')

fprintf(kscsv, '%s, -**, -**, -**, -**, -**\r\n', soi{vvv});

elseif ~isempty(best) % %&& ~strcmp(soi{vvv}, 'M2')

fprintf(kscsv, ['%s,',...

'%.2f +/- %.2f (%.2f; %.2e),',...

'%.2f +/- %.2f (%.2f; %.2e),',...

'%.2f +/- %.2f (%.2f; %.2e),',...

'%.2f +/- %.2f (%.2f; %.2e),',...

'%.2f\r\n'],...

soi{vvv},...

best.k1,...

best.k2,...

best.k3,...

best.k4,...

bestr2);

else

fprintf(kscsv, '%s, nan, nan, nan, nan, nan\r\n', soi{vvv});

end

end

end

%% loading the USGS data

% path to the .txt files

USGSpath = [comp_using 'Dropbox\Field Work\Analysis\Field Data Analysis\'...

'TCM rating curve\USGS Tidal Sites\USGS Tidal Dnlds\TXT files'];

% obtaining the filenames from the

filenames = dir(USGSpath);

% cleaning odd structure fields

filenames(1) = []; filenames(1) = [];

for fff = 1:length(filenames) % 2 %

% obtaining sitenumber and rivername from filenames

underscore = regexp(filenames(fff).name, '_');

sitenum = filenames(fff).name(1:underscore(1)-1);

rvname = filenames(fff).name(underscore(1)+1:underscore(3)-1);

% update to user

disp(['Obtaining data for USGS ' sitenum ': ' strrep(rvname, '_', ' ')])

% obtaining the site data

data = obtain_rv_data([USGSpath '\' filenames(fff).name],...

rvname,...

sitenum);

data = data{2}; % keeping only [time, discharge, stage]

%% select two weeks of good data

if ~isempty(regexp(lower(rvname), 'plum'))

start = datenum('20151023', 'yyyymmdd');

ender = datenum('20151108', 'yyyymmdd');

elseif ~isempty(regexp(lower(rvname), 'shark'))

start = datenum('20160309', 'yyyymmdd');

ender = datenum('20160324', 'yyyymmdd');

elseif ~isempty(regexp(lower(rvname), 'conn'))

start = datenum('20150814', 'yyyymmdd');

ender = datenum('20150829', 'yyyymmdd');

elseif ~isempty(regexp(lower(rvname), 'murder'))

start = datenum('20160414', 'yyyymmdd');

ender = datenum('20160429', 'yyyymmdd');

data(:,3) = data(:,3) + 1.92*0.3048; % mean low-low water datum (1.92 ft)

elseif ~isempty( regexp(strrep(rvname, '_',''), 'MID') ) % Middle River

start = datenum('20160111', 'yyyymmdd');

ender = datenum('20160125', 'yyyymmdd');

elseif ~isempty( regexp(strrep(rvname, '_',''), 'LIT') ) % Little Back - REMOVED

% - likely not enough data for a valid phase analysis - REMOVED

start = datenum('20170326', 'yyyymmdd');

ender = datenum('20170409', 'yyyymmdd');

elseif ~isempty( regexp(strrep(rvname, '_',''), 'SAV') ) % Savannah River

start = datenum('20160420', 'yyyymmdd');

ender = datenum('20160505', 'yyyymmdd');

elseif ~isempty( regexp(strrep(rvname, '_',''), 'OGE') ) % OgeeChee River

start = datenum('20160428', 'yyyymmdd');

ender = datenum('20160513', 'yyyymmdd');

elseif ~isempty( regexp(strrep(rvname, '_',''), 'STM') ) % St Mary's River

start = datenum('20151220', 'yyyymmdd');

ender = datenum('20160104', 'yyyymmdd');

elseif ~isempty( regexp(strrep(rvname, '_',''), 'CHA') ) % Chassahowitza River

start = datenum('20150820', 'yyyymmdd');

ender = datenum('20150904', 'yyyymmdd');

elseif ~isempty( regexp(strrep(rvname, '_',''), 'HAL') ) % Halls River

start = datenum('20160627', 'yyyymmdd');

ender = datenum('20160711', 'yyyymmdd');

elseif ~isempty( regexp(strrep(rvname, '_',''), 'CRY') ) % Crystal River

start = datenum('20150907', 'yyyymmdd');

ender = datenum('20150922', 'yyyymmdd');

elseif ~isempty( regexp(strrep(rvname, '_',''), 'MOB') ) % Mobile River

% - likely not enough data for a valid phase analysis

start = datenum('20160810', 'yyyymmdd');

ender = datenum('20160901', 'yyyymmdd');

elseif ~isempty( regexp(strrep(rvname, '_',''), 'WAX') ) % Wax Lake - REMOVED

% - strong/odd (semi-)annual cycle, invalid phase analysis - REMOVED

start = datenum('20161027', 'yyyymmdd');

ender = datenum('20161110', 'yyyymmdd');

% Columbia Slough- BAD DATA!!

% EAST FORK - BAD DATA!!

% Little Back - REMOVED

% Wax Lake - REMOVED

else

start = [];

ender = [];

end

if ~isempty(start)

% perform moving average on data

idx = find( (data(:,1) >= start) & (data(:,1) < ender) );

clip_data = data(idx, :);

avgdata = nan(length(idx), 4);

avgdata(:,1) = aej_moving_avg(clip_data(:,1), window_size);

avgdata(:,2) = aej_moving_avg(clip_data(:,2), window_size);

avgdata(:,3) = aej_moving_avg(clip_data(:,3), window_size);

dldt = [(clip_data(2:end,3) - clip_data(1:end-1,3))./...

(clip_data(2:end,1) - clip_data(1:end-1,1)); nan];

avgdata(:,4) = aej_moving_avg(dldt, window_size);

% % normalizing stage - 0 to 1

% data2Q(:,2) = [(avgdata(:,2) - min(avgdata(:,2)))./...

% (max(avgdata(:,2)) - min(avgdata(:,2)))];

% create the table

tbl = table(avgdata(:,1), avgdata(:,2), avgdata(:,3), avgdata(:,4),...

'VariableNames', {'time', 'Q', 'stage', 'dldt'});

% initializing variable that stores the "best data"

best = [];

bestr2 = -100.00; % adjusted rsquared

idx = 1;

for ooo = 1:length(lroptions)

% plotting the site relationship

adp_lm = fitlm(tbl, lroptions{ooo});

% storing the Linear Model fits data

if all(adp_lm.Coefficients.pValue <= 0.05) &... p < 0.05 and...

adp_lm.Rsquared.Adjusted > bestr2 % and new r^2 > old r^2

% resetting the "best" variables

best.k1 = zeros(1,4);

best.k2 = zeros(1,4);

best.k3 = zeros(1,4);

best.k4 = zeros(1,4);

for aaa = 1:length(adp_lm.CoefficientNames)

if strcmp(adp_lm.CoefficientNames{aaa}, 'stage^2')

best.k1 = table2array(adp_lm.Coefficients(aaa,:));

elseif strcmp(adp_lm.CoefficientNames{aaa}, 'stage')

best.k2 = table2array(adp_lm.Coefficients(aaa,:));

elseif strcmp(adp_lm.CoefficientNames{aaa}, 'dldt^2')

best.k3 = table2array(adp_lm.Coefficients(aaa,:));

elseif strcmp(adp_lm.CoefficientNames{aaa}, 'dldt')

best.k4 = table2array(adp_lm.Coefficients(aaa,:));

end

end

bestr2 = adp_lm.Rsquared.Adjusted;

idx = ooo;

end

end

% plotting the results

figure(fig)

% makes sure the saved pdf fits within a landscape document

set(gcf,'PaperOrientation','landscape');

set(gcf,'PaperUnits','normalized');

set(gcf,'PaperPosition', [0 0 1 1]);

% stage and dldt

aej = fitlm(tbl, lroptions{idx})

plot(aej)

xticks = get(gca, 'xtick');

yticks = get(gca, 'ytick');

title({[strrep(rvname, '_', ' ') ':'], lroptions{idx}})

grid on

legend off

num_factors = length(regexp(aej.Formula.LinearPredictor, '+'))+1;

if num_factors == 1

text(mean(xticks), yticks(2), {...

[num2str(aej.Coefficients.Estimate(1)) '*' aej.CoefficientNames{1}];...

['Adjusted R^2 = ' num2str(aej.Rsquared.Adjusted)];...

['Ordinary R^2 = ' num2str(aej.Rsquared.Ordinary)];...

['p-value: ' aej.CoefficientNames{1} ' = ' num2str(aej.Coefficients.pValue(1))];...

plot_notes...

}, 'fontsize', 8)

elseif num_factors == 2

text(mean(xticks), yticks(2), {...

[num2str(aej.Coefficients.Estimate(1)) '*' aej.CoefficientNames{1}];...

[num2str(aej.Coefficients.Estimate(2)) '*' aej.CoefficientNames{2}];...

['Adjusted R^2 = ' num2str(aej.Rsquared.Adjusted)];...

['Ordinary R^2 = ' num2str(aej.Rsquared.Ordinary)];...

['p-value: ' aej.CoefficientNames{1} ' = ' num2str(aej.Coefficients.pValue(1))];...

['p-value: ' aej.CoefficientNames{2} ' = ' num2str(aej.Coefficients.pValue(2))];...

plot_notes...

}, 'fontsize', 8)

elseif num_factors == 3

text(mean(xticks), yticks(2), {...

[num2str(aej.Coefficients.Estimate(1)) '*' aej.CoefficientNames{1}];...

[num2str(aej.Coefficients.Estimate(2)) '*' aej.CoefficientNames{2}];...

[num2str(aej.Coefficients.Estimate(3)) '*' aej.CoefficientNames{3}];...

['Adjusted R^2 = ' num2str(aej.Rsquared.Adjusted)];...

['Ordinary R^2 = ' num2str(aej.Rsquared.Ordinary)];...

['p-value: ' aej.CoefficientNames{1} ' = ' num2str(aej.Coefficients.pValue(1))];...

['p-value: ' aej.CoefficientNames{2} ' = ' num2str(aej.Coefficients.pValue(2))];...

['p-value: ' aej.CoefficientNames{3} ' = ' num2str(aej.Coefficients.pValue(3))];...

plot_notes...

}, 'fontsize', 8)

elseif num_factors == 4

text(mean(xticks), yticks(2), {...

[num2str(aej.Coefficients.Estimate(1)) '*' aej.CoefficientNames{1}];...

[num2str(aej.Coefficients.Estimate(2)) '*' aej.CoefficientNames{2}];...

[num2str(aej.Coefficients.Estimate(3)) '*' aej.CoefficientNames{3}];...

[num2str(aej.Coefficients.Estimate(4)) '*' aej.CoefficientNames{4}];...

['Adjusted R^2 = ' num2str(aej.Rsquared.Adjusted)];...

['Ordinary R^2 = ' num2str(aej.Rsquared.Ordinary)];...

['p-value: ' aej.CoefficientNames{1} ' = ' num2str(aej.Coefficients.pValue(1))];...

['p-value: ' aej.CoefficientNames{2} ' = ' num2str(aej.Coefficients.pValue(2))];...

['p-value: ' aej.CoefficientNames{3} ' = ' num2str(aej.Coefficients.pValue(3))];...

['p-value: ' aej.CoefficientNames{4} ' = ' num2str(aej.Coefficients.pValue(4))];...

plot_notes...

}, 'fontsize', 8)

end

% writing summary table

if write_save~=0

labeler = strrep(rvname, '_', '');

fprintf(kscsv, ['%s,',... % '%s, %s',...

'%.2f +/- %.2f (%.2f; %.2e),',...

'%.2f +/- %.2f (%.2f; %.2e),',...

'%.2f +/- %.2f (%.2f; %.2e),',...

'%.2f +/- %.2f (%.2f; %.2e),',...

'%.2f\r\n'],...

labeler(1:3),...

best.k1,...

best.k2,...

best.k3,...

best.k4,...

bestr2);

end

% saving figures of all major axes

if save_fig ~= 0;

% *** SAVING FIGURE BEFORE MOVING ON

% makes sure the saved pdf fits within a landscape document

set(gcf,'PaperOrientation','landscape');

set(gcf,'PaperUnits','normalized');

set(gcf,'PaperPosition', [0 0 1 1]);

% save as .tif

print(gcf, '-dtiff' , [save_path 'USGS_Q_relate\Qrelation_' sitenum '_' strrep(rvname, '_', ' ') addnotes '.tif'])

end

fig = fig+1;

%% Saving the data

%7{

% if not M2, M5 or A5

labeler = strrep(rvname, '_', '');

eval([labeler(1:3) 'lr.data2Q = avgdata;'])

eval([labeler(1:3) 'lr.k_stage2 = best.k1(1);'])

eval([labeler(1:3) 'lr.k_stage = best.k2(1);'])

eval([labeler(1:3) 'lr.k_dldt2 = best.k3(1);'])

eval([labeler(1:3) 'lr.k_dldt = best.k4(1);'])

eval(['save([save_path ''' labeler(1:3) '_relate2Q.mat''], ''' labeler(1:3) 'lr'')'])

%}

end

end

%% closing .csv file

fclose(kscsv);

close all

%% determining time periods of use - comment out after all done

% close all

% rvname

% plotyy(data(:,1), data(:,2), data(:,1), data(:,3))

% set(gca, 'xticklabel', datestr(get(gca, 'xtick'),'mm-dd-yy'))

======== END FILE ========

File 5: This file imports the determined coefficients, pairs them with observations, and TCM observations to create the entire two-part tidal rating curve.

======== BEGIN FILE ========

%{

Creating a final rating curve that incorporates storm periods and tidal

baseflow conditions.

Author: Allan Jones

Date: 15 August 2017

%}

clear; close all; clc

% determining which computer is being used

cur_dir = cd; slashes = regexp(cur_dir, '\');

comp_using = cur_dir(1:slashes(3));

% save the entire discharge .csv

save_fig = -1;

write4HC = -1;

%% River characterization data

%%%%%%%%%%%%%%%%% - MISSION RIVER

% site cleaning thresholds - [qlow, qhi, slow, shi]

sites.M1.thresholds = [-35, 250, 0.75, 5.5];

sites.M2.thresholds = [-35, 250, 0.75, 5.5];

sites.M3.thresholds = [-20, 90, 1.00, 5.5];

sites.M4.thresholds = [-30, 90, 1.25, 5.5];

sites.M5.thresholds = [-50, 40, 2.2, 5.5];

% channel width in [meters]

sites.M1.width = 23.0;

sites.M2.width = 31.7;

sites.M3.width = 30.5;

sites.M4.width = 30.0;

sites.M5.width = 34.3;

%%%%%%%%%%%%%%%%% - ARANSAS RIVER

% site cleaning thresholds - [qlow, qhi, slow, shi]

sites.A1.thresholds = [-35, 250, 1.10, 2.7];

sites.A2.thresholds = [-35, 60, 1.10, 2.7];

sites.A3.thresholds = [-50, 90, 1.75, 3.1];

sites.A4.thresholds = [-50, 60, 1.75, 3.25];

sites.A5.thresholds = [-50, 40, 2.2, 3.75];

% channel width in [meters]

sites.A1.width = 17.0;

sites.A2.width = 27.9;

sites.A3.width = 40.5;

sites.A4.width = 51.7;

sites.A5.width = 68.7;

%% prepping output information

outputpath = [comp_using 'Desktop\toShare_dischargeData_v2\'];

% [comp_using 'Dropbox\Field Work\Analysis\'...

% 'Field Data Analysis\TCM rating curve\Figures\' date '\'];

% [comp_using 'Dropbox\LO_Methods_tidalRiverGaging\'...

% 'Writing_Docs\Tables & Figures\Final discharge files\' date '\'];

if ~isdir([outputpath])

mkdir([outputpath]);

end

% opening the summary document and writing header

Qsummary = fopen( [outputpath 'MA_discharge_summary.csv'],'w');

fprintf(Qsummary, '%s\r\n',...

'Mean (Median) +/- SE (min; max)');

fprintf(Qsummary, '%s, %s, %s, %s\r\n',...

'Site Name',...

'Baseflow',...

'Storm',...

'Overall');

%% import and clean site long-term datasets

% create a path for loading data

load_path = ['Dropbox\Field Work\Analysis\All Long Term Data\'...

'Matlab Cleaned Data\Calibrated TCM data\'];

data_saving = 'prelim_20170725\TCM matrix-only data\';

load_ks_path = [comp_using 'Desktop\toShare_dischargeData_v2\mat files\'];

% site identifiers

soi = {'A1', 'A2', 'A3', 'A4', 'A5','M1', 'M2', 'M3', 'M4', 'M5'};

avgwindow = 13;

%% obtaining deployment dates for cleaning

%opening the .csv deployment file

deployment_path = ['Dropbox\Field Work\Analysis\'...

'Field Data Analysis\Clean Field data\'];

deploy_file = 'TCM_deployments.csv';

Dfid = fopen([comp_using deployment_path deploy_file]);

dd = [];

%reading the information from each line

dummy = fgetl(Dfid); num = 1;

while ischar(dummy)

% pull dates from between commas and create variable of site name

commas = [1 regexp(dummy, ',') length(dummy)+1];

for iii = 1:length(commas)-1

if iii == 1

% creating variable (sturcture) of site name

sitevar = dummy(commas(iii):commas(iii+1)-1);

else

% storing the dates within the variable

eval(['dd.' sitevar '.deploy_dates{iii-1,1} = '....

dummy(commas(iii)+1:commas(iii+1)-1) ';'])

end

end

% grab new line

dummy = fgetl(Dfid); num = num+1;

end

fclose(Dfid);

%% load each site's data and calculate discharge

for vvv = 3 % 1:length(soi)

disp(['Loading site: ' soi{vvv}])

% loading saved TCM/LTC data

eval(['load([comp_using load_path data_saving '...

'soi{vvv} '...

'''_calib_mat_only.mat''])'])

% Making data ONE LARGE data variable

eval(['data = ' soi{vvv} '_mat_only;'])

all_data = [ nanmean([data(:,1), data(:,5)],2),... % averaged LTC and TCM timestamps

data(:,17),... % TCM velocities [cm s-1]

data(:,2)]; % LTC stage [m]

% remove bizarre points

eval(['threshs = sites.' soi{vvv} '.thresholds;'])

% velocities < -50 cm/s || > 90 cm/s

idx = find( (all_data(:,2) < threshs(1)) | (all_data(:,2) > threshs(2)) );

all_data(idx, :) = [];

% stages

idx = find( (all_data(:,3) < threshs(3)) | (all_data(:,3) > threshs(4)) );

all_data(idx, :) = [];

% clip to acceptable data

start = datenum('20150701 00:00', 'yyyymmdd HH:MM');

if strcmp(soi{vvv}, 'A3')

%*** does nothing, "find"s nothing

ender = datenum('20170204 00:00', 'yyyymmdd HH:MM');

idx = find( (all_data(:,1) < start) &...

(all_data(:,1) > ender) );

all_data(idx,:) = [];

% removing data before and after acceptable period

else

% clip data from before proper TCM installation

idx = find(all_data(:,1) < start);

all_data(idx,:) = [];

end

% store 'nan'-values for installation times

eval(['lenny = length(dd.' soi{vvv} '.deploy_dates);'])

for iii = 1:lenny

eval(['timestamp = datenum('...

'dd.' soi{vvv} '.deploy_dates{iii,1}'...

',''yyyymmdd HHMM'');'])

idx = find( (all_data(:,1) >= timestamp - 1/24) &...

(all_data(:,1) < timestamp + 2/24) );

all_data(idx,[2:3]) = nan;

end

% perform moving average on data

disp(['Moving average: ' soi{vvv}])

avgdata = nan( length(all_data), size(all_data,2)+1 );

avgdata(:,1) = aej_moving_avg_v1(all_data(:,1), avgwindow);

avgdata(:,2) = aej_moving_avg_v1(all_data(:,2), avgwindow);

avgdata(:,3) = aej_moving_avg_v1(all_data(:,3), avgwindow);

dldt = nan( size(avgdata(:,3)) );

for iii = 1:length(avgdata)-1

if avgdata(iii+1,1) - avgdata(iii,1) < 30/(60*24)

dldt(iii) = (avgdata(iii+1,3) - avgdata(iii,3))/...

(avgdata(iii+1,1) - avgdata(iii,1));

end

end

avgdata(:,4) = aej_moving_avg_v1(dldt, avgwindow);

% remove data without TCM?

idx = find( ~isnan(avgdata(:,2)) );

avgdata = avgdata(idx,:);

%% import saved linear model coefficients and calculate discharge

eval(['load([load_ks_path soi{vvv} ''_relate2Q.mat''])'])

eval(['lmks = ' soi{vvv} 'lr;'])

eval(['width = sites.' soi{vvv} '.width;'])

% creating storage variable

discharge_estimate = nan( length(avgdata), 2);

discharge_estimate(:,1) = avgdata(:,1);

% convert velocity to m/s

avgdata(:,2) = avgdata(:,2)./100;

% begin looping through timestamps and determining if baseflow or

% storm and calculate volumetric discharge appropriately

disp(['Calculating Volumetric Discharge: ' soi{vvv}])

percentile90_thresh = prctile(avgdata(:,2), 90);

idvect = zeros( size(avgdata(:,2)) );

for iii = 1:length(avgdata)

% storm periods

if avgdata(iii,2) >= percentile90_thresh % ||...

% avgdata(iii,4) >= (nanmean(avgdata(:,4)) + 3*nanstd(avgdata(:,4)))

% calculating the storm discharge

discharge_estimate(iii,2) = ...

avgdata(iii,2)*avgdata(iii,3)*width;

idvect(iii) = 1;

else % baseflow periods

% calculating the baseflow discharge

discharge_estimate(iii,2) = ...

lmks.k_stage2*avgdata(iii,3).^2 + ...

lmks.k_stage*avgdata(iii,3) + ...

lmks.k_dldt2*avgdata(iii,4).^2 + ...

lmks.k_dldt*avgdata(iii,4);

end

end

%% output summary table and dischrge .csvs

% writing the discharge data file

if write4HC > 0

disp(['Writing discharge .csv: ' soi{vvv}])

fid = fopen( [outputpath soi{vvv} '_Discharge.csv'], 'w');

fprintf(fid, '%s, %s\r\n',...

'Time', 'Discharge, [m3 s-1]');

% writing the actual data - float point

for iii = 1:length(discharge_estimate)

if ~isnan(discharge_estimate(iii,1))

fprintf(fid, '%s, %.4f\r\n',...

datestr(discharge_estimate(iii,1)),...

discharge_estimate(iii,2));

end

end

fclose(fid);

end

% writing the site line to the summary file

disp(['Adding line to summary file: ' soi{vvv}])

bi = find( idvect == 0 );

si = find( idvect == 1 );

if strcmp(soi{vvv}, 'A5') || strcmp(soi{vvv}, 'M2')

fprintf(Qsummary, '%s, %s, %s, %s\r\n',...

soi{vvv},...

'nan',...

'nan',...

'nan');

else

% writing bas

fprintf(Qsummary, ['%s, '... site name

'%.2f (%.2f) +/- %.2f (%.2f; %.2f), '... baseflow

'%.2f (%.2f) +/- %.2f (%.2f; %.2f), '... storm

'%.2f (%.2f) +/- %.2f (%.2f; %.2f)\r\n'],... overall

soi{vvv},... site name

nanmean(discharge_estimate(bi,2)),... mean (baseflow)

nanmedian(discharge_estimate(bi,2)),... median (baseflow)

nanstd(discharge_estimate(bi,2)),... std. error (baseflow)

nanmin(discharge_estimate(bi,2)),... minimum (baseflow)

nanmax(discharge_estimate(bi,2)),... maximum (baseflow)

nanmean(discharge_estimate(si,2)),... mean (storm)

nanmedian(discharge_estimate(si,2)),... median (storm)

nanstd(discharge_estimate(si,2)),... std. error (storm)

nanmin(discharge_estimate(si,2)),... minimum (storm)

nanmax(discharge_estimate(si,2)),... maximum (storm)

nanmean(discharge_estimate(:,2)),... mean (overall)

nanmedian(discharge_estimate(:,2)),... median (overall)

nanstd(discharge_estimate(:,2)),... std. error (overall)

nanmin(discharge_estimate(:,2)),... minimum (overall)

nanmax(discharge_estimate(:,2)) ); % maximum (overall)

end

if save_fig > 0;

figure(vvv)

ppp = plot(discharge_estimate(:,1), discharge_estimate(:,2));

set(ppp, 'marker', '.')

set(ppp, 'markersize', 12)

title(soi{vvv})

ylabel({'Discharge,','[$m^3$ $s^{-1}$]'},...

'Interpreter', 'Latex')

set(gca, 'xticklabel', datestr(get(gca, 'xtick'), 'mm/dd/yy'))

xlabel('Time, [mm/dd/yy]')

save_path = [comp_using 'Dropbox\LO_Methods_tidalRiverGaging\'...

'Writing_Docs\Tables & Figures\Figures\Supplement D tiffs\'];

% *** SAVING FIGURE BEFORE MOVING ON

% save as .tif

% print(gcf, '-dpdf' , [save_path soi{vvv} '_Q_timeseries.pdf'])

print(gcf, '-dtiff' , [save_path soi{vvv} '_Q_timeseries.tif'])

end

aej = find( ~isnan(discharge_estimate(:,2)) );

string = datestr(avgdata(aej(end),1));

QQQ = discharge_estimate(aej(end),2);

disp([soi{vvv} ': ' string '=> ' num2str(QQQ) ])

disp(['=========== END OF SITE ==========='])

end

% closing discharge summary file

fclose(Qsummary);

%% good checker script

%{

figure(1)

start = datenum('20151121', 'yyyymmdd');

ender = datenum('20151122', 'yyyymmdd');

subplot(3,1,1)

plot(discharge_estimate(:,1), discharge_estimate(:,2), '.')

hold on

set(gca, 'xlim', [start, ender])

set(gca, 'xticklabel', datestr(get(gca, 'xtick'), 'HH:MM')) %'mm/dd'))

subplot(3,1,[2:3])

plot(avgdata(:,1), avgdata(:,2), '.')

hold on

plot(avgdata(:,1), avgdata(:,3)/10,'.')

plot(avgdata(:,1), avgdata(:,4)/10,'.')

legend('Vel', 'Stage/10', '(dS/dt)/10')

set(gca, 'xlim', [start, ender])

set(gca, 'xticklabel', datestr(get(gca, 'xtick'), 'mm/dd'))

plot(get(gca, 'xlim'), [percentile90_thresh, percentile90_thresh], '--r')

% dl/dt checks...

figure(2)

aej = (nanmean(avgdata(:,4)) + 3*nanstd(avgdata(:,4)));

mmj = (nanmean(avgdata(:,4)) - 3*nanstd(avgdata(:,4)));

plot(avgdata(:,1), avgdata(:,4), '.')

hold on

plot(get(gca, 'xlim'), [aej, aej], '--r')

plot(get(gca, 'xlim'), [mmj, mmj], '--r')

plot(avgdata(:,1), avgdata(:,2)*10, '.')

for ddd = 99:0.001:100

if abs( prctile(avgdata(:,4), ddd) - (nanmean(avgdata(:,4))+3*nanstd(avgdata(:,4))) ) < 1e-5

disp(num2str(ddd))

break

end

end

%}

======== END FILE ========
